# Unraveling sauropod diversity in the Portezuelo Formation of Patagonia through a comprehensive analysis of new and existing material

**DOI:** 10.1186/s12862-024-02280-9

**Published:** 2024-07-09

**Authors:** Kevin Leonel Gomez, Agustín Pérez-Moreno, Jorge Gustavo Meso, Flavio Bellardini, Mattia Antonio Baiano, Diego Pol, Alberto Garrido, Jonatan Kaluza, Luciana Muci, Michael Pittman

**Affiliations:** 1 Consejo Nacional de Investigaciones Científicas y Técnicas (CONICET), Ciudad Autónoma de Buenos Aires, Buenos Aires, C1405 Argentina; 2 Instituto de Investigación en Paleobiología y Geología (IIPG), Universidad Nacional de Río Negro (UNRN), Consejo Nacional de Investigaciones Científicas y Técnicas (CONICET), General Roca, Río Negro Province 8332 Argentina; 3 División Paleontología de Vertebrados, Museo de La Plata (Anexo), Calle 122 y 60, La Plata, Buenos Aires Province B1900WA Argentina; 4 School of Life Sciences, The Chinese University of Hong Kong, Shatin, Hong Kong SAR China; 5 Área Laboratorio e Investigación, Museo Municipal ‘Ernesto Bachmann’. Dr Natali S/N, Villa El Chocon, Neuquén, 8311 Argentina; 6 Universidad Nacional de Río Negro, Sede Alto Valle/Valle Medio. Estados Unidos 750, General Roca, Río Negro Province 8332 Argentina; 7 Museo Argentino de Ciencias Naturales, Av. Angel Gallardo 470, Ciudad Autónoma de Buenos Aires, 1405 Argentina; 8 Museo Provincial de Ciencias Naturales, Dirección Provincial de Minería, Zapala, Neuquén Province Q8340 Argentina; 9 Fundación de Historia Natural Félix de Azara, Universidad Maimónides, Hidalgo 775, Ciudad Autónoma de Buenos Aires, Buenos Aires, C1405 Argentina

**Keywords:** Sauropod diversity, Portezuelo formation, Somphospondyli, Dinosauria, Cretaceous, Phylogeny

## Abstract

The Portezuelo Formation preserves an outstanding record of the upper Turonian – lower Coniacian. Despite the discovery of a significant quantity of sauropod fossil material from the formation, only two species have been formally described to date: *Malarguesaurus florenciae* and *Futalognkosaurus dukei*. Here we present new sauropod material mostly composed of non-articulated caudal vertebrae (MCF-PVPH 916 and 917) that belong to two titanosauriforms on the basis of the following features: anterior caudal vertebrae with procoelous-opisthoplatyan articulations, transverse processes that reach the posterior articular face of the centrum and neural spines with a transverse width of around 50% of their anteroposterior length; anterior and middle caudal vertebrae with the neural arch restricted to the anterior half of the centrum; middle caudal centra with circular cross-section. Phylogenetic analysis recovers the new material in close relation to *Malarguesaurus* within a monophyletic clade at the base of Somphospondyli. This clade shares large pedicel height with a vertical anterior border on the middle caudal vertebrae, a vertical orientation of the neural spines on the distalmost middle caudal vertebrae and proximalmost posterior caudal vertebrae, and subequal relative lengths of the proximal ulnar condylar processes. The specimens presented here are distinct not only from *Futalognkosaurus*, but also from other indeterminate titanosaurian remains from the same formation. However, there are no significant differences between the specimen MCF-PVPH 917 and *Malarguesaurus*, but there are differences between the posterior caudal vertebrae of MCF-PVPH 916 and *Malarguesaurus*, so they could be considered different species. Whilst we err on the side of caution in not naming new taxa here, the two specimens significantly expand what we know about sauropods in the Turonian–Coniacian ecosystems of Patagonia, which will continue to do so as more material is discovered.

**Supplementary Information:**

The online version contains supplementary material available at 10.1186/s12862-024-02280-9.

## Introduction

Sauropod dinosaurs, characterized by a herbivorous diet, elongated necks and tails, and relatively small skulls, were the largest terrestrial vertebrates to have ever inhabited the planet [1–3]. Serving as the dominant megaherbivores in terrestrial ecosystems throughout the Mesozoic, particularly in Gondwana [4–6], neosauropods ultimately diversified into Diplodocoidea and Macronaria, which were the exclusive representatives of sauropods during the Cretaceous [7, 8].

Diplodocoidea is a group of neosauropods known from the Middle Jurassic [9, 10] to the early Late Cretaceous (Turonian; [11, 12]), representing important modelers of both Laurasia and Gondwana terrestrial ecosystems for more than 60 million years [13–16]. The second group, Macronaria, would have originated in Asia during the Middle Jurassic [12]and would eventually became the dominant herbivorous lineage in the Late Cretaceous, when titanosaurians flourished worldwide [17–19]. The last diplodocoids, the rebbachisaurids, come from the Early Cretaceous of Gondwana and are observed until close to the Cenomanian–Turonian transition [20–22]. In this context, the reconstruction of the sauropod fauna composition from post-Turonian ecosystems contributes to understanding the macroevolutionary processes linked with the decline of the last diplodocoids and the rise of the titanosaurians.

In Patagonia, abundant vertebrate fossil specimens, especially archosaurs, have been found in the Portezuelo Formation (upper Turonian – lower Coniacian; [23, 24]), the second lithostratigraphic unit of the Río Neuquén Subgroup (Neuquén Group; [23, 24]). The earliest findings of dinosaurs from the Portezuelo Formation come from two sites in Neuquén Province, close to Plaza Huincul [25] and Barreales Lake [26]. Established by Keidel [27], the Portezuelo Formation overlies the Cerro Lisandro Formation and underlies the Los Bastos Formation [23], exhibiting good exposures towards Barreales Lake, with thicknesses ranging from 95 to 130 m [28, 29]. Its composition of medium-grained, yellowish and reddish-brown sandstones indicates a fluvial deposition regime, alternating with orange pelites in a decreasing grain size sequence, which become very thin in the neighboring province of Río Negro. It presents frequent paleosoils, evidence of stable environmental conditions.

The Sierra del Portezuelo area in the Neuquén Basin of Patagonia, Argentina, is pivotal for the study of Late Cretaceous sauropod dinosaurs. Within this Formation, only two formally described taxa have emerged: *Malarguesaurus florenciae* [30] and *Futalognkosaurus dukei* [31]. In this unique paleontological landscape, a field expedition in February 2023 yielded crucial materials, primarily comprising isolated sauropod caudal vertebrae from two distinct specimens separated by approximately 300 m. These findings, retrieved from the lower section of the Turonian–Coniacian Portezuelo Formation, present a compelling opportunity to expand our understanding of the neosauropods that once roamed this ancient Patagonian ecosystem.

In the following sections, we present a comprehensive analysis of the anatomical characteristics, taxonomic implications, and phylogenetic relationships of the sauropod specimens from the Sierra del Portezuelo area, offering a nuanced perspective on the evolutionary tapestry of these colossal creatures that once dominated the landscapes of ancient Patagonia.

## Institutional abbreviations

**BYU**, Brigham Young University, Museum of Paleontology, Provo, Utah, USA; **CM**, Carnegie Museum of Natural History, Pittsburgh, USA; **IANIGLA**, Instituto Argentino de Nivología, Glacialogía y Ciencias Ambientales, Mendoza, Argentina; **MCF**, Museo Carmen Funes, Plaza Huincul, Neuquén, Argentina; **MNN**, Musée National du Niger, Niamey, Republic of Niger; **MMS**, Museo Municipal de Ciencias Naturales, Senillosa, Neuquén, Argentina; **MPM**, Museo Padre Molina, Río Gallegos, Santa Cruz, Argentina; **MUC**, Museo de la Universidad Nacional del Comahue, Neuquén, Argentina.

## Materials and methods

**Fig. 1:**
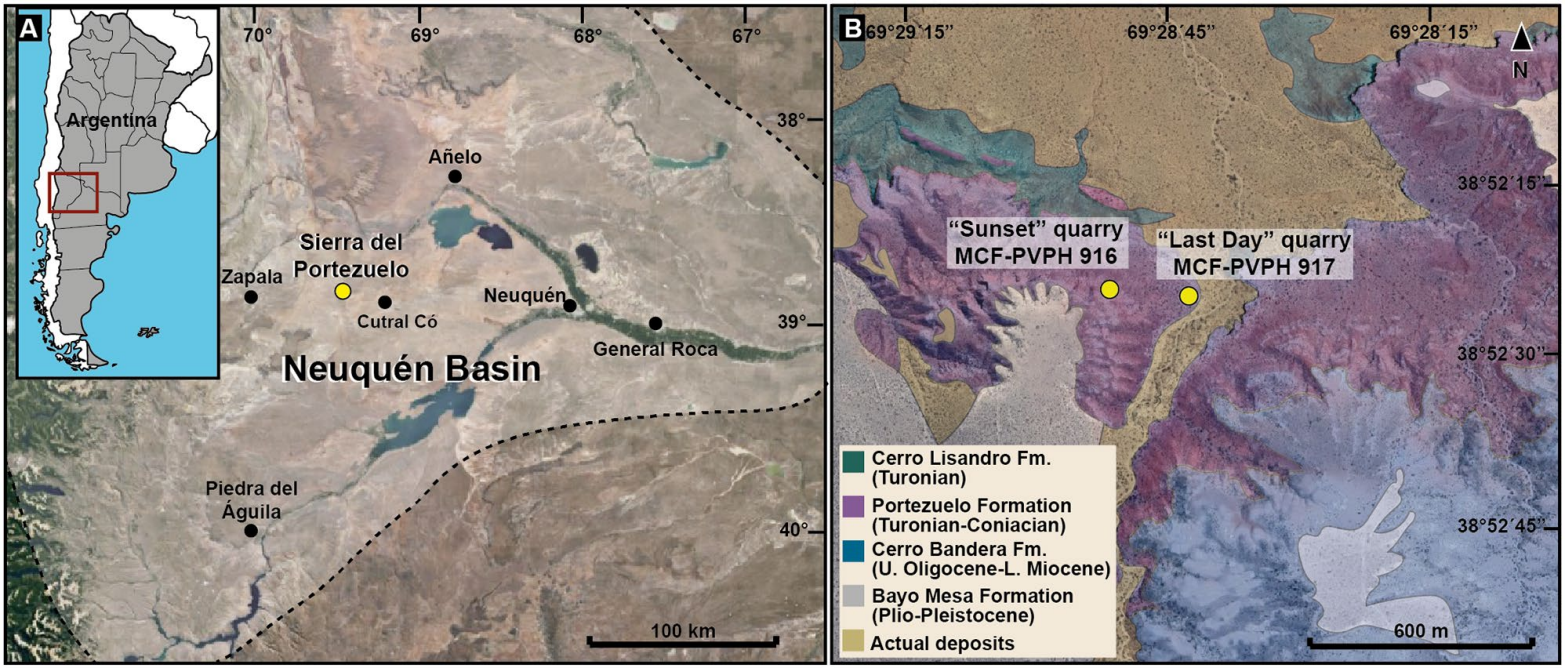
Location and geological map showing the distribution of the new titanosauriform specimens described here (MCF-PVPH 916 and 917) from the Portezuelo Formation. **A**, Neuquén Basin; **B**, detail of the fossiliferous localities (marked with yellow dots) from which the materials of the present study arise. Location map based on satellite image acquired from Google Earth (December 23, 2023; Data SIO, NOAA, US Navy, GEBCO; Image Landsat/Copernicus)

During February 2023, a field expedition was carried out in the Sierra del Portezuelo area, about 22 km northwest of the city of Cutral Có in Neuquén province (Fig. 1). This recovered isolated axial and appendicular bones belonging to two different specimens found around 300 m apart. Both were collected from the lower section of the Portezuelo Formation (Río Neuquén subgroup, Neuquén Group) of estimated upper Turonian – lower Coniacian age [23, 32]. Despite the poorer preservation of the caudal vertebrae and uncertainty in the exact positions of the elements in the original caudal vertebrae sequence, they represent anterior, middle and posterior zones of the tail. All bones were mechanically prepared and housed in the paleontological collections of the MCF.

For the osteological description we mainly followed the nomenclature of [33, 34], and [35]. The elongation of the caudal vertebrae was calculated according to the Elongation Index (EI) *sensu* [36] as the anteroposterior length of the centrum divided by the midline height of the posterior articular surface.

In order to investigate the phylogenetic relationships of MCF-PVPH 916 and 917, an equally weighted parsimony analysis was carried out. We added the new material to the [37] dataset which was then analyzed in *TNT v1.5* [38]. We conducted the analysis with the “New Technology Search”, using the command “xmult = hits50”. Under this command, Sectorial Search, Ratchet, Drift, and Tree Fusing algorithms are applied together with the traditional search procedures, such as Wagner Trees, Tree Branch Reconnection (TBR) and Subtree-Pruning-Regrafting algorithms, to find the Minimum Length Trees (MLTs).Using the most parsimonious trees (MPTs) held in the memory of the software, a subsequent “Traditional Search” was conducted through a round of TBR branch swapping. To identify unstable ‘wildcard’ taxa causing polytomies, we applied the Iter PCR methodology in *TNT* [39]. To assess branch support, we calculated three support metrics. Bremer support was calculated in *TNT* using first all 104 taxa and all most parsimonious trees. The search for suboptimal trees was performed by saving up to 1000 trees up to 1 step longer, increasing the score by 1 at a time. For groups not lost in suboptimal trees, the search with restrictions was repeated 3 times and the minimum score was used. Secondly, a Bremer Support was performed without the unstable taxa under the same parameters as above. The absolute Bootstrap and Jackknife support values were calculated in *TNT* with standard replacement using 1000 replicates.

### Systematic paleontology

Dinosauria Owen, 1842.

Sauropoda Marsh, 1878.

Neosauropoda, Bonaparte, 1986.

Macronaria Wilson and Sereno, 1998.

Titanosauriformes Salgado, Coria and Calvo, 1997.

Somphospondyli Wilson and Sereno, 1998.

Gen. et sp. Indet.

### Referred materials

Five non-articulated caudal vertebrae from the middle–posterior zone of the tail belonging from a single individual MCF-PVPH 916, and three isolated anterior–middle caudal vertebrae, a proximal portion of an ulna, and a metacarpal belonging to specimen MCF-PVPH 917. Despite being at the same level of the Portezuelo Formation, the distance between both sets of material is approximately 300 m, so we consider that they come from different individuals. As such for descriptions and comparisons, as well as the phylogenetic analysis, these specimens are treated separately as MCF-PVPH 916 and MCF-PVPH 917.

### Geological setting

The fossil bones described here come from the Sierra del Portezuelo, 22 km northwest of the city of Cutral Có, Neuquén Province, Patagonia, Argentina (Fig. 1). Elements of specimen MCF-PVPH 917 come from the ‘Last Day’ quarry (38◦52′25.5 S/69◦28′38.3 W; Fig. 1), and specimen MCF-PVPH 916 was found in situ in the ‘Sunset’ quarry (38◦52′24.4 S/69◦28′51.1 W; Fig. 1). Both specimens come from the lower levels of the Portezuelo Formation (upper Turonian–lower Coniacian), Río Neuquén Subgroup, Neuquén Group.

## Results

### Caudal vertebrae

The description is organized from anterior to posterior position along the caudal series.

#### Specimen MCF-PVPH 917

**Fig. 2:**
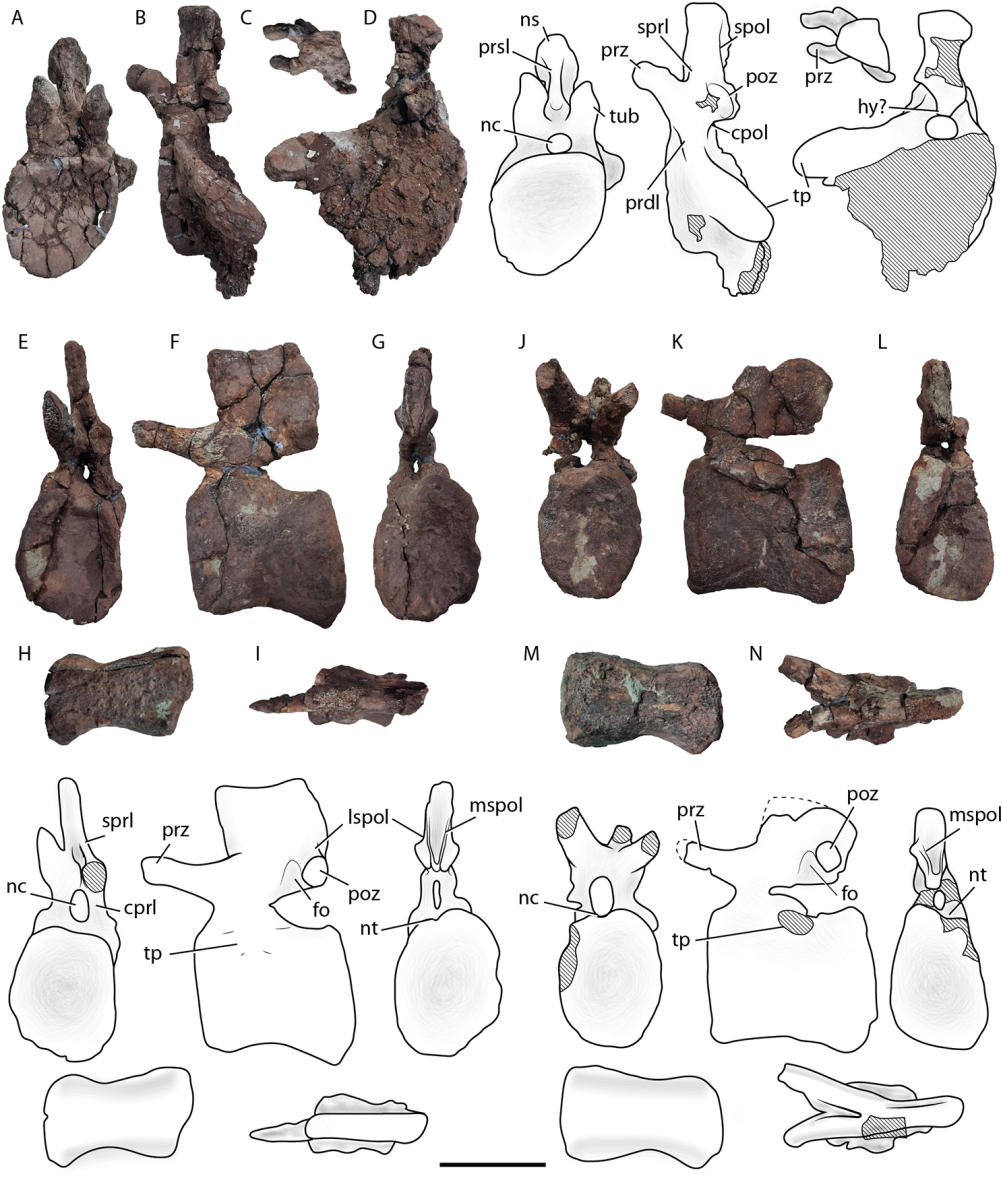
Caudal vertebrae from the Last Day locality, Sierra del Portezuelo area, Neuquén Province, Argentina. Anteriormost caudal vertebra (MCF-PVPH 917/1) in anterior (**A**), left lateral (**B**), dosal (**C**), and posterior (**D**) views; middle caudal vertebra (MCF-PVPH 917/2) in anterior (**E**), left lateral (**F**, inverted), posterior (**G**), ventral (**H**), and dorsal (**I**) views; middle caudal vertebra (MCF-PVPH 917/3) in anterior (**J**), left lateral (**K**), posterior (**L**), ventral (**M**), and dorsal (**N**) views. *Abbreviations* cpol, centropostzygapophyseal lamina; dl, dorsal lip; fo, fossa; hy, hyposphene; lspol, lateral spinopostzygapophyseal lamina; mspol, medial spinopostzygapophyseal lamina; nc, neural canal; ns, neural spine; nt, notch; poz, postzygapophysis; prdl, prezygodiapophyseal lamina; prsl, prespinal lamina; prz, prezygapophysis; spol, spinopostzygapophyseal lamina; sprl, spinoprezygapophyseal lamina; tp, transverse process; tub, tubercle. Dashed line for the reconstructed parts, and hatched pattern for broken surfaces. Scale bar of 10 cm

This comprises of three isolated caudal vertebrae, including one anterior and two middle caudal vertebrae (Fig. 2). Only one belongs to the most anterior section of the tail, probably one of the first three elements (MCF-PVPH 917/1; Fig. 2A-D). This vertebra is incomplete, lacking its right transverse process, and with most of the posterior surface eroded. The centrum of this vertebra is dorsoventrally taller than anteroposteriorly long, with a slightly concave anterior articular surface; the posterior articular surface is poorly preserved. Considering that the anterior articular surface does not have a deep concavity typical of vertebrae classified as procoelous, and that the middle caudal vertebrae of the same specimen are amphicoelous (MCF-PVPH 917/2 and MCF-PVPH 917/3; Fig. 2E-N), it is likely that the posterior face of MCF-PVPH 917/1 was slightly convex or flat, as in the procoelous-opisthoplatyan (see [40]) anterior vertebrae of *Malarguesaurus* [30]. In contrast, derived titanosaurians have procoelous anterior caudal vertebrae (e.g., Aelosaurini, Saltasaurinae, and Rinconsauria). The slightly concave anterior surface also differs from the deeper concave surface present in anterior caudal vertebrae of a titanosaurian from the same formation (MMS-PV 09 and MMS-PV 10; [41]). The lateral surface of the anteriormost caudal vertebra lacks fossae or ridges (Fig. 2B). This condition contrasts with the anterior caudal vertebrae with fossae or pits observed in many diplodocoids and some titanosauriforms [42, 43]. Also, the internal bone structure lacks of camerae or camellae in both the centrum and the neural arch, as in others early-branching somphospondylans (e.g., *Padillasaurus, Chubutisaurus*, *Huabeisaurus*, and *Wintonotitan*; [44–47]), which contrast with derived titanosaurians where caudal pneumaticity is present [48]. The lateral and ventral surfaces of the centrum are anteroposteriorly concave. The left preserved transverse process of MCF-PVPH 917/1 is posterolaterally oriented and transversely long, reaching the posterior face of the centrum (Fig. 2B), as in most titanosauriforms [49]. This transverse process tapers distally, in contrast to the wing-shaped transverse process of the anteriormost caudal vertebrae of diplodocoids [42]. A low prezygodiapophyseal lamina (PRDL) is present, faded anterodorsally to contact ventrally the prezygapophyseal process (Fig. 2B). The presence of this lamina reinforces the interpretation of this element as an anterior caudal vertebra. The neural arch is around 1.4 times as high as the centrum and located in the anterior half of the dorsal surface of the latter. This ratio is similar to that in *Malarguesaurus* ([30]: Fig. 5). The neural spine is vertical and as lateromedially wide as anteroposteriorly long (Fig. 2C), having a flat dorsal margin in lateral view. This is different from the neural spine of the anterior caudal vertebra of *Malarguesaurus*, which is slightly curved posteriorly, is anteroposteriorly longer than lateromedially wide, and has a dorsal margin that is convex in its anterior half and concave in its posterior half in lateral view [30]. The prezygapophyseal processes are robust and surpass the anterior face of the centrum (Fig. 2B). They are anterodorsally oriented with an angle of 45°, as in several titanosaurians (e.g., *Aeolosaurus*, *Overosaurus*, and *Narambuenatitan* [50–52]: Fig. 2). They are also curve slightly downward at their distal ends and project medially. On the lateral surface of the prezygapophyseal process there is a faint tubercle (Fig. 2A). The spinoprezygapophyseal laminae (SPRLs) are short and fade close to the base of the lateral surface of the neural spine. The sprl process is absent, whereas is present in some titanosauriforms [53]. A PRSL is present as a long and broad lamina on the anterior surface of the neural spine (Fig. 2A). There is no spinodiapophyseal lamina (SPDL), anterior centrodiapophyseal lamina (ACDL), or posterior centrodiapophyseal lamina (PCDL), as in MMS-PV 09 and MMS-PV 10 [41]. The postzygapophyseal facets are concave and oval in outline, and face ventrolaterally (Fig. 2D). Each postzygapophysis is supported ventrally by a broad centropostzygapophyseal lamina (CPOL) that also forms the lateral and dorsal margins of the neural canal. The poorly preserved posterior portion of the neural arch does not allow us to confirm the existence of a hyposphene. Both spinopostzygapophyseal laminae (SPOLs) are well developed and run dorsally along the full length of the posterior surface of the neural spine (Fig. 2B, D).

The middle caudal vertebra MCF-PVPH 917/2 is complete, aside from the left prezygapophyseal process, and is slightly deformed to the right side (Fig. 2E-I). The centrum is longer than high, and both anterior and posterior faces are slightly taller than they are wide (Table 1). Due to deformation, the maximum mediolateral width is placed in the central half of the articular surface (Fig. 2E, G). The centrum is slightly amphicoelous with its anterior articular surface more concave than the posterior one. This is shared with *Malarguesaurus* [30] and another specimen from the Sierra del Portezuelo area (MCF-PVPH 162; [54]) and contrasts with the typical procoelous middle caudal vertebrae of titanosaurians and specimen MCF-PVPH 163 [54], also from the same locality. The cross section of the centrum is circular, as in most titanosauriforms, and thereby differs from the trapezoidal shape seen in several lognkosaurians (e.g., *Uberabatitan*, and *Baurutitan*; [55]; [56]). The ventral surface of the centrum is concave in lateral view (Fig. 2F) and lacks a shallow longitudinal hollow (Fig. 2H), as in most neosauropods (e.g., *Camarasaurus*, *Europasaurus*, *Chubutisaurus*, *Lusotitan*, and *Lourinhasaurus*; BYU 9047 [5, 45, 57, 58]). On the dorsal margin of the posterior articular surface there is a notch (Fig. 2G), which is also observed in the specimen MCF-PVPH 916 (see below). The transverse processes are reduced to a low protuberance (Fig. 2F). As in Titanosauriformes, the neural arch of the middle caudal vertebra is placed in the anterior half of the dorsal surface of the centrum (e.g., *Giraffatitan*, *Venenosaurus*, *Tastavinsaurus*, and *Dreadnoughtus*; [59]; [40]; [60]; [61]). The height of the pedicels (below the level of prezygapophyses) is greater than that of other titanosauriforms (e.g., *Lusotitan*, *Venenosaurus*, and *Cedarosaurus*; [57]; [40]; [62]) but slightly less than the height observed in *Malarguesaurus* ([30]: Fig. 6). The neural spine is transversely thicker and rectangular in lateral view, being 1.5 times as long as high (Fig. 2F, I). The dorsal margin of the neural spine is slightly concave. As in *Malarguesaurus*, *Tastavinsaurus* [60], *Epachthosaurus*, and some Saltasauroidea (e.g., *Malawisaurus* and *Alamosaurus*; [63]; [64]), the neural spine is vertical (Fig. 2F), contrasting with the slightly directed posteriorly neural spine in the middle caudal vertebrae of most neosauropods. The prezygapophyseal process is nearly horizontal and projects beyond the anterior face of the centrum. Ventrally, this process is supported by a thick centroprezygapophyseal lamina (CPRL) that forms the lateral walls of the neural canal (Fig. 2E). The prezygapophyseal process is less than 40% of the anteroposterior length of the centrum, which is different from the elongated process of some titanosaurian taxa (e.g., *Epachthosaurus*, *Malawisaurus*, *Mendozasaurus*, and *Bonitasaura*; [65]; [66]; [67]). The postzygapophyseal facet is circular and flat (Fig. 2G) and a deep fossa develops anteriorly to it in the lateral surface of the neural arch (Fig. 2F). This fossa seems to be also present in *Malarguesaurus* (IANIGLA-PV 110/3). In posterior view, there are two laminae around the postzygapophyses, one lateral and one medial. Both connect the postzygapophyses with the neural spine, thus we consider them as the lateral spinopostzygapophyseal lamina (LSPOL) and the medial spinopostzygapophyseal lamina (MSPOL; Fig. 2G). This arrangement of laminae is similar to that observed in a slightly more anterior caudal vertebra of *Malarguesaurus* ([30]).

The middle caudal vertebra MCF-PVPH 917/3 represents the most posterior element of the caudal series of the Last Day locality. It is damaged as it lacks the distal tips of its prezygapophyseal processes (Fig. 2J), and the anterior end of the neural spine (Fig. 2J-N). The features of this vertebra are similar to those of the MCF-PVPH 917/2, except for a lesser development of the transverse processes (of which only the base of the left is preserved) and the more posterior orientation of the neural spine (Fig. 2K). In this vertebra, a lateral fossa also develops in the neural arch, in front of the postzygapophyses (Fig. 2K), and an MSPOL above and medial to them is also present (Fig. 2L). Despite being somewhat damaged, a slight notch is recognized on the dorsal margin of the posterior articular surface (Fig. 2L).

#### Specimen MCF-PVPH 916

**Fig. 3:**
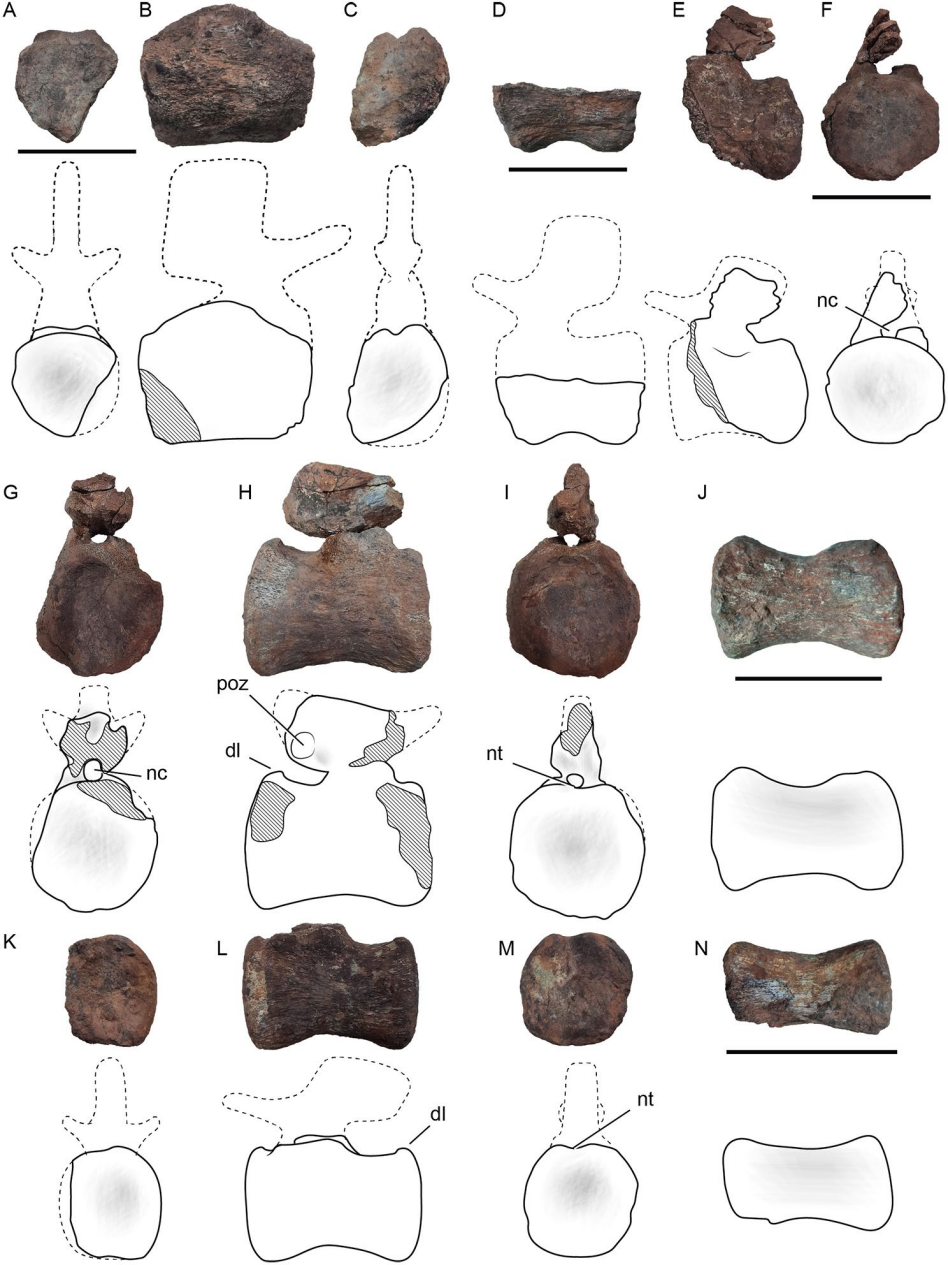
Caudal vertebrae from the Sunset locality, Sierra del Portezuelo area, Neuquén Province, Argentina. Middle caudal vertebra (MCF-PVPH 916/1) in anterior (**A**), right lateral (**B**) and posterior (**C**) views; middle caudal vertebra (MCF-PVPH 916/2) in left lateral (**D**) view; middle caudal vertebra (MCF-PVPH 916/3) in left lateral (**E**) and posterior (**F**) views; middle–posterior caudal vertebra (MCF-PVPH 916/4) in anterior (**G**), right lateral (**H**), posterior (**I**), and ventral (**J**) views; posterior caudal vertebra (MCF-PVPH 916/5) in anterior (**K**), left lateral (**L**), posterior (**M**), and ventral (**N**) views. *Abbreviations* dl, dorsal lip; nc, neural canal; ns, neural spine; nt, notch; poz, postzygapophysis. Dashed line for the reconstructed parts, and hatched pattern for broken surfaces. Scale bars of 10 cm

This specimen is composed of three middle and two posterior caudal vertebrae (Fig. 3). All vertebrae from this specimen are very damaged. The first two middle caudal vertebrae are represented by incomplete centra. The middle caudal vertebra MCF-PVPH 916/1 (Fig. 3A-C) is amphicoelous with its anterior face slightly deeper than the posterior one, which contrasts with the procoelous middle caudal vertebra of titanosaurians (e.g., [67–69]. Due to the poor preservation of this specimen, no transverse processes and chevron facets are recognized. The vertebra MCF-PVPH 916/2 is a ventral half portion of a centrum (Fig. 3D). As in MCF-PVPH 916/1, the centrum of MCF-PVPH 916/2 is amphicoelous, with the anterior articular surface more concave than the posterior one. The ventral surface is markedly concave in lateral view, and transversely convex. There are no excavations or ridges on the lateral surfaces of this centrum. It also lacks the ventrolateral ridges and midline hollow that is considered as a synapomorphy of Titanosauria [42, 57, 70]. On the ventral surface there are facets for the articulation with the chevrons.

The caudal vertebra MCF-PVPH 916/3 lacks the anterior portion of the centrum and neural spine (Fig. 3E-F). The posterior articular surface of the centrum is slightly concave, and the neural arch would be located at the anterior end of the dorsal surface of the centrum, as in the middle caudal vertebrae of titanosauriforms [30]. The posterior articular surface is slightly wider than high, although due to the state of preservation of the vertebra these dimensions could have been almost the same (Table 1). The transverse processes are slightly marked on the lateral surface of the centrum (Fig. 3E).

The caudal vertebra MCF-PVPH 916/4 could belong to the middle–posterior section of the tail (Fig. 3G-J). This element lacks portions of the centrum margins, both prezygapophyseal processes, and the posterior portion of the neural spine. The centrum is longer than high, having an elongation index (EI, *sensu* [36]) of 1.3. Both anterior and posterior articular surfaces have similar measurements, being as wide as they are tall (Fig. 3G, I; Table 1). The lateral surfaces lack fossae or ridges (Fig. 3H, J). As in the more anterior vertebrae no transverse processes are developed (Fig. 3H). The dorsal margin of the posterior articular surface is lipped dorsally in lateral view, and has a concave notch half way along its mediolateral width (Fig. 3 H-I). As in the specimen MCF-PVPH 917, the neural arch is placed in the anterior half of the dorsal surface of the centrum. Given the morphology of the preserved portion of the neural spine, if it was complete, it would be posteriorly inclined, which, together with the absence of transverse processes, and a greater elongation of the centrum, would indicate a more posterior position in the caudal series. The postzygapophyses are flat, have a circular outline (Fig. 3H), and do not surpass the posterior surface of the centrum.

**Table 1:** Measurements of caudal vertebrae MCF-PVPH 916 and 917 from the Portezuelo formation

Specimen	cl	aw	ah	pw	ph	sl	sw	sh	EI
MCF-PVPH 917/1	-	17,5	17,5	-	-	8,0	-	10,5	-
MCF-PVPH 917/2	12,5	9,5*	11,5	9,5	11,5	9,0	2,0	6,0	1,1
MCF-PVPH 917/3	12,5	9*	11,5	9*	11,5	8,5	2,0	-	1,1
MCF-PVPH 916/1	12,5	-	-	-	-	-	-	-	-
MCF-PVPH 916/2	13,0	-	-	-	-	-	-	-	-
MCF-PVPH 916/3	-	-	-	10,5	9,5	-	-	-	-
MCF-PVPH 916/4	11,0	9,0	8,5	9,0	8,5	-	-	-	1,3
MCF-PVPH 916/5	10,5	-	7,5	7,5	7,5	-	-	-	1,4

*Abbreviations* ah, anterior height of the centrum; aw, anterior width of the centrum; cl, centrum length; EI, elongation index *sensu* [36]; ph, posterior height of the centrum; pw, posterior width of the centrum; sh, height of the neural spine from the zygapophyses; sl, anteroposterior length of the neural spine; sw, width of the neural spine. All measurements are in centimeters

* indicates that a measurement is estimated

The caudal vertebra MCF-PVPH 916/5 is represented by a nearly complete isolated centrum without the neural arch (Fig. 3K-N). The centrum is slightly amphicoelous, and the anterior and posterior articular surfaces are equally concave. This contrasts with the procoelous posterior caudal vertebrae of *Malarguesaurus* ([30]) and the stronger procoely present in Eutitanosauria. The centrum is more elongated, having an EI of 1.4 (Table 1), which is different from the very elongated centrum of diplodocoids such as *Apatosaurus* (CM 3018), *Lavocatisaurus* [16], and *Nigersaurus* (MNN GAD 512). The articular surfaces have a circular outline (Fig. 3K, M), contrasting with some titanosaurians that have dorsoventrally flattened posterior caudal centra (e.g., *Saltasaurus*, and *Rinconsaurus*; [72]; [68]). No lateral ridges, ventral hollow, or transverse processes are present (Fig. 3L, N). As was described in specimen MCF-PVPH 917, the dorsal margin of the posterior articular surface has a notch half way along its mediolateral width, and is also lipped dorsally (Fig. 3L and M).

### Ulna

**Fig. 4:**
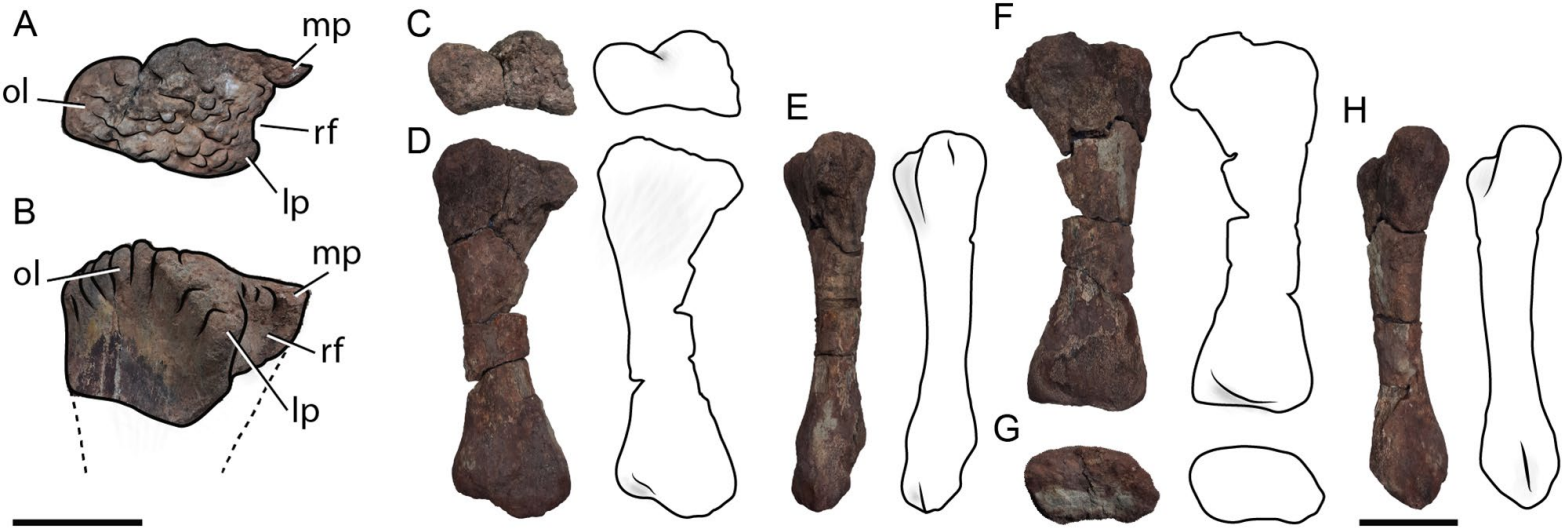
Ulna and metacarpal IV from the Last Day locality, Sierra del Portezuelo area, Neuquén Province, Argentina. Right ulna (MCF-PVPH 917/4) in proximal (**A**) and posterolateral (**B**) views; **C-H**, right metacarpal IV in proximal (**C**), lateral (**D**), anterior (**E**), medial (**F**), distal (**G**), and posterior (**H**) views. *Abbreviations* lp, lateral process; mp, medial process; ol, olecranon process; rf, radial fossa. Dashed line for the reconstructed parts. Scale bars of 10 cm

Only the proximal portion of a right ulna was recovered from the Last Day locality (MCF-PVPH 917/4; Fig. 4A-B). The cross section of the proximal diaphysis is mediolaterally compressed. In proximal view, the articular surface is triradiate (Fig. 4B), due to the well-developed medial, lateral, and posterior processes, as occurs in all sauropods [33]. The proximal processes are subequal in length, with the medial one being slightly longer, as in *Tehuelchesaurus* [73] and *Dreadnoughtus* [61]. This is different to the unequal length of the proximal processes of the ulna of several Neosauropoda, where the medial process is noticeably longer (e.g., *Europasaurus*, *Sauroposeidon*, *Neuquensaurus*, and *Bonitasaura*; [74]; [75]; [76]; [67]). The lateral process is more robust than the medial process (Fig. 4B). Unlike the prominent olecranon process of Saltasauroidea, in MCF-PVPH 917/4 this is low, barely projecting above the proximal surface (Fig. 4A). The dorsal development of the olecranon process also appears to be less than that described in another sauropod from the Portezuelo Formation ([41]). The radial fossa is wide and deep and is defined by the lateral and medial processes (Fig. 4B).

### Metacarpal

Only one metacarpal was collected from the Last Day locality (MCF-PVPH 917/5; Fig. 4C-H). This element is almost complete, and we tentatively interpret it as a right metacarpal IV. Its proximodistal length is 34.5 centimeters, and it expands anteroposteriorly at both ends (Fig. 4D), being longer anteroposteriorly at its proximal end (13.5 centimeters) than at its distal end (11.5 centimeters). Its proximal surface is rough, and anteroposteriorly expanded with respect to the diaphysis, being longer anteroposteriorly than wide mediolaterally, and slightly wider anteriorly than posteriorly (Fig. 4C). In lateral view, the proximal surface is flat and inclined anteroventrally. The lateral surface of the proximal portion is slightly concave to receive metacarpal V (Fig. 4C). This smooth depression occupies almost half the length of the shaft, indicating a tighter arrangement of the metacarpals, an indicator of a typical columnar posture of sauropods with massive bodies. The diaphysis is elliptical in cross section, being longer anteroposteriorly (6.5 centimeters) than it is wide mediolaterally (4.5 centimeters). The anterior margin is more concave than the posterior one. Its distal surface is longer anteroposteriorly than wide transversely, having a trapezoidal outline (Fig. 4G).

### Phylogenetic analysis

**Fig. 5:**
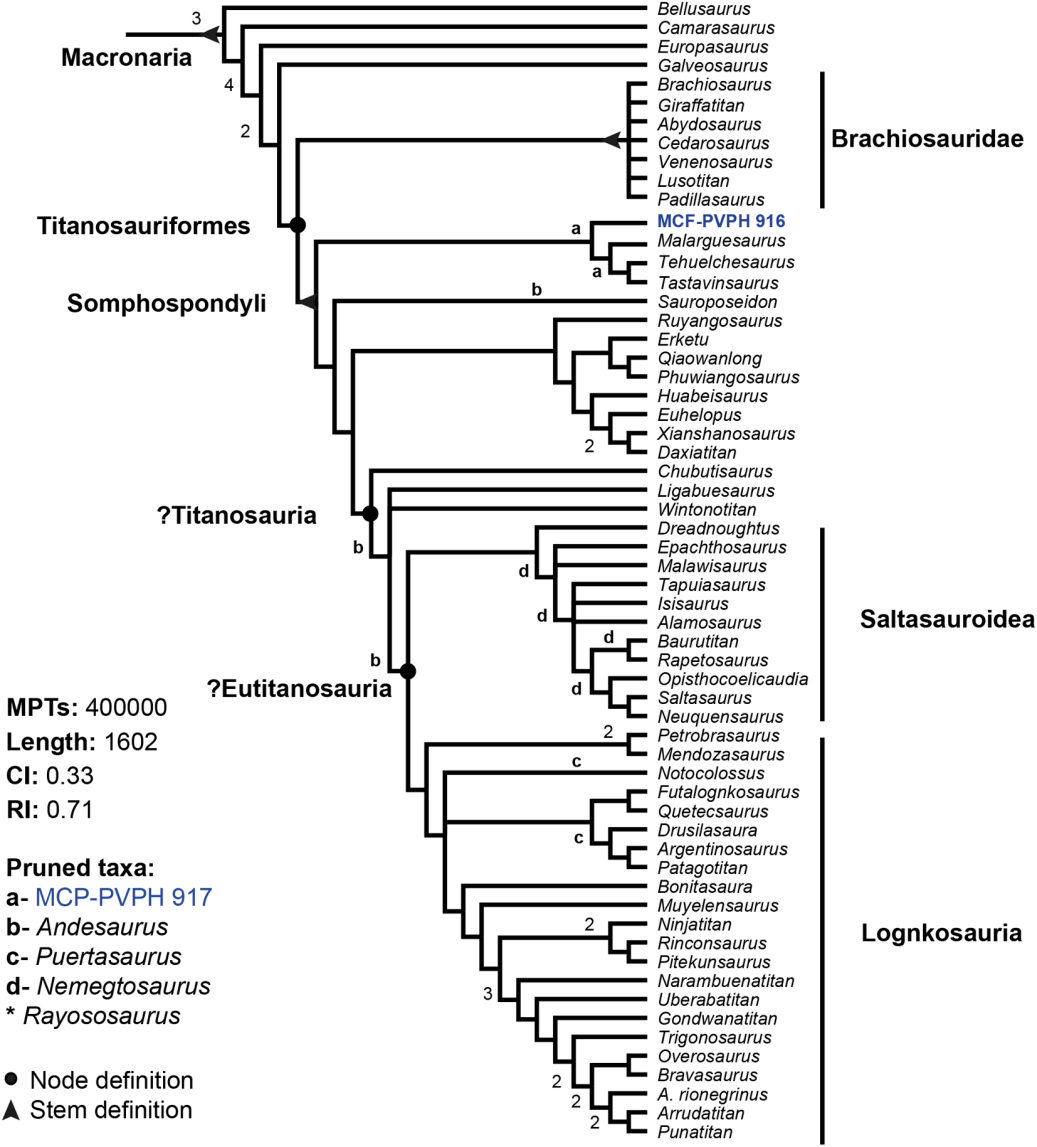
Simplified reduced strict consensus tree after pruning 4 unstable taxa and deleting repeated trees from the 400,000 trees obtained (see text for details). The alternative positions of *Rayososaurus* are found within Rebbachisauridae (not included in the figure). Support values are indicated in each node (Bremer support values higher than 1). *Abbreviations* CI, consistency index; MPT, most parsimonious tree; RI, retention index

The initial analysis using the data set of [37] retrieved 194 most parsimonious trees (MPTs) of 1602 steps. The second round of TBR branch swapping found 400,000 MPTs, resulting in an overflow of the memory tree space (consistency index = 0.33; retention index = 0.71). The strict consensus tree (Additional file: Figure S1) had the same polytomies seen in previous iterations of the phylogenetic dataset ([37, 77]). Iter PCR identified the neosauropods *Andesaurus*, *Puertasaurus*, *Nemegtosaurus*, *Rayososaurus*, and the specimen MCF-PVPH 917 as unstable taxa that were pruned to give 42,857 MPTs. This allowed the internal nodes Eutitanosauria, Titanosauria and Lithostrotia to be resolved (Fig. 5).

An early-branching clade of somphospondylans (Somphospondyli *sensu* [33]), consisting of *Tastavinsaurus*, *Tehuelchesaurus*, *Malarguesaurus* and specimens MCF-PVPH-916 and MCF-PVPH 917 was identified in our analysis. This clade is a polytomy that is only resolved when specimen MCF-PVPH 917 is pruned (Fig. 5). Members of this newly recognized clade of somphospondylans (*Tastavinsaurus*, *Tehuelchesaurus*, *Malarguesaurus*, and the specimens MCF-PVPH 916 and 917) share specific morphological characteristics, such as a large pedicel height below the prezygapophysis with a vertical anterior border on the middle caudal vertebrae (ch. 256), vertical orientation of the neural spines on the posterior-most anterior and middle caudal vertebrae (ch. 257), and first posterior caudal vertebrae with vertical neural spines (ch. 260). Specimen MCF-PVPH 916 differs from *Malarguesaurus* + (*Tastavinsaurus* + *Tehuelchesaurus*) by having amphiplatyan posterior caudal centra (ch. 261).

Bremer support values show a support of 1 for most nodes, including the clade to which specimens MCF-PVPH 916 and 917 belong. Some nodes with values greater than 1 correspond to clades such as Neosauropoda, Diplodocidae, Dicraeosauridae and Macronaria, although there are also other unnamed monophyletic groups with a support higher than 1 (Fig. 5; Additional file: Figures S2-S3). When analyzing the Bremer support without the influence of unstable taxa, the results are very similar, although other nodes with a support greater than 1 stand out, such as the clade corresponding to Saltasaurinae (Additional file: Figure S3). Regarding the Jackknife and Bootstrap analyses (Additional file: Figures S4 and S5), it is observed that both values are low, except in well-conserved groups such as Flagellicaudata, Diplodocidae, and Dicraeosauridae, where these are greater than 60% (Additional file: Figure S4). Support values do not increase or vary considerably when unstable taxa are excluded prior to the analysis (Additional file: Figure S5).

Although the newly recovered clade has clear synapomorphies, it is poorly supported by the Bremer, Jackknife, and Bootstrap analyses. This, coupled with the instability shown by *Malarguesaurus* and *Tehuelchesaurus* in previous phylogenetic analyses [3, 37, 74, 78], leads us to believe that it is not appropriate to name this clade at this stage and we would not do so unless future studies incorporating a larger number of taxa can strengthen its recovery. In summary, the results of this phylogenetic analysis shed light on the fragmentary material from the Portezuelo Formation. Thanks to this, it was possible to identify and support the assignment of these new sauropod materials to the base of the clade Somphospondyli. This tool also allowed us to increase our knowledge of the diversity of sauropod fauna in this formation.

## Discussion

In this work we present sauropod fossil material from two localities in the Portezuelo Formation (upper Turonian–lower Coniacian) that is composed primarily of caudal vertebrae. Despite being represented by isolated caudal vertebrae, specimens MCF-PVPH 916 and 917 possess an anatomical overlap that includes the vertebrae of the midsection of the tail. These elements do not show differences that could indicate that they belong to different taxa: the middle caudal vertebrae are amphicoelous, lacking excavations or ridges on the lateral or ventral surfaces; both specimens show a notch on the dorsal margin of the posterior articular surface of the centrum; and both have the neural arch located in the anterior half of the centrum. The phylogenetic analysis also recovered both MCF-PVPH 916 and 917 as closely related taxa (Fig. 5), but only a small number of characters were scored in the matrix: 28 (7%) of characters for specimen MCF-PVPH 916 and 53 (12%) for specimen MCF-PVPH 917. Despite not showing significant differences in their homologous elements, the incompleteness of both specimens MCF-PVPH916 and 917, and the distance that separates the localities where they were found (around 300 m), do not allow us to confirm that they belong to the same taxon, but we cannot rule out that possibility either.

Anatomical features of MCF-PVPH 916 and 917 and our phylogenetic analysis allow us to identify both specimens as non-titanosaurian somphospondylans. The assignment to Titanosauriformes is based on the presence of the following synapomorphies: anterior caudal vertebrae with posteriorly extended transverse processes reaching the posterior articular surface of the centrum; neural spine of anterior caudal vertebra with a lateromedial width of ∼ 50% of its anteroposterior length (lognkosaurians show a laterally expanded neural spine in anterior caudal vertebrae); anterior and middle caudal vertebrae with the neural arch restricted to the anterior half of the centrum; middle caudal vertebrae with a circular centrum in cross-section (Lognkosaurians show trapezoidal middle caudal vertebrae in cross-section). Additionally, the axial elements from the Sierra del Portezuelo area show the following non- titanosaurian characters: anterior caudal vertebrae without fossae or pits on the lateral surfaces of the centrum; absence of a bulge on the ventral surface of the transverse processes of anterior caudal vertebrae (plesiomorphic character among non-titanosauriform sauropods acquired in Saltasauroidea). Considering Titanosauria and more nested groups, the Sierra del Portezuelo specimens have the following pleisiomorphic conditions: amphicoelous middle–posterior caudal vertebrae (strongly procoelous caudal centra are diagnostic of titanosaurians); amphicoelous posterior caudal vertebrae (different to procoelous posterior centra widely represented in derived titanosaurians).

### Comparisons with sauropods from the Portezuelo formation

As mentioned above, the anatomical information of both specimens comes mainly from caudal vertebrae, with limited appendicular information from MCF-PVPH 917. In this section we compare the new specimens MCF-PVPH 916 and 917 from the Sierra del Portezuelo area with existing sauropod records from the continental sediments of the upper Turonian–lower Coniacian Portezuelo Formation. *Malarguesaurus florenciae* [30] and *Futalognkosaurus dukei* [31] are the only named sauropodan species from the formation with other sauropod material being identified to coarser taxonomic levels: isolated caudal vertebrae assigned to Titanosauriformes [54] and axial and appendicular elements from at least two individuals assigned to Titanosauria [41].

**Fig. 6:**
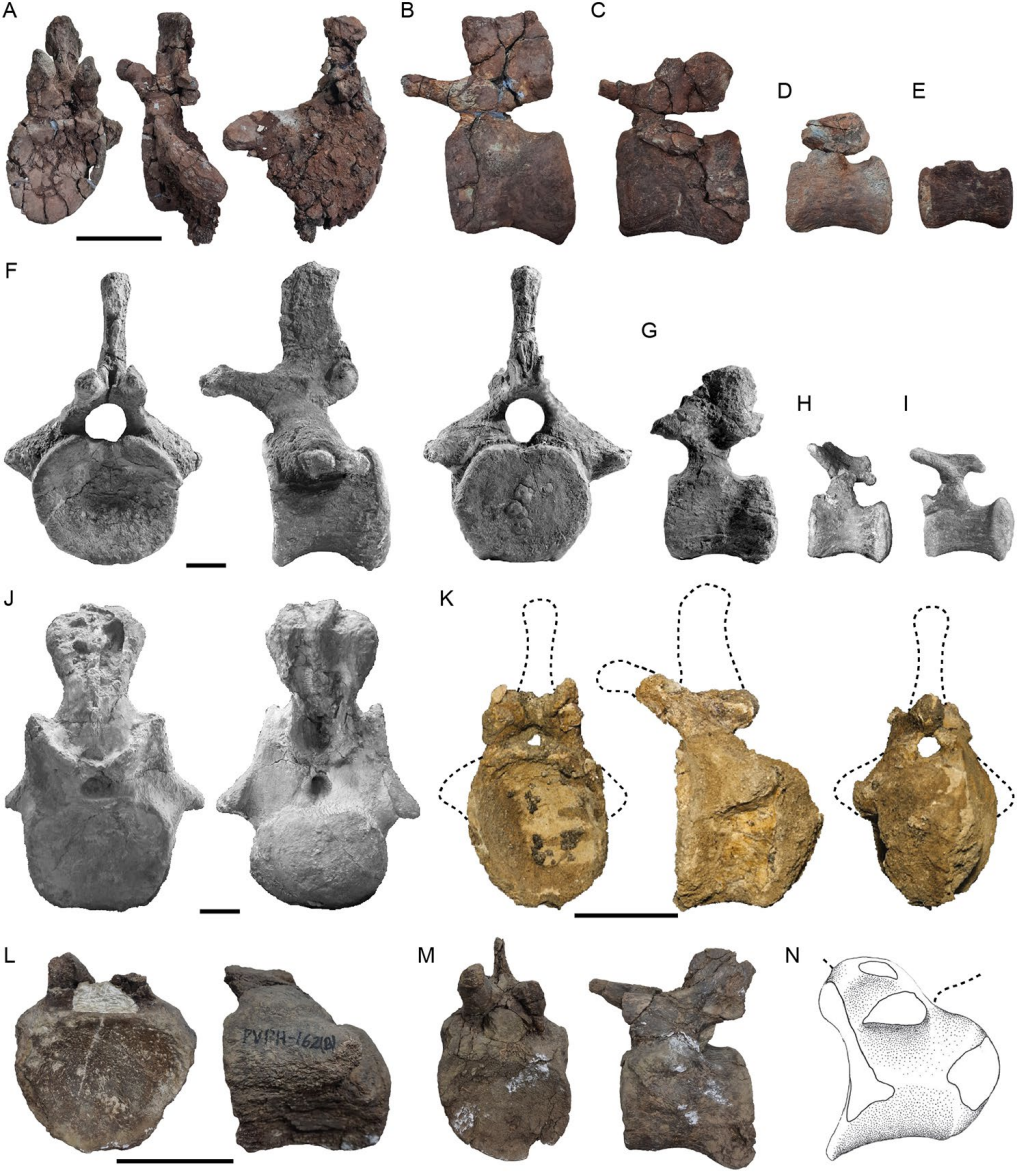
Caudal vertebrae of sauropods from the Portezuelo Formation. Anterior caudal vertebra MCF-PVPH 917/1 in anterior, left lateral, and posterior views (**A**); anterior–middle caudal vertebra MCF-PVPH 917/2 in left lateral (inverted) view (**B**); middle caudal vertebra MCF-PVPH 917/3 in left lateral view (**C**); posterior caudal vertebra MCF-PVPH 916/4 in left lateral (inverted) view (**D**); posterior caudal vertebra MCF-PVPH 916/5 in left lateral view (**E**); anterior caudal vertebra of *Malarguesaurus florenciae* (IANIGLA-PV 110/1; modified from [30]) in anterior, left lateral, and posterior views (**F**); middle caudal vertebra of *Malarguesaurus* (IANIGLA-PV 110/3; modified from [30]) in left lateral view (**G**); posterior caudal vertebra of *Malarguesaurus florenciae* (IANIGLA-PV 110/5; modified from [30]) in left lateral view (**H**); posterior caudal vertebra of *Malarguesaurus florenciae* (IANIGLA-PV 110/6; modified from [30]) in left lateral view (**I**); first caudal vertebra of *Futalognkosaurus dukei* (MUCPv 323; modified from [31]) in anterior and posterior views (**J**); anterior caudal vertebra from Los Bastos locality (MMS-PV 09; modified from [41]) in anterior, left lateral, and posterior views (**K**); anterior caudal vertebra of MCF-PVPH 162 in anterior and left lateral views (**L**); middle caudal vertebra of MCF-PVPH 162 in anterior and left lateral views (**M**); middle caudal vertebra of MCF-PVPH 163 (modified from [54] in left lateral (inverted) view (**N**). Scale bars of 10 cm

*Malarguesaurus florenciae* is a large titanosauriform represented by axial elements (anterior, middle and posterior caudal vertebrae, chevrons, and dorsal ribs), and a few appendicular bones (a fragment of humerus and an incomplete femur; [30]). Our phylogenetic analysis recovers *Malarguesaurus* as a non-titanosaurian somphospondylan within a monophyletic clade comprising *Tastavinsaurus*, *Tehuelchesaurus* as well as MCF-PVPH 916 and 917 (Fig. 5). However, recent phylogenetic analyses show that the position of *Malarguesaurus* is unstable (e.g., [37, 74, 79]). Similar to MCF-PVPH 917, the anterior caudal vertebra of *Malarguesaurus* have the following features (Fig. 6A, F): probable presence of procoelous–opisthoplatyan anterior caudal centra; posteriorly extended transverse processes reaching the posterior face of the centrum; neural arch occupying the anterior half of the centrum and lacking pneumaticity; absence of a hyposphene ridge; absence of the SPDL. As for the differences, some may be due to serial variation along the tail: the neural spine of the anterior caudal vertebra of *Malarguesaurus* is more transversely compressed and is higher than that of MCF-PVPH 917 (the ratio of the height of the neural spine above the prezygapophyseal process and centrum height is 1.15 in *Malarguesaurus* but only 0.7 in MCF-PVPH 917); the height of the pedicel below the prezygapophyseal process in the middle caudal vertebrae is greater in *Malarguesaurus* (Fig. 6B-C, G). A notable difference occurs in the dorsal margin of the neural spine of the anterior caudal vertebra: while MCF-PVPH 917 has a flat margin, *Malarguesaurus* has a neural spine with a convex anterior and concave posterior dorsal margins (Fig. 6A, F), which is a probable autapomorphy of this taxon [30]. However, given that there are no more anterior caudal vertebrae preserved in MCF-PVPH 917 and *Malarguesaurus*, we cannot assume that this difference is not due to serial variation. The centra of the middle caudal vertebrae of *Malarguesaurus* are wider than high, and have a circular anterior face and a subquadrangular posterior one [30], while the middle caudal vertebrae of the specimen MCF-PVPH 917 are slightly taller than they are wide, although this difference appears to be taphonomic, due to the general mediolateral crushing of the vertebrae. Due to these differences between the specimen MCF-PVPH 917 and *Malarguesaurus*, which could be related with the position in the series or the taphonomy, we cannot rule out the possibility that they are the same taxon.

As for specimen MCF-PVPH 916, the most notable difference with *Malarguesaurus* is found in the type of articulation of the posterior caudal vertebrae. While specimen MCF-PVPH 916 has amphicoelous middle caudal vertebrae associated with slightly amphicoelous posterior caudal vertebrae, *Malarguesaurus* has procoelous-opisthoplatyan middle caudal vertebrae associated to slightly to strongly procoelous posterior caudals (Fig. 6D-E, H-I), which is considered an autapomorphic character of this taxon [30]. Although the articulations of the caudal vertebrae are usually variable along the tail of a same individual in somphospondylans and early branching titanosaurians (e.g., *Tastavinsaurus*, *Andesaurus*, and *Mendozasaurus*; [49, 60, 66]), both middle and posterior caudal vertebrae of MCF-PVPH 916 are slightly amphicoelous, and in the latter they do not have a convex posterior articular surface as in overlapping vertebrae of *Malarguesaurus*. The possibility that these differences may be intraspecific is difficult to test due to the limited material available from the specimens studied here, and taxa such as *Malarguesaurus* with incomplete caudal series. Intragenus or intraspecific variations have been studied in taxa represented by numerous individuals, such as the diplodocids *Apatosaurus* and *Diplodocus*, or the macronarian *Camarasaurus*, but their intrageneric relationships are still not clear [13]. However, with the information we have available, we can note that specimen MCF-PVPH 916 has a different combination of features than *Malarguesaurus* in terms of the articulation of its caudal series, so it is likely that the former could represent a different taxon than *Malarguesaurus*. *Futalognkosaurus dukei* is a giant lognkosaurian, which is preserved as a complete neck, dorsal vertebrae, dorsal ribs, the first caudal vertebra, and appendicular elements [26, 80]. As the only preserved caudal element of this taxon is the first caudal vertebra, there is no overlap of elements with MCF-PVPH 916 and 917, so a detailed comparison is not possible. However, the anterior caudal vertebra of *Futalognkosaurus* possesses a combination of characters typical of anterior vertebrae of derived titanosaurians that are not observed in MCF-PVPH 917 (Fig. 6A, J), such as transverse processes with a high lateral margin that do not taper distally, neural spines that are lateromedially expanded ∼ 1.5 times their anteroposterior length, and conspicuous laminae such as the SPDL and ventral SPRL.

As mentioned previously, sauropod materials also emerged from Los Bastos locality (Neuquén Province, Argentina) of the Portezuelo Formation, which include an isolated tooth, an anterior caudal centrum, and partially associated axial and appendicular elements belonging to at least two individuals (i.e., anterior caudal vertebrae, a right ulna, a right radius, a right metacarpal IV, a left fibula, and a right femur), which were assigned to indeterminate colossosaurian titanosaurians [41]. The differences between these materials and MCF-PVPH 917 are restricted to the axial skeleton. While the anterior caudal vertebrae of the Los Bastos specimens have small vascular foramina in their lateroventral surfaces, these are absent in MCF-PVPH 917. The anterodorsal orientation of the prezygapophyseal processes of the anterior caudal vertebra from the Los Bastos locality (MMS-PV 09) is similar to that of MCF-PVPH 917 (Fig. A-K). However, the anterior caudal vertebra of the Los Bastos locality has a markedly procoelous centrum with a deeper concave anterior surface than MCF-PVPH 917 (Fig. 6A, K).

Finally, the remaining sauropod material to be compared with MCF-PVPH 916 and 917 also come from the Sierra del Portezuelo area, and consist of indeterminate titanosaurian axial elements (MCF-PVPH 162 and 163; [54]). MCF-PVPH 162 comprises one anterior and one middle caudal vertebra, whereas MCF-PVPH 163 comprises only a middle caudal vertebra. The anterior caudal vertebra of MCF-PVPH 162 is markedly procoelous, a condition that is not observed in the anteriormost preserved caudal vertebrae of MCF-PVPH 917 which is more likely to be procoelous–opisthoplatyan, since its anterior articular surface is slightly concave, and its posterior articular surface (although damaged) could not have originally had a prominent convexity (Fig. 6-B, L). Similar to the middle caudal vertebrae of MCF-PVPH 917 and 916, the middle caudal vertebra of MCF-PVPH 162 is amphicoelous with its anterior surface more concave than the posterior one (Fig. 6C, M), although this lacks the notch on the dorsal margin of the posterior articular surface present in these specimens. In MCF-PVPH 917, the shape and orientation of the neural spine, as well as its posterior orientation and distal extent that does not exceed the posterior articular face of the centrum, are features also found in MCF-PVPH 162. However, the neural spine in MCF-PVPH 162 is more posteriorly placed on the centrum, with its anterior margin on its posterior half ([54]: Fig. 3D), whereas in the middle caudal MCF-PVPH 917/3 the anterior margin is located on the anterior third of the centrum (Fig. 6C, M). Furthermore, the neural arch of the middle caudal vertebra of MCF-PVPH 162 occupies a greater dorsal surface over the centrum than that of specimen MCF-PVPH 917 (Fig. 6C, M). The articular surface of the postzygapophyses is markedly concave in MCF-PVPH 162, while in MCF-PVPH 917 they are flat. On the other hand, although specimen MCF-PVPH 163 is represented by a very incomplete middle caudal vertebra, its procoelous articulation makes it different from specimen MCF-PVPH 162 and 916 and 917 (Fig. 6C, N). In this way, specimens MCF-PVPH 162 and 163 presented by [54] not only show differences between them that could consider them to be different taxa, but they are also different from MCF-PVPH 916 and 917.

### Implications for Upper Cretaceous sauropod diversity in Patagonia

The fossil record of sauropods from the Turonian–Coniacian of Patagonia comes mostly from the Neuquén Basin (lower Cenomanian–middle Campanian; [23]) and the San Jorge Basin [81, 82]. From the late Cenomanian to early Turonian, we have the Huincul Formation [23], from which is known the rebbachisaurids *Limaysaurus tessonei* [71], *Cathartesaura anaerobica* [83] and *Sidersaura marae* [11], and the titanosaurians *Argentinosaurus huinculensis* [84], *Choconsaurus baileywillisi* [85], *Chucarosaurus diripienda* [86] and *Bustingorrytitan shiva* [77]. Furthermore, for this same period, the titanosaurians *Epachthosaurus sciuttoi* [87], and *Drusilasaura deseadensis* [88], and the rebbachisaurid *Katepensaurus goicoecheai* [89], are known from the Bajo Barreal Formation which pertains to the Chubut Group in the San Jorge Basin [81, 90]. Overlying this formation is the Cerro Lisandro Formation, whose age is estimated as middle–upper Turonian [23], and from which arose the titanosaurian *Quetecsaurus rusconii* [91]. From strata assigned to the upper Turonian– lower Coniacian of the Portezuelo Formation [23], the somphospondylan *Malarguesaurus*, and the longkosaurian *Futalognkosaurus* were formally named. By the Coniacian, the fossil record is practically only represented by titanosaurians. Within Titanosauria, the clades Colossosauria and Lognkosauria can be identified, although early branched titanosaurians like *Kaijutitan maui* [92] (from the Sierra Barrosa Formation; middle– late Coniacian, [23] and titanosaurians with uncertain affiliation like *Elaltitan lilloi* (from the Lago Colhué Huapi Formation; [93]) are also known. Also, the colossosaurian *Mendozasaurus neguyelap* [94] emerged from the Sierra Barrosa Formation. Finally, from the Plottier Formation (late Coniacian–early Santonian; [23]), emerge the colossosaurians *Petrobrasaurus puestohernandezi* [95], *Notocolossus gonzalezparejasi* [96], and *Muyelensaurus pecheni* [26], the latter two being the specifier taxa for the clades Lognkosauria and Rinconsauria respectively.

In this context, the specimens studied here expand the fossil record of the Portezuelo Formation. Although one of these specimens (MCF-PVPH 916) is different from both the formally named taxa of this formation (i.e., *Malarguesaurus* and *Futalognkosaurus*) and from the other specimens presented by other authors [41, 54], we cannot assume that we are facing a new species due to its incompleteness. However, we are seeing that the diversity of sauropods in the Portezuelo Formation is greater than what was known until now, being considerable compared to the other formations of the Turonian–Coniacian of Patagonia.

## Conclusions

Despite there being numerous sauropod fossils from the upper Turonian–lower Coniacian Portezuelo Formation, only two species have been formally named: the non-titanosaurian somphospondylan *Malarguesaurus florenciae* [30] and the lognkosaurian *Futalognkosaurus dukei* [31]. Further sauropod materials correspond to mostly disarticulated elements of incomplete titanosauriform specimens, mainly composed of caudal axial elements [41, 54]. MCF-PVPH 916 and 917 presented here come from the Sierra del Portezuelo area, and are composed of caudal vertebrae without clear articular association from at least two different specimens. In other independent phylogenetic analyses [37, 41, 74], materials from the Portezuelo Formation (e.g., *Malarguesaurus* and material from the Los Bastos locality), have been shown to be phylogenetically unstable due to the incompleteness of the specimens. The phylogenetic analysis presented here shows that the new specimens from the Sierra del Portezuelo area (MCF-PVPH 916 and 917) form, together with *Malarguesaurus, Tastavinsaurus* and *Tehuelchesaurus* an early-branching clade within Somphospondyli.

The specimens MCF-PVPH 916 and 917 described here lack differences in their caudal vertebrae from homologous positions that could indicate that they belong to different taxa, but the distance that separates the sites where they were found, and the incompleteness of both specimens do not allow us to accept this hypothesis either. On the other hand, there are differences in the caudal vertebrae of these specimens with those of other specimens described from the same formation, such as the lognkosaurian *Futalognkosaurus* and the specimens referred to as titanosaurians by other authors (i.e., [41, 54]). MCF-PVPH 917 shows differences with the somphospondylian *Malarguesaurus*, although these could be due to variations in the caudal series along the tail, or due to taphonomic causes, so we cannot rule out that this specimen represents another individual of this taxon. On the other hand, specimen MCF-PVPH 916 has amphicoelous middle and posterior caudal vertebrae, which contrasts with the caudal series of *Malarguesaurus*, where the posterior caudal vertebrae show a convexity on the posterior articulating surface. In this sense, we can consider the presence of at least one taxon different from what is known for the Portezuelo Formation, which would expand the faunal record for the formation. However, due to the incompleteness of the known specimens for this formation, some of which are represented by few elements, future fieldwork will be required to obtain more complete specimens to better justify the presence of a new taxon. These specimens expand our knowledge about the Turonian–Coniacian ecosystems of South America, confirming the success of these animals in the Upper Cretaceous faunae of Gondwana.

### Electronic supplementary material

Below is the link to the electronic supplementary material.

Supplementary Material 1

## Data Availability

The materials studied here are housed in the palaeontological collections of the Museo Carmen Funes (Plaza Huincul, Neuquén Province, Argentina). All data arising from this work are provided in the manuscript and the additional file.
