# Supplementary material for: Unraveling sauropod diversity in the Portezuelo Formation of Patagonia through a comprehensive analysis of new and existing material
Source: BMC Ecol Evol. 2024 Jul 9;24:96. doi: 10.1186/s12862-024-02280-9 (PMC11234639; doi:10.1186/s12862-024-02280-9)
Supplement: Supplementary file 1 — Supplementary Material 1 [file 12862_2024_2280_MOESM1_ESM.docx]

Supplementary data for

Unraveling sauropod diversity in the Portezuelo Formation of Patagonia through a comprehensive analysis of new and existing material

K.L. Gomez^1,2*^, A. Pérez-Moreno^1,3^, J.G. Meso^1,2^, Flavio Bellardini^1,2^, M. A. Baiano^1,4,5^, D. Pol^1,6^, A. Garrido^7^, J. Kaluza^8^, L. Muci^9^& M. Pittman^4*^

^1^Consejo Nacional de Investigaciones Científicas y Técnicas (CONICET), Argentina.

^2^Instituto de Investigación en Paleobiología y Geología (IIPG); Universidad Nacional de Río Negro (UNRN) - Consejo Nacional de Investigaciones Científicas y Técnicas (CONICET), General Roca 8332, Río Negro Province, Argentina. [kevinn.lgomez@gmail.com](mailto:kevinn.lgomez@gmail.com), [mbaiano@unrn.edu.ar](mailto:mbaiano@unrn.edu.ar)

^3^División Paleontología de Vertebrados, Museo de La Plata (Anexo), Calle 122 y 60, La Plata (B1900WA), Buenos Aires Province, Argentina. [aperezmoreno7@gmail.com](mailto:aperezmoreno7@gmail.com)

^4^School of Life Sciences, The Chinese University of Hong Kong, Shatin, Hong Kong SAR, China. [mpittman@cuhk.edu.hk](mailto:mpittman@cuhk.edu.hk), [mattiaantoniobaiano@cuhk.edu.hk](mailto:mattiaantoniobaiano@cuhk.edu.hk)

^5^Área Laboratorio e Investigación, Museo Municipal ‘Ernesto Bachmann’, Dr Natali S/N, 8311 Villa El Chocon, Neuquén Province, Argentina.

^6^Museo Paleontológico Egidio Feruglio, Trelew, Chubut Province, Argentina. [cacopol@gmail.com](mailto:cacopol@gmail.com)

^7^Museo Provincial de Ciencias Naturales ‘Prof. Dr. Juan A. Olsacher’. Dirección Provincial de Minería, Zapala, Neuquén Province, Argentina. [albertogarrido@gmail.com](mailto:albertogarrido@gmail.com)

^8^Fundación de Historia Natural Félix de Azara, Universidad Maimónides. Hidalgo 775, C1405, Ciudad autónoma de Buenos Aires, Buenos Aires Province, Argentina. [yojonatan@hotmail.com](mailto:yojonatan@hotmail.com)

^9^Universidad Nacional de Río Negro, Sede Alto Valle/Valle Medio. Estados Unidos 750, R8332 General Roca, Río Negro Province, Argentina. [luciana.muci97@gmail.com](mailto:luciana.muci97@gmail.com)

*Corresponding author

Contents

Figure S1 3

Figure S2 4

Figure S3 5

Figure S4 6

Figure S5 7

List of Characters 8-30

Synapomorphy List 31-46

Data Matrix 47-63


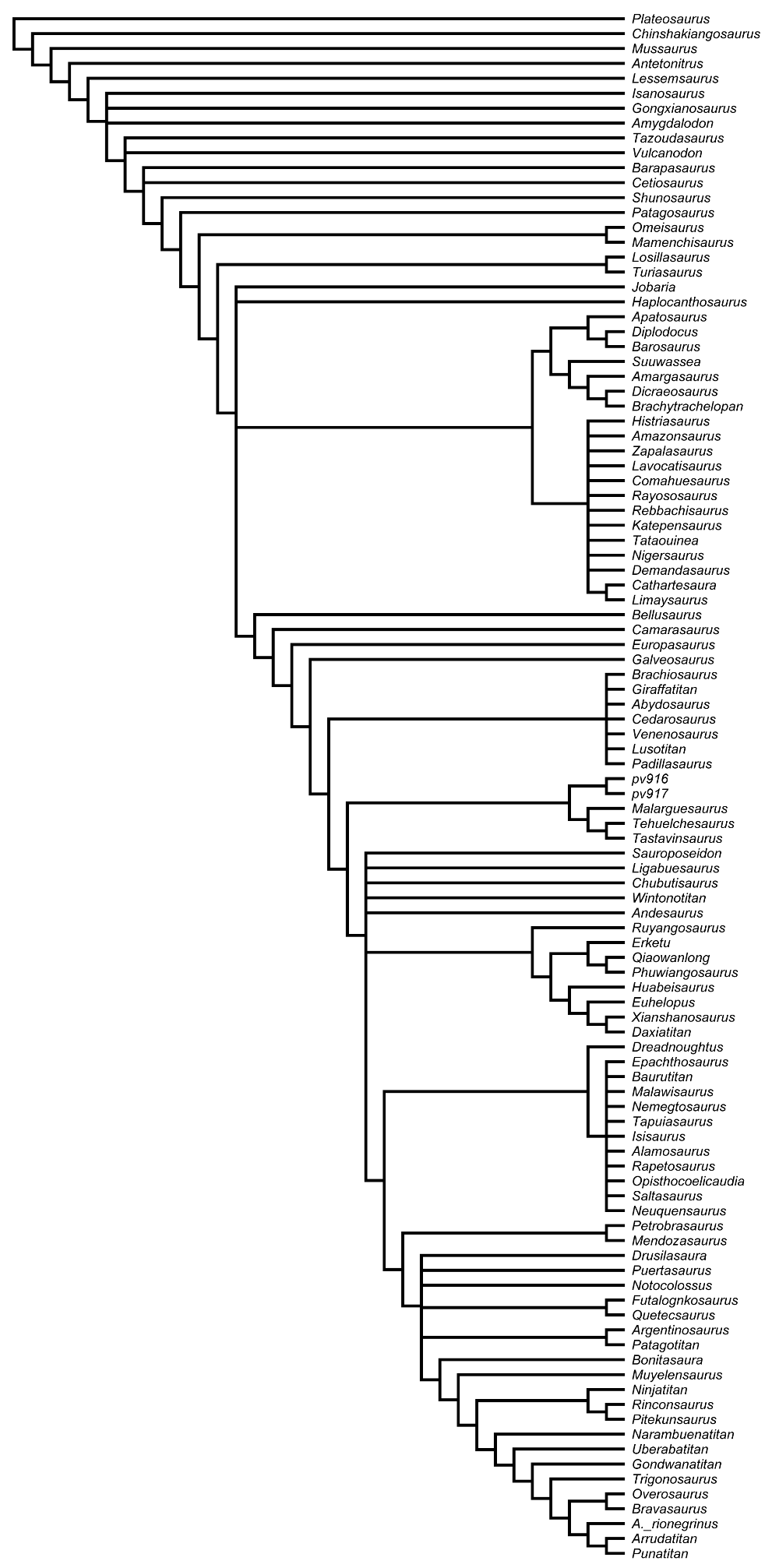


Figure S1. Strict consensus of more than 400,000 of the most parsimonious trees (MPT of 1602 steps) with all 104 taxa in the matrix (Consistency Index = 0.33; Retention Index = 0.71).


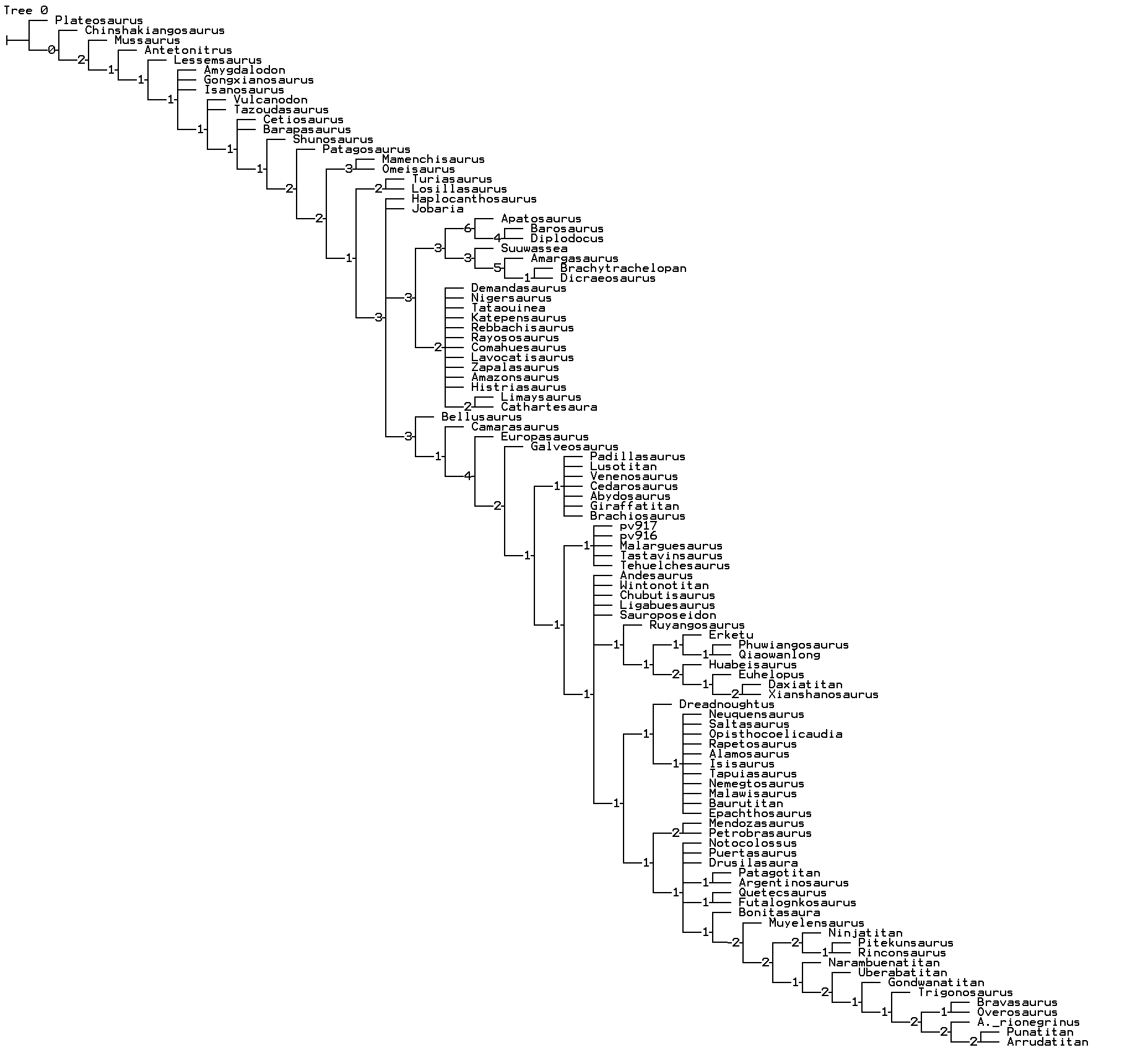


Figure S2. Bremer support values calculated with all 104 taxa and all most parsimonious trees. The search for suboptimal trees was performed by saving up to 1000 trees up to 1 step longer, increasing the score by 1 at a time. For groups not lost in suboptimal trees, the search with restrictions was repeated 3 times and the minimum score was used. The image shows the support values for each node.


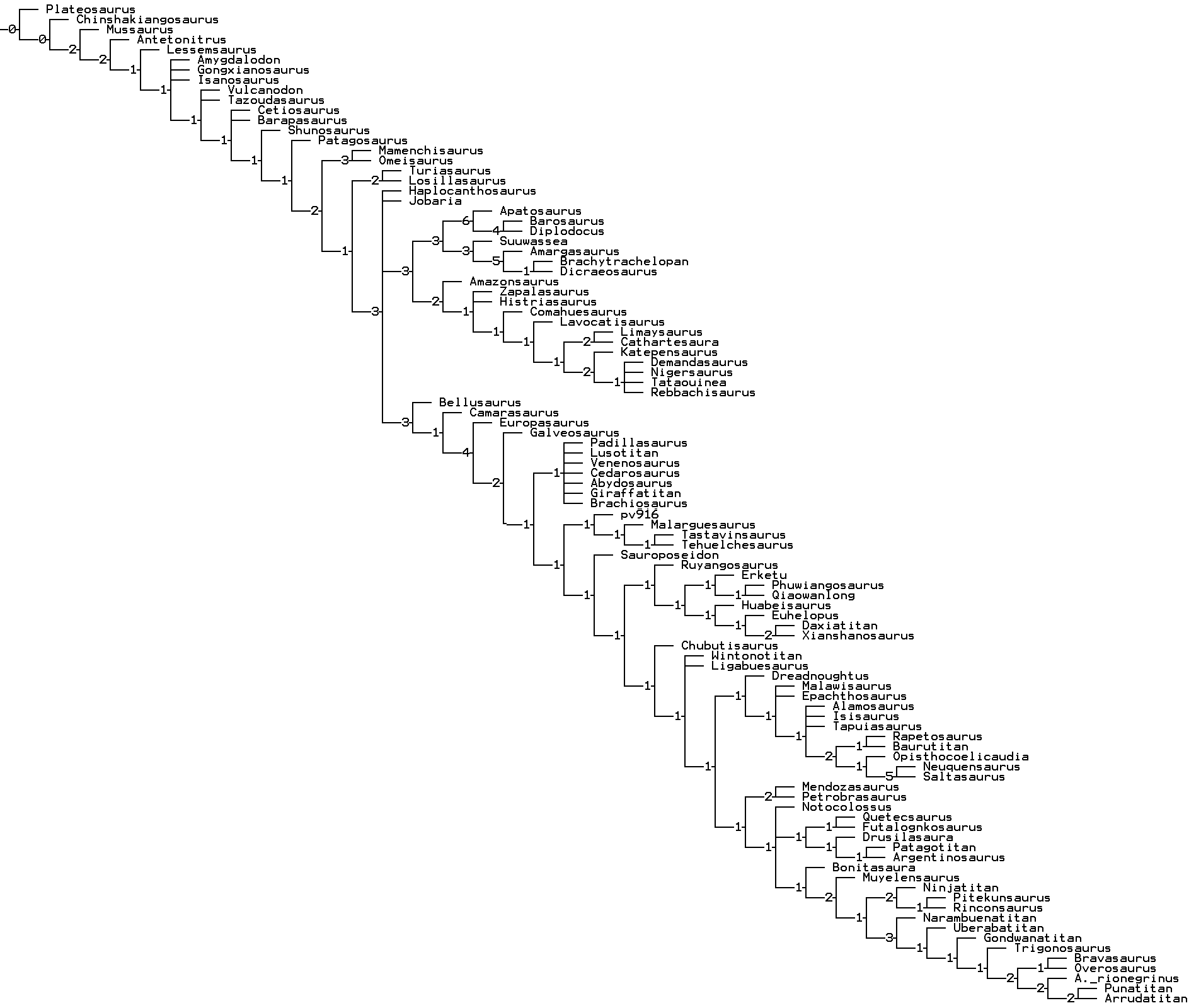


Figure S3. Bremer support values calculated using the most parsimonious trees but without the most unstable taxa. The settings for this analysis were the same as for the Bremer support values calculated with all 104 taxa (Fig. S2). The image shows the support values for each node.


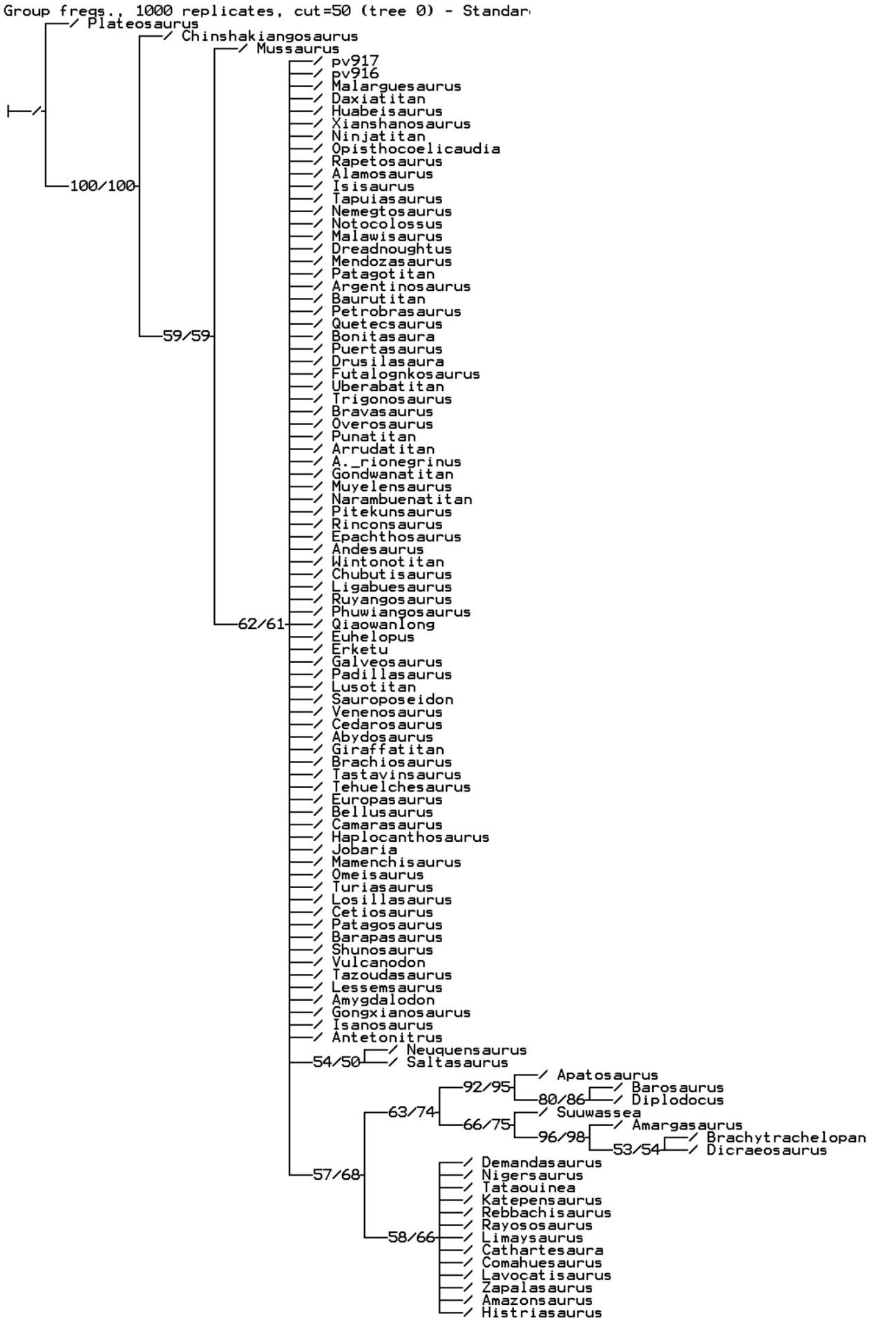


Figure S4. Bootstrap and Jackknife support values exceeding 50% with absolute frequency, calculated with standard substitution and using 1000 replicates.


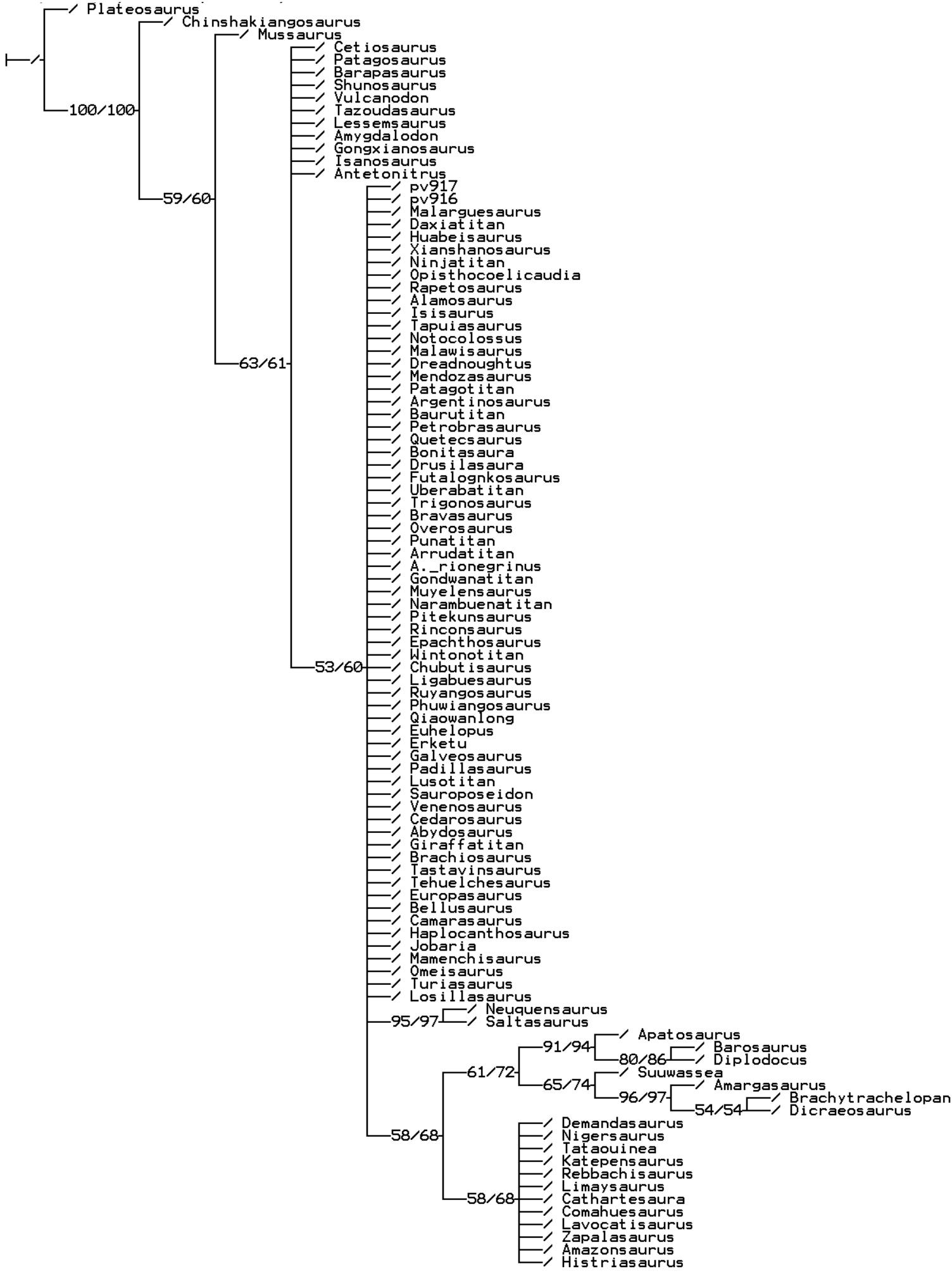
Figure S5. Bootstrap and Jackknife support values exceeding 50% with absolute frequency, calculated with standard substitution and using 1000 replicates. Both supports were calculated using the most parsimonious trees but without the most unstable taxa.

List of characters (Perez-Moreno et al., 2023)

1. Posterolateral processes of premaxilla and lateral processes of maxilla, shape: without midline contact (0); with midline contact forming marked narial depression, subnarial foramen not visible laterally (1). (Wilson, 2002:character 1).
2. Premaxillary anterior margin shape: without step (0); with marked step but short step (1); with marked and long step (2) (modified from Wilson, 2002:character 2).
3. Premaxilla, ascending process shape in lateral view: convex (0); concave, with a large dorsal projection (1); sub-rectilinear and directed posterodorsally (2). (Whitlock, 2011:character 3)
4. Premaxilla, external surface: without anteroventrally orientated vascular grooves originating from an opening in the maxillary contact (0); vascular grooves present (1). (Whitlock, 2011:character 2)
5. Premaxilla-maxilla suture, shape: planar (0); twisted along its length, giving the contact a sinuous appearance in lateral view (1). (D’Emic, 2012:character 2)
6. Premaxilla, small finger-like, vertically oriented premaxillary process near anteromedial corner of external naris: (0) absent; (1) present. (D’Emic, 2012:character 3)
7. Maxillary border of external naris, length: short, making up much less than one-fourth narial perimeter (0); long, making up more than one third narial perimeter (1). (Wilson, 2002:character 3).
8. Maxilla, foramen anterior to the preantorbital fenestra: absent (0); present (1). (Zaher et al., 2011:character 244).
9. Preanteorbital fenestra: absent (0); present, being wide and laterally opened (1). (Modifiedfrom Wilson, 2002:character 4).
10. Subnarial foramen and anterior maxillary foramen, position: well distanced from one another (0); separated by narrow bony isthmus (1). (Wilson, 2002:character 5)
11. Antorbital fenestra: much shorter than orbital maximum diameter, less than 85% of orbit (0); subequal to orbital maximum diameter, greater than 85% orbit (1). (Modifiedfrom Wilson, 2002:character 6 following to Whitlock, 2011:character 13)
12. Antorbital fenestra, shape of dorsal margin: straight or convex (0); concave (1). (Whitlock, 2011:character 14).
13. Antorbital fossa: present (0); absent (1). (Wilson, 2002:character 7)
14. External nares position: terminal (0); retracted to level of orbit (1); retracted to a position between orbits (2). (Wilson, 2002:character 8)
15. External nares, maximum diameter: shorter (0); or longer than orbital maximum diameter (1). (Wilson, 2002:character 9)
16. Orbital ventral margin, anteroposterior length: broad, with subcircular orbital margin (0); reduced, with acute orbital margin (1). (Wilson, 2002:character 10)
17. Lacrimal, anterior process: present (0); absent (1). (Wilson, 2002:character 11)
18. Lacrimal, anteriorly projecting vertical plate of bone: absent (0); present (1). (D’Emic, 2012: character 4)
19. Jugal contribution to the ventral border of the skull: present and long (0); absent or very reduced (1). (Carballido et al., 2012:character 16).
20. Quadratojugal-Maxilla contact: absent or small (0); broad (1). (Whitlock, 2011:character 10).
21. Jugal-ectopterygoid contact: present (0); absent (1). (Wilson, 2002:character 12)
22. Jugal, contribution to antorbital fenestra: absent (0); present, but very reduced (1); present and large, bordering approximately one-third its perimeter (2). (Modifiedfrom Wilson, 2002:character 13).
23. Quadratojugal, position of anterior terminus: posterior to orbit (0); between the orbit (1); anteriorly surpassing the orbit (2). (modified by Canudo et al.; 2019 in the base of Whitlock, 2011:character 30).
24. Quadratojugal, anterior process length: short, anterior process shorter than dorsal process (0); long, anterior process more than twice as long as dorsal process (1). (Wilson, 2002:character 32)
25. Quadratojugal, angle between anterior and dorsal processes: less than or equal to 90°, so that the quadrate shaft is directed dorsally (0); greater than 90°, approaching 130°, so that the quadrate shaft slants posterodorsally (1). (Whitlock, 2011:character 31).
26. Ventral edge of anterior surface of the quadratojugal: straight, not expanded ventrally (0); slightly expanded ventrally, forming a small bulge, which height is less than twice the ramus height (1); well expanded ventrally, forming a notorious bulge, which height is twice or more the minimum height of the ramus (2). (Modifiedfrom Upchurch et al., 2004:character 26)
27. Squamosal contribution to the supratemporal fenestra: present, the squamosal is well visible in dorsal view (0); reduced or absent (1). (Curry Rogers, 2005:character 37).
28. Squamosal-quadratojugal contact: present (0); absent (1). (Wilson, 2002:character 31)
29. Squamosal, posteroventral margin: smooth (0); "with prominent, ventrally directed "prong" (1). (Whitlock, 2011:character 37).
30. Prefrontal posterior process size: small, not projecting far posterior of frontal-nasal suture (0); elongate, approaching parietal (1). (Wilson, 2002:character 14)
31. Prefrontal, posterior process shape: flat (0); hooked (1). (Wilson, 2002:character 15)
32. Prefrontal, anterior process: absent (0); present (1). (Curry Rogers, 2005:character 30)
33. Prefrontal-Frontal contact width: large, equal or longer than the anteroposterior length of the prefrontal (0); narrow, less than half the anteroposterior length of the prefrontal (1). (Zaher et al., 2011:character 239).
34. Postorbital, ventral process shape: transversely narrow (0); broader transversely than anteroposteriorly (1). (Wilson, 2002:character 16).
35. Postorbital, posterior process: present (0); absent (1). (Wilson, 2002:character 17).
36. Postorbital, posterior margin articulating with the squamosal: with tapering posterior process (0); with a deep posterior process (1). (Zaher et al., 2011:character 245).
37. Frontal contribution to supratemporal fossa: present (0); absent (1). (Wilson, 2002:character 18)
38. Frontals, midline contact (symphysis): sutured (0); or fused in adult individuals (1). (Wilson, 2002:character 19)
39. Frontal, anteroposterior length: approximately twice (0); or less than minimum transverse breadth (1). (Wilson, 2002:character 20)
40. Frontal-nasal suture, shape: flat or slightly bowed anteriorly (0); V-shaped, pointing posteriorly (1). (Whitlock, 2011:character 21)
41. Frontals, dorsal surface: without paired grooves facing anterodorsally (0); grooves present, extend on to nasal (1). (Whitlock, 2011:character 22)
42. Frontal, contribution to dorsal margin of orbit: contribution to dorsal margin of orbit: less than 1.5 times the contribution of prefrontal (0); at least 1.5 times the contribution of prefrontal (1). (Whitlock, 2011:character 23)
43. Parietal occipital process, dorsoventral height: short, less than the diameter of the foramen magnum (0); deep, nearly twice the diameter of the foramen magnum (1). (Wilson, 2002:character21)
44. Parietal, contribution to post-temporal fenestra: present (0); absent (1). (Wilson, 2002:character 22)
45. Parietal, distance separating supratemporal fenestrae: less than the long axis of supratemporal fenestra, 0.8 or less (0); almost the same than the long axis of supratemporal fenestra 0.8-1.2 (1); much larger than the long axis of supratemporal fenestra more than 1.2 (2). (Modifiedfrom Wilson, 2002: character 24).
46. Postparietal foramen: absent (0); present (1). (Wilson, 2002:character 23)
47. Paroccipital process distal terminus: straight, slightly expanded surface (0); rounded, tongue-like process (1). (Whitlock, 2011:character 42)
48. Supratemporal fenestra: present (0); absent (1). (Wilson, 2002:character 25)
49. Supratemporal fenestra, long axis orientation: anteroposterior (0); transverse (1). (Wilson, 2002:character26)
50. Supratemporal fenestra, maximum diameter: much longer than (0); or subequal to that of foramen magnum (1). (Wilson, 2002:character 27)
51. Supratemporal region, anteroposterior length: temporal bar longer (0); or shorter anteroposteriorly than transversely (1). (Wilson, 2002:character 28)
52. Supratemporal fossa, lateral exposure: not visible laterally, obscured by temporal bar (0); visible laterally, temporal bar shifted ventrally (1). (Wilson, 2002:character 29)
53. Supraoccipital, sagittal nuchal crest: broad, weakly developed (0); narrow, sharp and distinct (1). (Whitlock, 2011:character 45). 46
54. Laterotemporal fenestra, anterior extension: posterior to orbit (0); ventral to orbit (1). (Wilson, 2002:character 30)
55. Quadrate fossa: absent (0); present (1). (Wilson, 2002:character 33)
56. Quadrate fossa, depth: shallow (0); deeply invaginated (1). (Wilson, 2002:character 34)
57. Quadrate fossa, orientation: posterior (0); posterolateral (1). (Wilson, 2002:character 35)
58. Quadrate, articular surface shape: quadrangular in ventral view, oriented transversely (0); roughly triangular in shape or thin, crescent-shaped surface with anteriorly directed medial process (1). (Modifiedbasedon Mannion et al., 2011. from Whitlock, 2011:character 32).
59. Quadrate, articular surface shape: quadrangular in ventral view, oriented transversely or roughly triangular in shape (0); thin, crescent-shaped surface with anteriorly directed medial process (1). (Modifiedbasedon Mannion et al., 2011 from Whitlock, 2011:character 32).
60. Palatobasal contact, shape: pterygoid with small facet (0); dorsomedially orientated hook (1); or rocker-like surface for basipterygoid articulation (2). (Wilson, 2002:character 36)
61. Pterygoid, transverse flange (i.e. ectopterygoid process) position: posterior of orbit (0); between orbit and antorbital fenestra (1); anterior to antorbital fenestra (2). (Wilson, 2002:character37)
62. Pterygoid, quadrate flange size: large, palatobasal and quadrate articulations well separated (0); small, palatobasal and quadrate articulations approach (1). (Wilson, 2002:character 38)
63. Pterygoid, palatine ramus shape: straight, at level of dorsal margin of quadrate ramus (0); stepped, raised above level of quadrate ramus (1). (Wilson, 2002:character39)
64. Pterygoid, sutural contact with ectopterygoid: broad, along the medial or lateral surface (0); narrow, restricted to the anterior tip of the ectopterygoid (1). (Zaher et al. 2011:character 240)
65. Palatine, lateral ramus shape: plate-shaped (long maxillary contact) (0); rod-shaped (narrow maxillary contact) (1). (Wilson, 2002:character 40)
66. Epipterygoid: present (0); absent (1). (Wilson, 2002:character 41)
67. Vomer, anterior articulation: maxilla (0); premaxilla (1). (Wilson, 2002:character 42)
68. Supraoccipital, height: twice subequal to (0); or less than height of foramen magnum (1). (Wilson, 2002:character 43)
69. Paroccipital process, ventral non-articular process: absent (0); present (1). (Wilson, 2002:character 44)
70. Crista prootica, size: rudimentary (0); expanded laterally into dorsolateral process (1). (Wilson, 2002:character 45)
71. Basipterygoid processes, length: short, approximately twice (0); or elongate, at least four times basal diameter (1). (Wilson, 2002:character 46)
72. Basipterygoid processes, angle of divergence: approximately 45° (0); less than 30° (1). (Wilson, 2002:character 47) 47
73. Basal tubera, anteroposterior depth: approximately half dorsoventral height (0); sheet-like, 20% dorsoventral height (1). (Wilson, 2002:character 48)
74. Basal tubera, breadth: much broader than (0); or narrower than occipital condyle (1). (Wilson, 2002:character 49)
75. Basal tubera: distinct from basipterygoid (0); reduced to slight swelling on ventral surface of basipterygoid (1). (Whitlock, 2011:character 53)
76. Basal tubera, shape of posterior face: convex (0); slightly concave (1). (Whitlock, 2011:character 54)
77. Basioccipital depression between foramen magnum and basal tubera: absent (0); present (1). (Wilson, 2002:character 50)
78. Basisphenoid/basipterygoid recess: present (0); absent (1). (Wilson, 2002:character 51)
79. Basisphenoid/quadrate contact: absent (0); present (1). (Wilson, 2002)
80. Basisphenoid, sagittal ridge between basipterygoid processes: absent (0); present (1). (Zaher et al., 2011:character 242)
81. Basipterygoid processes, orientation: perpendicular to (0); or angled approximately 45° to skull roof (1). (Wilson, 2002:character 53)
82. Basipterygoid, area between the basipterygoid processes and parasphenoidrostrum: is a mildly concave subtriangular region (0); forms a deep slot-like cavity that passes posteriorly between the bases of the basipterygoid processes (1). (Mannion et al., 2013:character 48)
83. Occipital region of skull, shape: anteroposteriorly deep, paroccipital processes oriented posterolaterally (0); flat, paroccipital processes oriented transversely (1). (Wilson, 2002:character 54)
84. Occipital condyle, lateral surface of the basioccipital: flat or slightly convex (0); strongly concave (1). (Remes et al., 2009:character 50)
85. Dentary, depth of anterior end of ramus: slightly less than that of dentary at midlength (0); 150% minimum depth (1). (Wilson, 2002:character 55)
86. Dentary, anteroventral margin shape: gently rounded (0); sharply projecting triangular process (1). (Wilson, 2002:character 56)
87. Dentary symphysis, orientation: angled 15° or more anteriorly to (0); or perpendicular to axis of jaw ramus (1). (Wilson, 2002:character 57)
88. Dentary, cross-sectional shape of symphysis: oblong or rectangular (0); subtriangular, tapering sharply towards ventral extreme (1); subcircular (2). (Whitlock, 2011:character 60)
89. Dentary, tuberosity on labial surface near symphysis: absent (0); present (1). (Whitlock, 2011:character 57)
90. Dentary, posteroventral process shape: single (0); divided (1). (D’Emic, 2012:character 10)
91. Mandible, coronoid eminence: strongly expressed, clearly rising above plane of dentigerous portion (0); absent (1). (Whitlock, 2011:character 62)
92. External mandibular fenestra: present (0); absent (1). (Wilson, 2002:character 58)
93. Surangular depth: less than twice (0); or more than two and one-half times maximum depth of the angular (1). (Wilson, 2002:character 59) 48
94. Surangular ridge separating adductor and articular fossae: absent (0); present (1). (Wilson, 2002:character 60)
95. Adductor fossa, medial wall depth: shallow (0); deep, prearticular expanded dorsoventrally (1). (Wilson, 2002:character 61)
96. Splenial posterior process, position: overlapping angular (0); separating anterior portions of prearticular and angular (1). (Wilson, 2002:character 62)
97. Splenial posterodorsal process: present, approaching margin of adductor chamber (0); absent (1). (Wilson, 2002:character 63)
98. Coronoid, size: extending to dorsal margin of jaw (0); reduced, not extending dorsal to splenial (1); absent (2). (Wilson, 2002:character 64)
99. Tooth rows, shape of anterior portions: narrowly arched, anterior portion of tooth rows V-shaped (0); broadly arched, anterior portion of tooth rows U-shaped (1); rectangular, tooth-bearing portion of jaw perpendicular to jaw rami (2). (Wilson, 2002:character 65).
100. Tooth rows, length: extending to orbit (0); restricted anterior to orbit (1); restricted anterior to antorbital fenestra (2); restricted anterior to subnarial foramen (3). (Modifiedfrom Wilson, 2002:character 66)
101. Maxillary teeth shape: straight along axis (0); twisted axially through an arc of 30- 45º: absent (0); present (1). (D’Emic, 2012:character 15)
102. Dentary teeth, number: greater than 20 (0); 10-17 (1); 9 or fewer (2). (Modifiedfrom Wilson, 2002:character73)
103. Replacement teeth per alveolus, number: two or fewer (0); more than four (1). (Wilson, 2002:character 74)
104. Lateral plate: absent (0); present (1). (Upchurch et al., 2004:character 9)
105. Teeth, orientation: perpendicular (0); or oriented anteriorly relative to jaw margin (1). (Wilson, 2002:character 75)
106. Tooth crowns, orientation: aligned along jaw axis, crowns do not overlap (0); aligned slightly anterolingually, tooth crowns overlap (1). (Wilson, 2002:character 69)
107. Tooth crowns, shape: narrow crowns (0); broad crowns (1).
108. Tooth crowns, cross-sectional shape at mid-crown: elliptical (0); D-shaped (1); subcylindrical (2); cylindrical (3). (Wilson, 2002:character 70)
109. SI values for tooth crowns: less than 3.0 (0); 3.0-4.0 (1); 4.0-5.0 (2); more than 5.0 (3). (Upchurch et al., 2004:chs. 67-69)
110. Crown-to-crown occlusion: absent (0); present (1). (Wilson, 2002:character 67)
111. V-shaped wear facets: present (0); absent (1). (Modifiedfrom Wilson, 2002:character 68)
112. Development of the marginal wear facets: well developed (0); slightly developed as marginal facets (1).
113. One high angle wear facet and a second low angle wear facet: absent (0); present (1).
114. Single planar wear facet in labial or lingual surface of the teeth: absent (0); present (1).
115. Marginal tooth denticles: present (0); absent on posterior edge (1); absent on both anterior and posterior edges (2). (Wilson, 2002:character 72) 49
116. Enamel surface texture: smooth (0); wrinkled (1). (Wilson, 2002:character71)
117. Thickness of enamel asymmetric labiolingually: absent (0); present (1). (Whitlock, 2011:character 74)
118. Teeth, longitudinal grooves on lingual aspect: absent (0); present (1). (Wilson, 2002:character 76)
119. Cervical vertebrae, number: 10 or fewer (0); 12 (1); 13-14 (2); 15 (3); 16 or more (4). (Modifiedfrom Wilson, 2002:character 80 and Upchurch et al., 2004:chs. 96-100)
120. Atlas, intercentrum occipital facet shape: rectangular in lateral view, length of dorsal aspect subequal to that of ventral aspect (0); expanded anteroventrally in lateral view, anteroposterior length of dorsal aspect shorter than that of ventral aspect (1). (Wilson, 2002:character 79)
121. Axis, centrum shape: over two and a half times as long as tall (0); less than twice as long as tall (1). (D’Emic, 2012: character 20)
122. Cervical vertebrae, parapophyses, shape and orientation: short and weakly developed, projected laterally or slightly ventrally (0); middle development, ventrally such that the cervical ribs are displaced ventrally around half the height of the centrum (1); well developed, broad and ventrally projected such that cervical ribs are displaced ventrally more than the height of the centrum (2). (ModifiedfromD’Emic, 2012:character 29)
123. Cervical centra, articulations: amphicoelous (0); opisthocoelous (1). (Salgado et al., 1997:character 1 ; Wilson, 2002:character 82; Upchurch, 1998:character 81 and Upchurch et al., 2004:character 103)
124. Cervical centra, ventral surface: is flat or slightly convex transversely (0); transversely concave (1). (Upchurch, 1998:character 84 and Upchurch et al., 2004:character 107)
125. Cervical centra, midline keels on ventral surface: prominent and plate-like (0); reduced to low ridges or absent (1). (Upchurch, 1998:character 83 and Upchurch et al., 2004:character 106)
126. Cervical centra, pleurocoels: absent (0); present with well defined anterior, dorsal, and ventral edges, but not the posterior one (1); present, with well defined edges (2); absent, but with deep lateral fossa which bears small pneumatopores that communicate to the interior pneumatic cavities. (3).
127. Cervical centra, pleurocoels: singles without division (0); with a well defined anterior excavation and a posterior smooth fossa (1); divided by a bone septum, resulting in an anterior and a posterior lateral excavation (2); divided in three or more lateral excavations, resulting in a complex morphology (3); with a well defined anterior excavation and a posterior smooth fossa (Modified from Salgado et al., 1997; Wilson, 2002; Harris, 2006)
128. Cervical vertebrae, well-developed epipophyses: absent (0); present (1).
129. Cervical vertebrae, epipophyses shape: stout, pillar-like expansions above postzygapophyses (0); posteriorly projecting prongs (1). (D’Emic, 2012:character 24) 50
130. Prezygapophyses, anterior process suited ventrolaterally to the articular surface: absent (0); present (1). (Remes et al., 2009:character 79)
131. Cervical vertebrae with an accessory lamina, which runs from the PODL (or slightly anteriorly) up to the SPOL: absent (0); present (1). (ModifiedfromD'Emic, 2012:character 25)
132. Cervical vertebrae, height divided width (measured in its posterior articular surface): higher than 1.1 (0), around 1 (1); between 0.9 and 0.7 (2); smaller than 0.7 (3). (Modified from Wilson, 2002:character 84; Upchurch,1998:character 85 and Upchurch et al., 2004:character 108)
133. Cervical centra, small notch in the dorsal margin of the posterior articular surface: absent (0); present (1). (Carballido et al., 2012)
134. Cervical vertebrae, neural arch lamination: well developed, with well-marked laminae and fossae (0); rudimentary, with diapophyseal laminae absent or very slightly marked (1). (Wilson, 2002:ch, 81)
135. Cervical vertebrae with an accessory lamina, which runs from the postzygodiapophyseal lamina (PODL) up to the spinoprezygapophyseal lamina (SPRL): absent (0); present (1). (Modifiedfrom Sereno et al., 2007:chs. 50, 51; Whitlock, 2011:chs. 78, 96).
136. Cervical centra, internal pneumaticity: absent (0); present with singles and wide cavities (1); present, with several small and complex internal cavities (2). (Modifiedfrom Carballido et al., 2011)
137. Anterior cervical vertebrae, prespinal lamina: absent (0); present (1). (Carballido et al., 2012).
138. Anterior cervical vertebrae, neural spine shape: single (0); bifid (1). (Wilson, 2002:character 72; Upchurch et al., 2004:character 118)
139. Middle and posterior cervical vertebrae, prespinal lamina: absent (0); present (1). (Carballido et al., 2012).
140. Middle cervical vertebrae, lateral fossae on the prezygapophysis process: absent (0); present (1). (Harris, 2006).
141. Middle, cervical vertebrae, height of the neural arch: less than the height of the posterior articular surface (0); higher than the height of the posterior articular surface (1). (Wilson, 2002:character 87; similar Upchurch et al., 2004:111 and 112)
142. Middle cervical centrum, anteroposterior length divided by the height of the posterior articular surface: less than 4 (0); more than 4 (1). (Wilson, 2002:character 74; and Upchurch et al., 2004:character 102).
143. Middle and posterior cervical vertebrae, morphology of the centroprezygapophyseal lamina: single (0); dorsally divided, resulting in a lateral and medial lamina, being the medial lamina linked with the intraprezygapophyseal lamina and not with the prezygapophysis (1); divided, resulting in the presence of a “true” divided centroprezygapophyseal lamina, which is dorsally connected to the prezygapophisis (2). (Carballido et al., 2012).
144. Middle and posterior cervical vertebrae, morphology of the centropostzygapophyseal lamina (CPOL): single (0); divided, with the medial part contacting the intrapostzygapophyseal lamina (1) (Carballido et al., 2012)
145. Middle and posterior cervical vertebrae, articular surface of zygapophyses: flat (0); transversally convex (1). (Upchurch et al., 2004)
146. Middle and posterior cervical vertebrae, prominent triangular flange on the posterior edge of the diapophyseal process (in the PCDL): absent (0); present (1). (Remes et al., 2009: character 78)
147. Middle cervical vertebrae, prezygapophyses position: do not extend beyond the anterior margin of the centrum (0); extends beyond the anterior margin of the centrum (1). (Salgado et al., 1997, character 37)
148. Middle and posterior cervical vertebrae, parapophysis shape: subcircular (0); anteroposteriorly elongate (1). (D’Emic, 2012:character 28)
149. Posterior cervical vertebrae, lateral profile of the neural spine: displays steeply sloping cranial and caudal faces (0); displays steeply sloping cranial face and noticeably less steep caudal margin (1). (Upchurch et al., 2004:character 119)
150. Posterior cervical vertebrae, neural spine shape: not expanded distally (0); expanded but not as much as the width of the centrum (1); laterally expanded, being equal or wider than the vertebral centrum (1). (Modifiedfrom González Riga et al., 2009)
151. Posterior cervical vertebrae, lateral expansion: SPRLs does not contact the lateral margins of the neural spine (0); SPRLs are contacting the lateral margins of the neural spine (1). (Modifiedfrom González Riga and Ortiz, 2014: character 26-27)
152. Posterior cervical and anterior dorsal vertebrae, neural spine shape: single (0); bifid (1). (Wilson, 2002:character 90, Upchurch et al., 2004:character 118)
153. Posterior cervical vertebrae, proportions – ratio total height / centrum length: less than 1.5 (0); more than 1.5 (1). (González Riga et al., 2009:character 32)
154. Posterior cervical and anterior dorsal bifid neural spines, median tubercle: absent (0); present (1).
155. Number of dorsal vertebrae: 14 or more (0); 13 (1); 12 (2); 10 (3). (Modifiedfrom Wilson, 2002:character 91; Upchurch et al. 2004:character 122- 125)
156. Dorsal centra, pleurocoels: absent (0); present (1). (Wilson, 2002:character 78; Upchurch et al. 2004:128)
157. Dorsal vertebrae, transverse processes: are directed laterally or slightly upwards (0); are directed strongly dorsolaterally (1). (Upchurch et al., 2004:character 138)
158. Dorsal vertebrae, distal end of the transverse process: curves smoothly into the dorsal surface of the process (0); is set off from the dorsal surface, the latter having a distinct dorsally facing flattened area (1). (Upchurch et al., 2004:character 140)
159. Anterior dorsal vertebrae, non-bifid neural spine in anterior or posterior view: posses subparallel lateral margins (0); posses lateral margins which slightly diverge dorsally (1); posses lateral margins which strongly diverge dorsally (2); posses lateral margins dorsomedially inclined, that approach each other (3).(Modified 52 from Wilson, 2002:character 107; Upchurch et al., 2004:character 155)
160. Middle to posterior dorsal vertebrae, non-bifid neural spine in anterior or posterior view: posses subparallel lateral margins (0); posses lateral margins which slightly diverge dorsally (1); posses lateral margins which strongly diverge dorsally (2). (Modifiedfrom Wilson, 2002:character 107; Upchurch et al., 2004:character 155)
161. Dorsal centra, pneumatic structures: absent, dorsal centra with solid internal structure (0); present, dorsal centra with simple and big air-spaces (camerate) (1); present, dorsal centra with small and complex air-spaces (polycamerate) (2); present, dorsal centra with small and complex air spaces (semicamellate/camellate) (3). (Modifiedfrom Carballido et al., 2011)
162. Anterior and middle dorsal neural spines, spinoprezygapophyseal lamina (SPRL): absent (0); present (1). (Modifiedfrom Upchurch et al. (2007:character 131).
163. Posterior dorsal neural spines, spinoprezygapophyseal lamina (SPRL): absent (0); present (1). (Modifiedfrom Upchurch et al., 2007:character 132).
164. Dorsal vertebrae, single not bifid neural spines, single prespinal lamina (PRSL): absent (0); present (1). (Modifiedfrom Salgado et al., 1997:character14)
165. Dorsal vertebrae, single not bifid neural spines, single prespinal lamina (PRSL): rough and wide, present in the dorsalmost part of the neural spine (0); rough and wide, extended along almost all neural spine (1); smooth and narrow (2). (Carballido et al., 2012)
166. Dorsal vertebrae with single neural spines, middle single fossa projected through the midline of the neural spine: present (0); absent (1). (Carballido et al., 2012)
167. Dorsal vertebrae with single neural spines, middle single fossa, projected through the midline of the neural spine: relatively wide median simple fossa (0); a thin median simple fossa (1); extremely reduced median simple fossa (2). (Carballido et al., 2012)
168. Anterior dorsal centra, articular face shape: amphicoelous (0); opisthocoelous (1). (Wilson, 2002:character 94; Upchurch et al., 2004:character 104)
169. Anterior and middle dorsal centra, pleurocoels: have rounded caudal margins (0); have tapering, acute caudal margins (1). (Salgado et al., 1997; Upchurch, 1998:character 06; Upchurch et al., 2004:ca 127)
170. Middle dorsal neural arches in lateral view, anterior edge of the neural spine: project anteriorly to the diapophysis (0); converge with the diapophysis (1); project posteriorly to the diapophysis (2). (Carballido et al., 2012)
171. Anterior and middle dorsal vertebrae, zygapophyseal articulation angle: horizontal or slightly posteroventrally oriented (0); posteroventraly oriented (around 30º) (1); strongly posteroventraly oriented (more than 40º) (2). (Carballido et al., 2012)
172. Anterior dorsal vertebrae, neural spine orientation: vertical, or slightly inclined (less than 20º) (0); posterodorsally, more than 20º (1); anteriorly directed (2).
173. Anterior dorsal vertebrae neural spine, triangular aliform processes: absent (0); present but do not project far laterally (not as far as caudal zygapophyses) (1); present and project far laterally (as far as caudal zygapophyses) (2). (Modifiedfrom Wilson, 2002:character 102 and Upchurch et al., 2004:chs. 153-154).
174. Anterior dorsal vertebrae, neural spine minimums width / length: 0.5 or greater (stout and short neural spine) (0); lower than 0.5 (thin and tall neural spines). (Carballido et al., 2017:ch. 174)
175. Anterior dorsal vertebrae, neural spine length (from TPRL to top): less than the height of the centrum (0); slightly higher than the centrum (1); twice or more the height of the centrum (2). (Carballido et al., 2017:ch. 175)
176. Anterior dorsal vertebrae, dorsal edge of the neural spine: flat (0); arrow-shaped (1); convex (2). (Carballido et al., 2017:ch. 176)
177. Posterior dorsal vertebrae, dorsal edge of the neural spine: flat (0); arrow-shaped (1); convex (2). (Carballido et al., 2017:ch. 177)
178. Middle to posterior dorsal centra, ventral surface: convex transversely (0); flattened (1); is slightly concave, sometimes with one or two crests (2). (Upchurch et al., 2004)
179. Middle dorsal vertebrae, hyposphene-hypantrum system: present (0); absent (1). (Modified from Salgado et al., 1997:character 25; Wilson, 2002:character 106; Upchurch et al., 2004:character 145)
180. Posterior dorsal vertebrae, hyposphene-hypantrum system: present and well developed, usually with a rhomboid shape (0); present and weakly developed, mainly as a laminar articulation (1); absent or only present in posteriormost dorsal vertebrae (2). (Carballido et al., 2012)
181. Middle and posterior dorsal vertebrae, transverse processes length: short (0); long (projecting along 1.5 the articular surface width) (1). (Carballido et al., 2012)
182. Mid and posterior dorsal vertebrae with a single lamina (the single TPOL) supporting the hyposphene or postzygapophysis from below: absent (0); present (1). (Modifiedfrom Upchurch et al., 2004:character 146)
183. Middle and posterior dorsal vertebrae, neural canal in anterior view: entirely surrounded by the neural arch (0); enclosed in a deep fossa, enclosed laterally by pedicels (1). (Upchurch et al., 2004:character 136)
184. Middle and posterior dorsal vertebrae, neural spine height: approximately twice the centrum length (0); for times the centrum length (1). (Upchurch et al., 2004)
185. Middle and posterior dorsal neural spines orientation: vertical (0); slightly inclined, with an angle of around 70 degrees (1); strongly inclined, with an angle not bigger than 40 degrees (2). (Modifiedfrom Wilson, 2002:character 104)
186. Middle and posterior dorsal vertebral, central keel: absent (0); present (1). (D’Emic, 2012:character 49)
187. Dorsal vertebrae, height of the neural arch divided the height of the centrum: less than 0.8 (0); more than 0.8 (1). (Pol et al., 2011:character 132)
188. Middle to posterior dorsal vertebrae, pleurocoel dorsal margin: rounded (0); angular (1). (Rauhut et al. 2015:character 346)
189. Middle to posterior dorsal vertebrae, pleurocoel dorsal margin: well below the dorsal margin of the centrum (0); at the level of the dorsal margin of the centrum or higher (1). (Rauhut et al., 2015:character 347)
190. Middle to posterior dorsal vertebrae, small fossa anterior or anteroventral to the pleurocoel: absent (0); present (1). (Rauhut et al. 2015:character 348)
191. Middle and posterior dorsal neural arches, centropostzygapophyseal lamina (CPOL), shape: simple (0); divided (1). (Wilson, 2002:character 95) 54
192. Middle and posterior dorsal neural arches, anterior centroparapophyseal lamina (ACPL): absent (0); present (1). (Wilson, 2002:character 96; Upchurch et al., 2004:character 133)
193. Middle and posterior dorsal neural arches, prezygoparapophyseal lamina (PRPL): absent (0); present (1). (Wilson, 2002:character 97)
194. Middle and posterior dorsal neural arches, posterior centroparapophyseal lamina (PCPL): absent (0); present (1). (Wilson, 2002:character 98, Upchurch et al., 2004:character 137)
195. Middle and posterior dorsal centrum in transverse section (height: width ratio): subcircular (ratio, similar to 1 or a bit higher) (0); slightly dorsoventrally compressed (ratios between 0.8 and 1) (1); strongly compressed (ratios below 0.8) (2). (Modifiedfrom Upchurch et al., 2004)
196. Middle and posterior dorsal vertebrae neural spine, triangular aliform processes: absent (0); present but do not project far laterally (not as far as caudal zygapophyses) (1); present and project far laterally (as far as caudal zygapophyses) (2). (Modifiedfrom Wilson, 2002:character 102 and Upchurch et al., 2004:chs. 153-154).
197. Middle and posterior dorsal vertebrae, spinodiapophyseal lamina (SPDL): absent (0); present (1). (Upchurch et al., 2004:character 157)
198. Middle and posterior dorsal vertebrae, accessory spinodiapophyseal lamina (SPDL): absent (0); present (1). (Upchurch et al., 2004:character 151)
199. Dorsal vertebrae, spinodiapophyseal webbing: lamina follows curvature of neural spine in anterior view (0); lamina "festooned" from spine, dorsal margin does not closely follow shape of neural spine and diapophysis (1). (Whitlock, 2011:character104)
200. Anterior dorsal vertebrae, spinopostzygapophyseal lamina (SPOL): absent (0); present (1). (Upchurch et al., 2007:character133)
201. Middle and posterior dorsal neural spines, lateral spinopostzygapophyseal lamina (lSPOL): absent (0); present (1). (Wilson, 2002: 100; Upchurch et al., 2004:character 159)
202. Middle and posterior dorsal neural arches, spinodiapophyseal lamina (SPDL) and spinopostzygapophyseal lamina (lSPOL) contact: absent (0); present (1). (Wilson, 2002:character 101)
203. Middle and posterior dorsal vertebrae, spinodiapophyseal (SPDL) and spinopostzygapophyseal lamina (lSPOL) contact: ventral, well separated from the triangular aliform process (0); dorsal, forms part of the triangular aliform process (1). (Carballido et al., 2012)
204. Middle and posterior dorsal vertebrae, height of neural arch below the postzygapophyses (pedicel): less than height of centrum (0); subequal to or greater than height of centrum (1). (Whitlock, 2011:character 109)
205. Posterior Dorsal vertebrae, medial spinopostzygapophyseal lamina (mSPOL): absent (0); present and forms part of the median posterior lamina (1). (Carballido et al., 2012)
206. Posterior dorsal vertebrae, transverse processes: lie posterior, or posterodorsal, to the parapophysis (0); lie vertically above the parapophysis (1). (Upchurch et al., 2004:character 139) 55
207. Posterior dorsal centra, articular face shape: amphicoelous (0); slightly opisthocoelous (1); opisthocoelous (2). (Modifiedfrom Wilson, 2002:character 105)
208. Posterior dorsal vertebrae, neural spine: narrower transversely than anteroposteriorly (0); broader transversely than anteroposteriorly (1). (Wilson, 2002: character 92)
209. Posterior dorsal vertebra, posterior centrodiapophyseal lamina (PCDL): has an unexpanded ventral tip (0); expands and may bifurcate toward its ventral tip (1). (Salgado et al., 1997)
210. Cervical ribs, distal shafts of longest cervical ribs: are elongate and form overlapping bundles (0); are short and do not project beyond the caudal end of the centrum to which they are attached (1). (Wilson, 2002:character 140)
211. Cervical ribs, angle between the capitulum and tuberculum: greater than 90°, so that the rib shaft lies close to the ventral edge of the centrum (0); less than 90°, so that the rib shaft lies below the ventral margin of the centrum (1). (Wilson, 2002:character 139)
212. Dorsal ribs, proximal pneumatopores: absent (0); present (1). (Wilson, 2002:character 141)
213. Anterior dorsal ribs, cross-sectional shape: subcircular (0); plank-like, anteroposterior breadth more than three times mediolateral breadth (1). (Wilson, 2002).
214. Sacral vertebrae, number: 3 or fewer (0); 4 (1); 5 (2); 6 (3). (Wilson, 2002:character 108)
215. Sacrum, sacricostal yoke: absent (0); present (1). (Wilson, 2002:character 109)
216. Sacral vertebrae contributing to acetabulum: numbers 1-3 (0); numbers 2-4 (1). (Wilson, 2002:character 110)
217. Sacral neural spines length: approximately twice length of centrum (0); approximately four times length of centrum (1). (Wilson, 2002:character 111)
218. Sacral ribs, dorsoventral length: low, not projecting beyond dorsal margin of ilium (0); high extending beyond dorsal margin of ilium (1). (Wilson, 2002:character 112)
219. Pleurocoels in the lateral surfaces of sacral centra: absent (0); present (1). (Upchurch et al., 2004:character 165)
220. Caudal vertebrae, number: 35 or fewer (0); 40 to 55 (1); increased to 70-80 (2). (Wilson, 2002:character114)
221. Caudal bone texture: solid (0); spongy (camellate), with large internal cells (1). (Wilson, 2002:character 113)
222. Anterior caudals, pneumatized neural arch: absent (0); present (1). 56
223. Caudal transverse processes: persist through caudal 20 or more posteriorly (0); disappear by caudal 15 (1); disappear by caudal 10(2). (Wilson, 2002:character 115)
224. First caudal centrum anterior articular surface: flat (0); concave (1); convex (2).
225. First caudal centrum, posterior articular surface: flat (0); concave (1); convex (2). The first caudal vertebra of *Patagotitan* has an unusual morphology having a flat anterior articulation surface and a markedly convex posterior one. Therefore, the character reflecting the first caudal centrum morphology (platycoelous, procoelous or biconvex) was split in two characters, one for the anterior articular surface and the second one for the posterior articular surface morphology.
226. First caudal neural arch, coel on lateral aspect of neural spine: absent (0); present (1). (Wilson, 2002:character 117)
227. Anterior caudal vertebrae (mainly the first and second): ventral bulge on transverse process: absent (0); present (1). (D’Emic, 2012:character 52)
228. Anterior caudal vertebrae, lateral pneumatic foramen: absent (0); present (1). The derived state is used even for small foramina. (Modified from D’Emic, 2012:character 56)
229. Posteriormost anterior and middle caudal vertebrae, transverse processes orientation: perpendicular (0); swept backwards, reaching the posterior margin of the centrum (1). (D’Emic, 2012:character 59)
230. Anterior caudal vertebrae, transverse processes: ventral surface directed laterally or slightly ventrally (0); directed dorsally (1). (Whitlock, 2011:character 125)
231. Anterior caudal centra (excluding the first), articular face shape: amphiplatyan or amphicoelous (0); procoelous/distoplatyan (1); slightly procoelous (2); procoelous (3); posterior surface markedly more concave than the anterior one (4). (Modifiedfrom González Riga et al, 2009)
232. Anterior caudal centra, large pneumatic foramen in lateral aspect (pleurocoels): absent or reduced (0); present (1). The derived state is solely used when the lateral pneumatic foramen is large, occupying much of the centrum, as those typically observed in several diplodocids. (Modified from Wilson, 2002:character 119)
233. Anterior caudal vertebrae, ventral surfaces: convex transversely (0); concave transversely (1). (Upchurch et al., 2004:character 182)
234. Anterior and middle caudal vertebrae, ventrolateral ridges: absent (0); present (1). (Upchurch et al., 2004:character 183)
235. Anterior and middle caudal vertebrae, triangular lateral process on the neural spine: absent (0); present (1). (Whitlock, 2011:character 123)
236. Anterior caudal transverse processes shape: triangular, tapering distally (0); "winglike", not tapering distally (1); similar to the wing-shape process (not tapering distally), but with the high lateral margin dorsomedially oriented instead of vertical. (Modified from Wilson, 2002:character 128, adding one character for describing the high processes observed in some titanosauriforms.)
237. Anterior caudal neural spines, transverse shape: anteroposteriorlly long and lateromedially thin neural spines (0); square (length and width similar) (1); laterally expanded, around 1.5 times wider than long (2); + shaped. (modifiedfrom Wilson, 2002:character 126)
238. Anterior caudal transverse processes, proximal depth: shallow, on centrum only (0); deep, extending from centrum to neural arch (1). (Wilson, 2002:character 127)
239. Anterior caudal transverse processes, diapophyseal laminae (ACDl, PCDL, PRDL, PODL): absent (0); present (1). (Wilson, 2002:character 129)
240. Anterior caudal transverse processes, anterior centrodiapophyseal lamina (ACDL), shape: single (0); divided (1). (Wilson, 2002:character 130)
241. Anterior caudal vertebrae, hyposphene ridge: absent (0); present (1). (Upchurch et al., 2004:character 187) 57
242. Anterior caudal centra, length: approximately the same (0); or doubling over the first 20 vertebrae (1). (Wilson, 2002:character 120)
243. Anterior caudal neural arches, spinoprezygapophyseal lamina (SPRL): absent, or present as small short ridges that rapidly fade out into the anterolateral margin of the spine (0); present, extending onto lateral aspect of neural spine (1); present, well developed and extending onto the anterior or anterolateral edges of the neural spine (2)(Modified from Wilson, 2002:character 121). A third state was incorporated in order to include the morphology observed in some taxa in which the SPRL is well developed, but is not extending into the lateral aspect of the neural spine, as is the case of Patagotitan.
244. Anterior caudal neural arches, spinodiapophyseal lamina (SPDL): absent (0); present (1). In titanosaurs the SPDL, when present, is extending from the diapophyseal section of the transverse process (the dorsalmost part of it) up to the neural spine.
245. Anterior caudal neural arches, spinoprezygapophyseal lamina (SPRL)- spinopostzygapophyseal lamina (SPOL) contact: absent (0); present, forming a prominent lamina on lateral aspect of neural spine (1). (Wilson, 2002:character 122)
246. Anterior caudal neural arches, prespinal lamina (PRSL): absent (0); present (1). (Wilson, 2002:character 123)
247. Anterior caudal vertebrae, ventral and medially placed SPRL, usually described as bifurcated PRSL: absent (0); present (1). This character was originally proposed as an autapomorphy of Futalognkosaurus but is not just restricted to this sauropod.
248. Anterior caudal prespinal lamina (PRSL), triangular shaped product of a dorsal expansion of it: absent (0); present (1).
249. Anterior caudal vertebrae, pair thin laminae that are bounding the prespinal laminae and that diverge dorsally: absent (0); present (1). This character was initially proposed as an autapomorphy of *Bonitasaura* but is present in some other sauropods, such as Patagotitan.
250. Middle caudal centra, shape: cylindrical (0); with flat ventral margin (1); quadrangular, flat ventrally and laterally (2); trapezoidal (laterally compressed forming a shallow fossa) (3). (Modified from Wilson, 2002: character 131, Carballido et al., 2020: character 250 and Hechenleitner et al., 2020: character 250).
251. Anterior and middle caudal centra, ventral longitudinal hollow: absent (0); present (1). (Wilson, 2002:character 132)
252. Middle caudal centra, articular face shape: amphiplatyan or amphicoelous (0); procoelous/distoplatyan (1); slightly procoelous (2); procoelous (3). (González Riga et al., 2009)
253. Posteriormost anterior and middle caudal vertebrae, location of the neural arches: over the midpoint of the centrum with approximately subequal amounts of the centrum exposed at either end (0); on the anterior half of the centrum (1). (Upchurch et al., 2004:character 185)
254. Anterior caudal vertebrae, anterior face of the centrum strongly inclined anteriorly: absent (0); present (1). (Santucci and Arruda Campos, 2011: character 256)
255. Middle caudal vertebrae, with the anterior face strongly inclined anteriorly: absent (0); present (1). 58
256. Middle caudal vertebrae, height of the pedicels below the prezygapophysis: low with curved anterior edge of the pedicel (0); high with vertical anterior edge of the pedicel (1). (Carballido et al., 2012)
257. Middle caudal vertebrae, orientation of the neural spines: anteriorly (0); vertical (1); slightly directed posteriorly (2); strongly directed posteriorly (3). (Modified from Wilson, 2002:character 133)
258. Posterior caudal vertebrae, neural spine strongly displaced posteriorly: absent (0); present (1). (Carballido et al., 2012).
259. Middle caudal vertebrae, ratio of centrum length to centrum height: less than 2, usually 1.5 or less (0); 2 or higher (1). (Upchurch et al., 2004:character 179)
260. Anterior-posterior caudal vertebrae (those with still well developed neural spine), neural spine orientation: vertical (0); slightly directed posteriorly (1); strongly directed posteriorly (2). (Carballido et al., 2012)
261. Posterior caudal centra, articular face shape: amphyplatic (0); procoelous (1); opisthocoelous (2). (Modified from González Riga et al., 2009)
262. Posterior caudal centra, shape: cylindrical (0); dorsoventrally flattened, breadth at least twice height (1). (Wilson, 2002:character 135)
263. Posterior caudal vertebrae, ratio of length to height: less than 5, usually 3 or less (0); 5 or higher (1). (Upchurch et al., 2004:character 180)
264. Distalmost caudal centra, articular face shape: platycoelous (0); biconvex (1). (Wilson, 2002:character 136)
265. Distalmost biconvex caudal centra, number: 10 or fewer (0); more than 30 (1). (Wilson, 2002:character 137)
266. Distalmost biconvex caudal centra, length-to height ratio: less than 4 (0); greater than 5 (1). (Wilson, 2002:character 138)
267. Forked chevrons with anterior and posterior projections: absent (0); present (1). (Wilson, 2002:character 143)
268. Forked chevrons, distribution: distal tail only (0); throughout middle and posterior caudal vertebrae (1). (Wilson, 2002:character 144)
269. Chevrons, crus bridging dorsal margin of haemal canal: present (0); absent (1). (Wilson, 2002:character 145)
270. Chevron haemal canal, depth: short, approximately 25% (0); or long, approximately 50% chevron length (1). (Wilson, 2002:character 146)
271. Chevrons: persisting throughout at least 80% of tail (0); disappearing by caudal 30 (1). (Wilson, 2002:character 147)
272. Posterior chevrons, distal contact: fused (0); unfused (open) (1). (Wilson, 2002:character 148)
273. Posture: bipedal (0); columnar, obligatory quadrupedal posture (1). (Wilson, 2002:character 149) Scapular girdle
274. Scapular acromion process, size: Narrow (0); broad, width more than 150% minimum width of blade (1). (Wilson, 2002:character 150)
275. Scapular blade, orientation respect to coracoid articulation: perpendicular (0); forming a 45º angle (1). (Wilson, 2002:character 151)
276. Scapular blade, distal expansion: absent (0); present (1).
277. Scapular blade, shape: acromial edge not expanded (both edges are running parallel to each other) (0); rounded expansion on acromial side (1); racquet-shaped (2): marked distal expansion due to the posterodorsal orientation of the dorsal edge (3). (Wilson, 2002:character 152; as modified by Carballido et al., 2017:ch. 277)
278. Scapula, acromion process dorsal margin: concave or straight (0); with V-shaped concavity (1); with U-shaped concavity (2). (Sereno et al., 2007: 88)
279. Scapula, highest point of the dorsal margin of the blade: lower than the dorsal margin of the proximal end (0); at the same height than the dorsal margin of the proximal end (1); higher than the dorsal margin of the proximal end (2). (Carballido et al., 2012 from Mannion, 2009)
280. Scapula, development of the acromion process: undeveloped (0); well developed (1). (Carballido et al., 2012)
281. Scapular length/minimum blade breadth: 5.5 or less (0); 5.5 or more (1). (Carballido et al., 2012)
282. Scapula, ventral margin with a well-developed ventromedial process: absent (0); present, having only one (1); present, having two processes (2). Onestatewasadded. (Modified from Carballido et al., 2011)
283. Scapular, acromial process position: lies nearly glenoid level (0); lies nearly midpoint scapular body (1). (Carballido et al., 2012)
284. Scapular acromion length: less than 1/2 scapular length (0); at least 1/2 scapular length (1). (Mannion et al., 2012:character168)
285. Glenoid scapular orientation: relatively flat or laterally facing (0); strongly bevelled medially (1). (Wilson, 2002:character 153)
286. Scapular blade, cross-sectional shape at base: flat or rectangular (0); D-shaped (1). (Wilson, 2002:character 154)
287. Coracoid, proximodistal length: less than the length of scapular articulation (0); approximately twice the length of scapular articulation (1). (Wilson, 2002:character 155)
288. Coracoid, anteroventral margin shape: rounded (0); rectangular (1). (Wilson, 2002:character 156)
289. Dorsal margin of the coracoid in lateral view: reaches or surpasses the the level of the dorsal margin of the scapular expansion (0); lies below the level of the scapular proximal expansion and separated from the latter by a V-shaped notch (1). (Upchurch et al., 2004:character 207)
290. Coracoid, Infraglenoid deep groove: absent (0); present (1).
291. Coracoid, infraglenoid lip: absent (0); present (1). (Wilson, 2002:character 157)
292. Sternal plate, shape: posterolateral margin curved (0); posterolateral margin expanded as a corner (1). (D’Emic, 2012:character 76)
293. Sternal plate, shape: oval (0); crescentic (1). (Wilson, 2002:character 158)
294. Prominent posterolateral expansion of the sternal plate producing a kidney-shaped profile in dorsal view: absent (0); present (1). (Upchurch et al., 2004:character 211)
295. Prominent parasagittal oriented ridge on the dorsal surface of the sternal plate: absent (0); present (1). (Upchurch et al., 2004::character 212)
296. Ridge on the ventral surface of the sternal plate: absent (0); present (1). (Upchurch et al., 2004:character 213)
297. Ratio of maximum length of sternal plate to the humerus length: less than 0,75, usually less than 0,65 (0); greater than 0,75 (1). (Upchurch et al., 2004:character 209)
298. Humerus, strong posterolateral bulge around the level of the deltopectoral crest: absent (0); present (1). (D’Emic, 2012:character 80)
299. Humerus, radial and ulnar condyles shape: radial condyle divided on anterior face by a notch (0); undivided (1). (D’Emic, 2012:character 83)
300. Humerus-to-femur ratio: less than 0.60 (0); 0.60 to 0.69 (1); 0.70 to 0.90 (2); greater than 0.90 (3) (Modified from Upchurch et al., 2004: character 216, Carballido et al., 2017: character 300 andHechenleitner et al., 2020: character 300).
301. Humeral deltopectoral attachment, development: prominent (0); reduced to a low crest or ridge (1). (Wilson, 2002:character160)
302. Humeral deltopectoral crest, shape: relatively narrow throughout length (0); markedly expanded distally (1). (Wilson, 2002:character161)
303. Humeral midshaft cross-section, shape: circular (0); elliptical (1). (Mannion et al, 2011:character 170)
304. Humerus, RI (sensu Wilson and Upchurch, 2003): Gracile (less than 0,27) (0); medium (0,28-0,32) (1); Robust (more than 0,33) (2). (Carballido et al., 2012)
305. Humeral distal condyles, articular surface shape: restricted to distal portion of humerus (0); exposed on anterior portion of humeral shaft (1). (Wilson, 2002:character 163)
306. Humeral distal condyle, shape: divided (0); flat (1). (Wilson, 2002:character 164)
307. Humeral, lateral margin: medially deflected (0); almost straight until the half-length or even more (1); almost straight until the proximal third of the total length of the humerus (2). (Carballido et al., 2012)
308. Humeral proximolateral corner, shape: rounded, the dorsal surface is well convex (0); pronounced / square, the dorsal surface low, almost flat (1). (Wilson, 2002:character 159)
309. Ulnar proximal condyle, shape: subtriangular (0); triradiate, with deep radial fossa (1). (Wilson, 2002:character 165)
310. Ulnar proximal condylar processes, relative lengths: subequal (0); unequal, anterior arm longer (1). (Wilson, 2002:character 166)
311. Ulnar olecranon process, development: prominent, projecting above proximal articulation (0); rudimentary, level with proximal articulation (1). (Wilson, 2002:character 167) 61
312. Ulna, length-to-proximal breadth ratio: gracile (0); stout (1). (Wilson, 2002:character 168)
313. Radial distal condyle, shape: round (0); subrectangular, flattened posteriorly and articulating in front of ulna (1). (Wilson, 2002:character 169)
314. Radius, distal breadth: slightly larger than midshaft breadth (0); approximately twice midshaft breadth (1). (Wilson, 2002:character 170)
315. Radius, distal condyle orientation: perpendicular to long axis of shaft (0); bevelled approximately 20º proximolaterally relative to long axis of shaft (1). (Wilson, 2002:character 171)
316. Carpal bones, number: 3 or more (0); 2 or fewer (1). (Wilson, 2002:character 173)
317. Carpal bones, shape: round (0); block-shaped, with flattened proximal and distal surfaces (1). (Wilson, 2002:character 174)
318. Metacarpus, shape: spreading (0); bound, with sub-parallel shafts and articular surfaces that extend half their length (1). (Wilson, 2002:character 175)
319. Metacarpals, shape of proximal surface in articulation: gently curving, forming a 90° arc (0); U-shaped, subtending a 270° arc (1). (Wilson, 2002:character 176)
320. Longest metacarpal-to-radius ratio: close to 0.3 (0); 0.45 or more (1). (Wilson, 2002:character 177)
321. Metacarpal I, length: shorter than metacarpal IV (0); longer than metacarpal IV (1). (Wilson, 2002:character 178)
322. Metacarpal I, distal condyle shape: divided (0); undivided (1). (Wilson, 2002:character 179)
323. Metacarpal I distal condyle, transverse axis orientation: bevelled approximately 20º respect to axis of shaft (0); proximodistally or perpendicular with respect to axis of shaft (1). (Wilson, 2002:character 180)
324. Manual digits II and III, phalangeal number: 2- 3-4-3-2 or more (0); reduced, 2-2-2- 2-2 or less (1); absent or unossified (2). (Wilson, 2002:character 181)
325. Manual phalanx I.1, shape: rectangular (0); wedge-shaped (1). (Wilson, 2002:character 182)
326. Manual nonungual phalanges, shape: longer proximodistally than broad transversely (0); broader transversely than long proximodistally (1). (Wilson, 2002:character 183)
327. Pelvis, anterior breadth: narrow, ilia longer anteroposteriorly than distance separating preacetabular processes (0); broad, distance between preacetabular processes exceeds anteroposterior length of ilia (1). (Wilson, 200:character 184)
328. Ilium, ischial peduncle size: large, prominent (0); low, rounded (1). (Wilson, 2002:character 185)
329. Ilium, dorsal margin shape: flat (0); semicircular (1). (Wilson, 2002:character 186)
330. Illiun, preacetabular ventral margin shape: straight (0), concave (1); with a convex ventral bump (2)
331. Ilium, preacetabular process shape: pointed, arching ventrally (0); semicircular, with posteroventral excursion of cartilage cap (1). (Wilson, 2002:character 188)
332. Ilium, preacetabular process orientation: anterolateral to body axis (0); perpendicular to body axis (1). (Wilson, 2002:character 189)
333. Highest point on the dorsal margin of the ilium: lies caudal to the base of the pubic process (0); lies cranial to the base of the pubic process (1). (Upchurch et al., 2004:character 245)
334. Pubis length respect to ischium: pubis slightly smaller or subequal to ischium (0); pubis larger (120% +) than ischium (1). (Carballido et al., 2012)
335. Pubis, ambiens process development: small, confluent with anterior margin of pubis prominent, (0); projects anteriorly from anterior margin of pubis (1). (Wilson, 2002:character 189)
336. Pubic apron, shape: flat (straight symphysis) (0); canted anteromedially (gentle S-shaped symphysis) (1). (Wilson, 2002:character 190).
337. Puboischial contact, length: approximately one third total length of pubis (0); onehalf total length of pubis (1). (Wilson, 2002:character 191)
338. Ischium, acetabular articular surface: maintains approximately the same transverse width throughout its length (0); is transversely narrower in its central portion and strongly expanded as it approaches the iliac and pubic articulations (1). (Mannion et al., 2013:character 180)
339. Ischium, iliac peduncle with constriction or "neck": absent (0); present (1). (Whitlock, 2011:character 173).
340. Ischium, elongate muscle scar on proximal end: absent (0); present (1). (Whitlock, 2011:character 174)
341. Ischial blade, shape: emarginate distal to pubic peduncle (0); no emargination distal to pubic peduncle (1). (Wilson, 2002:character 193)
342. Ischia pubic articulation: less or equal to the anteroposterior length of pubic pedicel (0); greater than the anteroposterior length of pubic pedicel (1). (Salgado et al., 1997)
343. Ischia, anteroposterior pubic pedicel width divided the total length of the ischium: less than 0.5 (0); 0.5 or larger (1). (Carballido et al., 2012).
344. Ischial distal shaft, shape: triangular, depth of ischial shaft increases medially (0); bladelike, medial and lateral depths subequal (1). (Upchurch et al., 2004:character 194)
345. Ischial distal shafts, cross-sectional shape: V-shaped, forming an angle of nearly 50º with each other (0); flat, nearly coplanar (1). (Wilson, 2002:character 195)
346. Ischia, distal end: is only slightly expanded (0); is strongly expanded dorsoventrally (1). (Upchurch, 1998:character 183)
347. Ischium, angle formed between the shaft and the acetabular line: forming an almost right angle (80-110°) (0) or; a close angle (less than 70°) (1). (Carballido et al., 2012)
348. Ischial tuberosity: absent (0); present (1). The tuberosity, noted by Otero (2010) for the ischium of *Neuquensaurus* (Otero, 2010:Fig. 8) and is present in other taxa, such as *Patagotitan*, *Bonitasaura*, *Futalognkosaurus*, *Alamosaurus* and *Neuquensaurus*.
349. Femur, longitudinal ridge on the anterior face: absent (0); present (1). (D’Emic, 2012:character 107)
350. Femur, fibular condyle: well developed, having a similar posterior projection than the tibial one (0); much shorter than the tibial condyle (1). The fibular condyle of *Patagotitan* and*Bonitasaura* is reduced in its posterior projection with respect to that of most other sauropods, which have fibular and tibial condyles that are almost equally posteriorly projected.
351. Femur, epicondyle development: well developed (0); reduced, almost absent (1). In *Patagotitan* the epicondyle is extremely developed and notorious in posterior and distal view, as a minor step laterally projected. In contrast, in some titanosaurs the epicondyle is almost imperceptible, as is the case of *Dreadnoughtus*, *Opsithocoelicaudia*, *Neuqunesaurus,* and *Saltasaurus*.
352. Femur, fourth trochanter position: almost at the half of the femur (0); in the proximal third of the femur (1). The fourth trochanter of *Patagotitan* is positioned around the proximal third of the total femur length, similar to the position observed in *Futalognkosaurus*, *Bonitasaura*, and some other non-Lognkosauria as *Rapetosaurus*, *Saltasaurus* and *Neuquensaurus*. In contrast, the fourth trochanter of most sauropods is around the half of the total femur length, being even lower in *Opisthocoelicaudia*.
353. Femur, fourth trochanter development: prominent (0); reduced to crest or ridge (1); extremely reduced (2). (Modifiedfrom Wilson, 2002:character 196, following to Whitlock, 2011:character 186)
354. Femur, lesser trochanter: present (0); absent (1). (Wilson, 2002:character 197)
355. Femur midshaft, transverse diameter: subequal to anteroposterior diameter (0); 125- 150% anteroposterior diameter (1); at least 185% anteroposterior diameter (2). (Wilson, 2002:character 198)
356. Femur, lateral bulge (marked by the lateral expansion and a dorsomedial orientation of the laterodorsal margin of the femur, which starts below the femur head ventral margin): absent (0); present (1). (Salgado et al., 1997)
357. Femur, pronounced ridge on posterior surface between greater trochanter and head: absent (0); present (1). (Whitlock, 2011:character 181)
358. Femur head position: perpendicular to the shaft, rises at the same level of the greater trochanter (0); dorsally directed, rises well above the level of the greater trochanter (1). (Modifiedfrom Upchurch et al., 2004:character 263)
359. Femur, distal condyles relative transverse breadth: subequal (0); tibial much broader than fibular (1). (Wilson, 2002:character 2000)
360. Femur, distal condyles orientation: perpendicular or slightly bevelleddorsolaterally (0); or bevelleddorsomedially approximately 10 relative to femoral shaft (1). (Wilson, 2002:character 201) 64
361. Femur, distal condyles articular surface shape: restricted to distal portion of femur (0); expanded onto anterior portion of femoral shaft (1). (Wilson, 2002:character 202)
362. Situation of the femoral fourth trochanter: on the caudal surface of the shaft, near the midline (0); on the caudomedial margin of the shaft (1). (Upchurch et al., 2004:character 268)
363. Tibial proximal condyle, shape: narrow, long axis anteroposterior (0); expanded transversely, condyle subcircular (1). (Wilson, 2002:character 203)
364. Tibial cnemial crest, orientation: projecting anteriorly (0); or laterally (1). (Wilson, 2002:character 204)
365. Tibia, distal breadth: approximately 125% (0); more than twice midshaft breadth (1). (Wilson, 2002:character 205)
366. Tibial distal posteroventral process, size: broad transversely, covering posterior fossa of astragalus (0); shortened transversely, posterior fossa of astragalus visible posteriorly (1). (Wilson, 2002:character 206)
367. Fibula, proximal tibial scar, development: not well-marked (0); well-marked and deepening anteriorly (1). (Wilson, 2002:character 207)
368. Fibula, lateral trochanter: absent (0); present (1). (Wilson, 2002:character 208)
369. Fibular distal condyle, size: subequal to shaft (0); expanded transversely, more than twice midshaft breadth (1). (Wilson, 2002:character 209)
370. Fibular, proximal end, anterior crest: absent or poorly developed (0); well developed creating an interlocking proximal crus (1). (D’Emic, 2012:character 111)
371. Fibula, shaft shape: straight, or slightly sigmoidal (0); sigmoid, such that the proximal and distal faces are angled relative to midshaft (1). (D’Emic, 2012:character 113)
372. Astragalus, shape: at least 1.5 times wider than anteroposteriorly long (0); anteroposterior and transverse dimensions subequal (1). (D’Emic, 2012:character 115)
373. Astragalus, shape: rectangular (0); wedge shaped, with reduced anteromedial corner (1). (Wilson, 2002:character210)
374. Astragalus, fibular facet: faces laterally (0); faces posterolaterally, anterior margin visible in posterior view (1). (Whitlock, 2011:character 186)
375. Astragalus, foramina at base of ascending process: present (0); absent (1). (Wilson, 2002:character 211)
376. Astragalus, ascending process length: limited to anterior two-thirds of astragalus (0); extending to posterior margin of astragalus (1). (Wilson, 2002:character 212)
377. Astragalus, posterior fossa shape: undivided (0); divided by vertical crest (1). (Wilson, 2002:character 213)
378. Astragalus, transverse length: 50% more than (0); or subequal to proximodistal height (1). (Wilson, 2002:character 214)
379. Calcaneum: present (0); absent or unossified (1). (Wilson, 2002:character 215) 65
380. Distal tarsals 3 and 4: present (0); absent or unossified (1). (Wilson, 2002:character 216)
381. Metatarsus, posture: bound (0); spreading (1). (Wilson, 2002:character 217)
382. Metatarsal I proximal condyle, transverse axis orientation: perpendicular to (0); angled ventromedially approximately 15º to axis of shaft (1). (Wilson, 2002:character 218)
383. Metatarsal I distal condyle, transverse axis orientation: perpendicular to (0); angled dorsomedially to axis of shaft (1). (Wilson, 2002:character 219)
384. Metatarsal III length divided by metatarsal I length: less than 1.3 (0); more than 1.3 (1). (González Riga et al., 2016:character 331)
385. Longestmetatarsal: metatarsal III (0); metatarsal IV (1). (González Riga et al., 2016:character 334)
386. Metatarsal I distal condyle, posterolateral projection: absent (0); present (1). (Wilson, 2002:character 220)
387. Metatarsal I, minimum shaft width: less than that of metatarsals II-IV (0); or greater than that of metatarsals IIIV (1). (Wilson, 2002:character 221)
388. Metatarsal I and V proximal condyle, size: smaller than (0); or subequal to those of metatarsals II and IV (1). (Wilson, 2002:character 222)
389. Metatarsal III length: more than 30% (0); or less than 25% that of tibia (1). (Wilson, 2002:character 223)
390. Metatarsals III and IV, minimum transverse shaft diameters: subequal to (0); or less than 65% that of metatarsals I or II (1). (Wilson, 2002:character 224)
391. Metatarsal IV, proximomedial end, shape: flat or slightly concave (0); possesses a distinct embayment (1). (D’Emic, 2012:character 117)
392. Metatarsal IV, distal end, orientation: roughly perpendicular to long axis of bone (0); bevelled upwards medially (1). (D’Emic, 2012:character 118)
393. Metatarsal V, length: shorter than (0); or at least 70% length of metatarsal IV (1). (Wilson, 2002:character 225)
394. Pedal non-ungual phalanges, shape: longer proximodistally than broad transversely (0); broader transversely than long proximodistally (1). (Wilson, 2002:character 226)
395. Pedal digits II-IV, penultimate phalanges, development: subequal in size to more proximal phalanges (0); rudimentary or absent (1). (Wilson, 2002:character 227)
396. Pedal unguals, orientation: aligned with (0); or deflected lateral to digit axis (1). (Wilson, 2002:character 228)
397. Pedal digit I ungual, length relative to pedal digit II ungual: subequal (0); 25% larger than that of digit II (1). (Wilson, 2002:character 229)
398. Pedal digit I ungual, length: shorter (0); or longer than metatarsal I (1). (Wilson, 2002:character 230)
399. Pedal ungual I, shape: broader transversely than dorsoventrally (0); sickle-shaped, much deeper dorsoventrally than broad transversely (1). (Wilson, 2002:character 231) 66
400. Pedal ungual II-III, shape: broader transversely than dorsoventrally (0); sickle-shaped, much deeper dorsoventrally than broad transversely (1). (Wilson, 2002:character 232)
401. Pedal digit IV ungual, development: subequal in size to unguals of pedal digits II and III (0); rudimentary or absent (1). (Wilson, 2002:character 233)
402. Unguals of pedal digit II and III, proximal dimensions: as broad as deep (0); significantly broader than deep (1). (Allain and Aquesbi, 2008:character 253)
403. Number of phalanges in pedal digit II: 3 (0); 2 (1). (González Riga et al., 2016:character 348)
404. Number of phalanges in pedal digit III: 4 (0); 3 (1). (González Riga et al., 2016:character 349)
405. Number of phalanges in pedal digit IV: 3 or more (0); 2 (1); 1 (2). (González Riga et al., 2016:character 350) 406. Postorbital, excluded from the infratemporal fenestra due to the articulation of the jugal with the squamosal: absent (0); present (1). (New)
406. Postorbital, excluded from the infratemporal fenestra due to the articulation of the jugal with the squamosal: absent (0), present (1). (Canudo et al., 2018)
407. Squamosal, ventral shape: thin (0); broad (1). (Canudo et al., 2018)
408. Preantorbital fenestra development: small, differentiated from the posterior maxillary foramen in its direction (see Wilson and Sereno, 1998) (0); laterally opened middle sized fenestra (1); laterally opened large fenestra (2). (Canudo et al., 2018)
409. Mid- and posterior dorsal neural arches, centroprezygapophyseal fossa depth: shallow or absent (0); deep, passing nearly all the way through the neural arch. (Wilson and Allain, 2015: ch. 101)
410. Mid- Posterior dorsal vertebrae, parapophysis, position with respect to prezygapophyses: at the same level or below (0); well above (1). (Wilson and Allain, 2015: ch. 100)
411. Posterior dorsal neural arches, centroprezygapophyseal lamina (CPRL), shape: single (0); divided (1). (Wilson and Allain, 2015: ch. 107)
412. Posterior dorsal neural arches, spinoparapophyseal lamina (SPPL): absent (0); present (1). (Wilson and Allain, 2015: ch. 109)
413. Middle caudal vertebrae, prezygapophyses orientation: anterodorsally oriented (around 45 degrees) (0); anteriorly oriented (nearly horizontal) (1). (Canudo et al., 2018: ch 413)
414. Position of the single, ventral process: below the acromion (0); on the scapular blade (posterior to the acromion) (1). This character was modified. Taxa without ventral process are scored as missing data (?). Taxa without two ventral process are scored as ambiguous. (Modified Carballido et al., 2019: ch. 414, based on Mannion et al., 2012)
415. Illium, postacetabular posteroventral edge: open concave (0); U-shaped notch (1); horizontal and low V-shaped notch (2). (Carballido et al., 2019: ch. 415)
416. Pubis, ischiadic articular surface: continuous without marked angle change (0); marked step formed by a proximal posterior directed surface and a more distal posterodorsal oriented surface (1). (Carballido et al., 2019:ch. 416)
417. Pubis, proximal symphysis: merges with the pubic shaft (0); forms a marked ventromedially directed process (1). (Carballido et al., 2019: ch. 417)
418. Anterior caudal vertebrae, deep and marked fossa on anterior aspect of the transverse process (prcdf): absent (0); present (1). (New character)
419. Apex of the convexity of the posterior articulation on anterior and middle caudal vertebrae: concentrical or slightly displaced above the centrum midline (0); strongly displaced upward, so that the apex of the posterior articulation is flushed to the level of the dorsal margin of the centrum (1). (Santucci and Arruda Campos, 2011: character 235).
420. Articular facets of the prezygapophyses on anterior and middle caudal vertebrae: normal, not expanded (0); wide, with a dorsal and a ventral expansion or protuberance (1). (Santucci and Arruda Campos, 2011: character 237).
421. Prezygapophyses curved downward on anteriormost caudal vertebrae: absent (0); present (1) (Santucci and Arruda Campos, 2011: character 238).
422. Postzygapophyses located on the anterior half of the centrum on anterior and middle caudal vertebrae: absent (0); present (1). (Santucci and Arruda Campos, 2011: character 239).
423. Length proportions of the prezygapophyses with respect to the centrum length in middle caudal vertebrae: less than 40%(0); between 40-50%(1); more than 50% (2). (Santucci and Arruda Campos, 2011: character 241).
424. Haemal arches with double articular facets set in a concave posterodorsal surface: absent(0); present (1). (Modified from Santucci and Arruda Campos, 2011: character 240).
425. Haemal arches, distal portion of the articular process with lateral bulge:absent(0); present (1).
426. Haemal arches, articular area: with no well-developed posterior protuberance below (0); with well-developed posterior protuberance below (1).
427. Anterior caudal vertebrae, neural arch, SPOL development: null (0); poorly developed causing the articular facet of the postzygapophysis to project slightly from the midline (1); strongly developed forming a bony process that supports the articular facet of the postzygapophysis (2).
428. Middle caudal vertebrae, neural arch, SPOL development: null (0); poorly developed causing the articular facet of the postzygapophysis to project slightly from the midline (1); strongly developed forming a bony process that supports the articular facet of the postzygapophysis (2).
429. Anterior caudal vertebrae, central, height difference between the anterior and posterior articular face: null (0); present but little developed (1); present and strongly developed (2).
430. Middle caudal vertebrae, central, height difference between the anterior and posterior articular face: null (0); present but little developed (1); present and strongly developed (2).
431. Posterior caudal vertebrae, central, height difference between the anterior and posterior articular face: null (0); present but little developed (1); present and strongly developed (2).

Synapomorphy List

The following list of unambiguous synapomorphies were obtained from the pool of 42,857 most parsimonious trees that excluded identified unstable taxa.

*Plateosaurus*:

All trees:

Char. 23: 1 --> 0

Char. 44: 2 --> 1

Char. 60: 1 --> 0

Char. 64: 1 --> 0

Char. 65: 1 --> 0

Char. 82: 1 --> 0

Char. 96: 1 --> 0

Char. 99: 1 --> 0

Char. 103: 1 --> 0

Char. 115: 1 --> 0

Char. 186: 1 --> 0

Char. 237: 1 --> 0

*Mussaurus*:

All trees:

Char. 86: 0 --> 1

*Antetonitrus*:

All trees:

Char. 163: 0 --> 1

Char. 368: 0 --> 1

*Chinshakiangosaurus*:

All trees:

No autapomorphies:

*Isanosaurus*:

Some trees:

Char. 351: 0 --> 1

Char. 357: 0 --> 1

*Gongxianosaurus*:

Some trees:

Char. 122: 1 --> 0

*Amygdalodon*:

Some trees:

Char. 177: 0 --> 1

*Lessemsaurus*:

All trees:

Char. 391: 0 --> 1

*Tazoudasaurus*:

All trees:

Char. 371: 0 --> 1

Some trees:

Char. 105: 1 --> 0

Char. 173: 1 --> 0

Char. 175: 2 --> 0

Char. 181: 0 --> 1

Char. 259: 1 --> 2

Char. 275: 1 --> 0

Char. 276: 1 --> 0

Char. 306: 0 --> 1

Char. 329: 0 --> 2

Char. 356: 0 --> 1

*Vulcanodon*:

All trees:

Char. 250: 0 --> 1

Char. 386: 1 --> 0

Some trees:

Char. 396: 1 --> 0

*Shunosaurus*:

All trees:

Char. 83: 0 --> 1

Char. 108: 0 --> 1

Char. 113: 0 --> 1

Char. 114: 0 --> 2

Char. 133: 0 --> 1

Char. 145: 0 --> 1

Char. 165: 0 --> 1

Char. 176: 02 --> 1

Char. 210: 1 --> 0

Char. 268: 0 --> 1

Char. 309: 1 --> 0

Char. 329: 0 --> 1

Char. 357: 0 --> 1

Some trees:

Char. 156: 0 --> 1

Char. 193: 0 --> 1

*Barapasaurus*:

All trees:

Char. 177: 0 --> 1

Char. 179: 0 --> 1

Char. 256: 2 --> 1

Char. 368: 0 --> 1

Char. 415: 0 --> 1

Some trees:

Char. 193: 0 --> 1

Char. 273: 0 --> 1

Char. 275: 1 --> 0

Char. 276: 1 --> 0

*Patagosaurus*:

All trees:

Char. 175: 2 --> 0

Char. 181: 0 --> 1

Char. 259: 1 --> 2

Char. 303: 1 --> 0

Some trees:

Char. 193: 1 --> 0

Char. 196: 1 --> 0

Char. 363: 1 --> 0

*Cetiosaurus*:

All trees:

Char. 131: 01 --> 2

Char. 177: 0 --> 2

Char. 194: 0 --> 1

Char. 222: 1 --> 2

Char. 281: 0 --> 1

Char. 342: 0 --> 1

Some trees:

Char. 124: 0 --> 1

Char. 142: 0 --> 1

Char. 156: 0 --> 1

Char. 182: 0 --> 1

Char. 196: 1 --> 0

Char. 412: 0 --> 1

*Losillasaurus*:

All trees:

Char. 179: 0 --> 1

Char. 188: 0 --> 1

Char. 203: 0 --> 1

Char. 228: 0 --> 1

*Turiasaurus*:

All trees:

Char. 123: 0 --> 1

Char. 137: 0 --> 1

Char. 145: 0 --> 1

Char. 151: 0 --> 1

Char. 166: 1 --> 0

Some trees:

Char. 129: 0 --> 1

*Omeisaurus*:

All trees:

Char. 101: 0 --> 1

Char. 145: 0 --> 1

Char. 194: 0 --> 1

Char. 309: 1 --> 0

Char. 346: 0 --> 1

Char. 358: 1 --> 0

Char. 385: 0 --> 1

Char. 414: 0 --> 2

Char. 416: 0 --> 1

Some trees:

Char. 193: 0 --> 1

*Mamenchisaurus*:

All trees:

Char. 2: 0 --> 1

Char. 15: 1 --> 0

Char. 60: 1 --> 0

Char. 65: 1 --> 0

Char. 151: 0 --> 1

Char. 170: 0 --> 1

Char. 195: 1 --> 2

Char. 204: 0 --> 1

Char. 223: 0 --> 1

Char. 224: 0 --> 2

Char. 230: 0 --> 3

Char. 259: 1 --> 2

Char. 281: 0 --> 1

Char. 350: 0 --> 1

Char. 375: 0 --> 1

Some trees:

Char. 193: 01 --> 0

*Jobaria*:

All trees:

Char. 128: 0 --> 1

Char. 145: 0 --> 1

Char. 163: 1 --> 0

Char. 175: 2 --> 1

Char. 207: 2 --> 1

Char. 275: 1 --> 0

Char. 276: 3 --> 0

Some trees:

Char. 129: 0 --> 1

Char. 193: 0 --> 1

*Haplocanthosaurus*:

All trees:

Char. 149: 0 --> 1

Char. 154: 2 --> 0

Char. 157: 0 --> 1

Char. 176: 2 --> 1

Char. 203: 0 --> 1

Char. 206: 0 --> 1

Char. 231: 0 --> 1

Char. 255: 0 --> 1

Char. 414: 0 --> 1

Some trees:

Char. 129: 0 --> 1

Char. 180: 0 --> 1

Char. 202: 1 --> 0

Char. 227: 0 --> 1

Char. 278: 01 --> 2

Char. 279: 0 --> 1

*Camarasaurus*:

All trees:

Char. 23: 1 --> 0

Char. 59: 0 --> 1

Char. 137: 0 --> 1

Char. 151: 0 --> 1

Char. 163: 1 --> 0

Char. 166: 1 --> 0

Char. 195: 1 --> 2

Char. 276: 3 --> 1

*Bellusaurus*:

All trees:

Char. 230: 0 --> 3

Char. 274: 0 --> 2

Char. 356: 0 --> 1

Some trees:

Char. 223: 0 --> 1

Char. 224: 0 --> 2

Char. 329: 1 --> 0

Char. 361: 1 --> 0

*Europasaurus*:

All trees:

Char. 18: 1 --> 0

Char. 38: 1 --> 0

Char. 45: 0 --> 1

Char. 79: 0 --> 1

Char. 132: 0 --> 1

Char. 136: 0 --> 1

Char. 138: 0 --> 1

Char. 169: 1 --> 2

Char. 175: 2 --> 0

Char. 194: 1 --> 0

Char. 202: 1 --> 0

Char. 203: 0 --> 1

Char. 217: 1 --> 0

Char. 279: 0 --> 1

Char. 414: 2 --> 1

*Tehuelchesaurus*:

All trees:

Char. 159: 1 --> 0

Char. 179: 0 --> 1

Char. 181: 0 --> 1

Char. 192: 1 --> 0

Char. 195: 1 --> 0

Char. 207: 2 --> 1

Char. 333: 1 --> 0

Char. 358: 1 --> 0

*Tastavinsaurus*:

All trees:

Char. 201: 1 --> 0

Char. 205: 0 --> 1

Char. 350: 0 --> 1

*Brachiosaurus*:

Some trees:

Char. 37: 0 --> 1

Char. 44: 0 --> 1

Char. 169: 1 --> 0

Char. 305: 1 --> 0

Char. 351: 0 --> 1

Char. 361: 1 --> 0

*Giraffatitan*:

Some trees:

Char. 27: 0 --> 1

Char. 177: 0 --> 2

Char. 184: 0 --> 1

Char. 276: 3 --> 1

Char. 282: 1 --> 0

*Abydosaurus*:

Some trees:

Char. 12: 1 --> 0

Char. 62: 1 --> 0

Char. 77: 1 --> 0

Char. 92: 1 --> 0

Char. 104: 0 --> 1

Char. 141: 1 --> 0

*Cedarosaurus*:

Some trees:

Char. 240: 1 --> 0

Char. 251: 0 --> 1

Char. 260: 0 --> 1

Char. 302: 1 --> 0

Char. 357: 0 --> 1

*Venenosaurus*:

All trees:

No autapomorphies:

*Sauroposeidon*:

All trees:

Char. 121: 0 --> 1

Char. 165: 0 --> 1

Char. 197: 0 --> 1

Char. 236: 0 --> 1

Char. 282: 1 --> 0

Some trees:

Char. 164: 1 --> 2

*Lusotitan*:

Some trees:

Char. 187: 0 --> 1

Char. 314: 0 --> 1

*Padillasaurus*:

All trees:

No autapomorphies:

*Galveosaurus*:

All trees:

Char. 131: 2 --> 0

Char. 134: 0 --> 1

Char. 142: 1 --> 2

Char. 143: 1 --> 0

Char. 168: 0 --> 1

Char. 207: 2 --> 1

Char. 251: 0 --> 1

*Erketu*:

All trees:

No autapomorphies:

*Euhelopus*:

All trees:

Char. 107: 2 --> 1

Char. 108: 1 --> 0

Char. 111: 1 --> 0

Char. 113: 1 --> 0

Char. 123: 1 --> 0

Char. 131: 1 --> 0

Char. 132: 0 --> 1

Char. 145: 0 --> 1

Char. 285: 1 --> 0

Char. 289: 1 --> 0

Char. 336: 0 --> 1

Char. 354: 2 --> 1

*Qiaowanlong*:

All trees:

No autapomorphies:

*Phuwiangosaurus*:

All trees:

Char. 131: 1 --> 3

Char. 143: 1 --> 0

Char. 329: 0 --> 2

Char. 336: 0 --> 1

*Ruyangosaurus*:

All trees:

Char. 131: 2 --> 3

Char. 170: 1 --> 2

Char. 181: 0 --> 1

Char. 182: 1 --> 0

Char. 184: 1 --> 0

Char. 217: 1 --> 0

Char. 279: 0 --> 1

Char. 280: 1 --> 0

Char. 306: 0 --> 1

Char. 360: 0 --> 1

*Ligabuesaurus*:

All trees:

Char. 148: 1 --> 0

Char. 174: 1 --> 2

Char. 299: 1 --> 2

Char. 305: 1 --> 0

Char. 385: 0 --> 1

Some trees:

Char. 172: 0 --> 2

*Chubutisaurus*:

All trees:

Char. 163: 1 --> 0

Char. 201: 1 --> 0

Char. 346: 0 --> 1

*Wintonotitan*:

Some trees:

Char. 177: 1 --> 0

*Andesaurus*:

All trees:

Char. 226: 0 --> 1

Char. 232: 0 --> 1

Char. 233: 0 --> 1

Char. 354: 1 --> 2

*Epachthosaurus*:

All trees:

Char. 163: 1 --> 0

Char. 178: 1 --> 0

Char. 179: 2 --> 0

Char. 216: 0 --> 1

Char. 240: 0 --> 1

Char. 403: 2 --> 1

Some trees:

Char. 226: 0 --> 1

*Rinconsaurus*:

All trees:

Char. 141: 1 --> 0

Char. 274: 1 --> 2

Char. 430: 0 --> 1

*Pitekunsaurus*:

All trees:

Char. 124: 1 --> 0

*Narambuenatitan*:

All trees:

Char. 56: 1 --> 0

Char. 121: 1 --> 2

Char. 182: 1 --> 0

Char. 194: 1 --> 0

Char. 197: 1 --> 0

Char. 224: 3 --> 2

Char. 236: 2 --> 1

Char. 247: 0 --> 1

Char. 255: 0 --> 1

Char. 298: 1 --> 0

Char. 347: 1 --> 0

Char. 358: 1 --> 0

Char. 360: 0 --> 1

Char. 412: 1 --> 0

Char. 426: 1 --> 0

Char. 427: 2 --> 0

*Muyelensaurus*:

All trees:

Char. 121: 1 --> 2

Char. 170: 1 --> 2

Char. 171: 0 --> 1

Char. 195: 1 --> 0

Char. 236: 2 --> 1

Char. 281: 1 --> 0

Char. 428: 0 --> 1

Char. 429: 0 --> 1

*Gondwanatitan*:

All trees:

Char. 124: 1 --> 0

Char. 125: 3 --> 0

Char. 135: 2 --> 1

Char. 188: 0 --> 1

Char. 301: 1 --> 0

Char. 307: 1 --> 0

1. *rionegrinus*:

All trees:

No autapomorphies:

*Arrudatitan*:

All trees:

Char. 424: 0 --> 1

*Punatitan*:

All trees:

Char. 253: 1 --> 2

Char. 412: 1 --> 0

*Overosaurus*:

All trees:

Char. 143: 0 --> 1

Char. 161: 1 --> 0

Char. 256: 1 --> 2

Char. 422: 2 --> 1

*Bravasaurus*:

All trees:

Char. 121: 1 --> 2

Char. 140: 1 --> 2

Char. 174: 0 --> 1

Char. 254: 1 --> 0

*Trigonosaurus*:

All trees:

Char. 159: 1 --> 3

Char. 205: 0 --> 1

Char. 229: 0 --> 1

*Uberabatitan*:

All trees:

Char. 131: 1 --> 0

Char. 159: 1 --> 3

Char. 174: 0 --> 1

Char. 178: 1 --> 0

Char. 195: 1 --> 0

Char. 201: 1 --> 0

Char. 253: 1 --> 0

Char. 348: 0 --> 1

Char. 356: 0 --> 1

Char. 418: 0 --> 1

Char. 424: 0 --> 1

*Futalognkosaurus*:

All trees:

No autapomorphies:

*Drusilasaura*:

All trees:

Char. 232: 0 --> 1

*Puertasaurus*:

All trees:

Char. 251: 0 --> 3

*Bonitasaura*:

All trees:

Char. 38: 1 --> 0

Char. 181: 0 --> 1

Char. 248: 0 --> 1

Char. 320: 1 --> 0

Char. 374: 1 --> 0

*Quetecsaurus*:

All trees:

No autapomorphies:

*Petrobrasaurus*:

All trees:

Char. 235: 0 --> 2

Char. 301: 0 --> 1

Some trees:

Char. 303: 1 --> 0

*Baurutitan*:

All trees:

Char. 419: 0 --> 1

Char. 420: 0 --> 1

Char. 424: 0 --> 1

*Argentinosaurus*:

All trees:

Char. 177: 0 --> 1

*Patagotitan*:

All trees:

Char. 184: 1 --> 0

*Mendozasaurus*:

All trees:

Char. 158: 1 --> 0

Char. 426: 0 --> 1

Char. 427: 0 --> 1

Some trees:

Char. 422: 0 --> 1

*Dreadnoughtus*:

All trees:

Char. 271: 1 --> 0

Char. 275: 1 --> 0

Char. 276: 3 --> 0

Char. 290: 0 --> 1

Char. 303: 1 --> 2

Char. 311: 0 --> 1

Char. 313: 1 --> 0

Char. 314: 0 --> 1

Char. 329: 0 --> 2

Char. 350: 0 --> 1

*Malawisaurus*:

All trees:

Char. 149: 2 --> 1

Char. 193: 1 --> 0

Char. 251: 3 --> 0

Char. 418: 0 --> 1

Some trees:

Char. 169: 1 --> 2

Char. 197: 1 --> 0

Char. 286: 1 --> 0

*Notocolossus*:

All trees:

Char. 161: 1 --> 0

Char. 170: 1 --> 0

Char. 236: 2 --> 1

Char. 245: 1 --> 0

Some trees:

Char. 158: 1 --> 3

*Nemegtosaurus*:

All trees:

No autapomorphies:

*Tapuiasaurus*:

All trees:

Char. 193: 1 --> 0

Char. 206: 2 --> 1

Some trees:

Char. 158: 3 --> 0

*Isisaurus*:

All trees:

Char. 197: 1 --> 0

Some trees:

Char. 131: 3 --> 1

Char. 141: 1 --> 0

Char. 194: 2 --> 0

Char. 208: 1 --> 0

Char. 242: 2 --> 0

Char. 271: 1 --> 0

Char. 273: 1 --> 0

Char. 286: 1 --> 0

Char. 311: 0 --> 1

Char. 328: 1 --> 0

Char. 346: 0 --> 1

*Alamosaurus*:

All trees:

Char. 108: 2 --> 1

Char. 160: 3 --> 2

Char. 287: 0 --> 1

Some trees:

Char. 158: 3 --> 1

Char. 169: 1 --> 2

Char. 184: 2 --> 1

Char. 217: 1 --> 0

Char. 225: 0 --> 1

Char. 227: 0 --> 1

Char. 257: 0 --> 1

Char. 258: 0 --> 1

Char. 281: 1 --> 2

Char. 301: 0 --> 1

Char. 303: 1 --> 0

Char. 348: 0 --> 1

Char. 368: 0 --> 1

Char. 385: 0 --> 1

*Rapetosaurus*:

All trees:

Char. 259: 1 --> 2

*Opisthocoelicaudia*:

All trees:

Char. 151: 0 --> 1

Char. 174: 1 --> 0

Char. 177: 1 --> 2

Char. 182: 0 --> 1

Char. 185: 0 --> 1

Char. 208: 1 --> 0

Char. 225: 0 --> 1

Char. 260: 1 --> 2

Char. 273: 1 --> 0

Char. 274: 1 --> 0

Char. 346: 0 --> 1

Char. 360: 1 --> 0

*Saltasaurus*:

All trees:

Char. 125: 3 --> 2

Char. 194: 2 --> 1

Char. 223: 2 --> 1

Char. 227: 0 --> 1

*Neuquensaurus*:

Some trees:

Char. 370: 1 --> 0

*Histriasaurus*:

Some trees:

Char. 157: 0 --> 1

*Amazonsaurus*:

All trees:

Char. 244: 0 --> 1

*Zapalasaurus*:

Some trees:

Char. 232: 0 --> 1

Char. 236: 3 --> 1

Char. 241: 0 --> 1

Char. 245: 1 --> 0

*Lavocatisaurus*:

All trees:

Char. 127: 1 --> 0

Char. 306: 0 --> 1

*Comahuesaurus*:

All trees:

Char. 169: 1 --> 2

Char. 170: 1 --> 2

Char. 197: 0 --> 1

Char. 218: 1 --> 0

Char. 306: 0 --> 2

*Cathartesaura*:

All trees:

Char. 244: 0 --> 1

Char. 417: 1 --> 0

*Limaysaurus*:

All trees:

No autapomorphies:

*Rayososaurus*:

All trees:

No autapomorphies:

*Rebbachisaurus*:

Some trees:

Char. 234: 1 --> 0

*Katepensaurus*:

All trees:

Char. 206: 0 --> 1

*Tataouinea*:

Some trees:

Char. 223: 2 --> 1

Char. 225: 0 --> 1

Char. 232: 0 --> 1

*Nigersaurus*:

Some trees:

Char. 235: 1 --> 0

Char. 255: 0 --> 1

Char. 282: 1 --> 0

Char. 410: 1 --> 0

*Demandasaurus*:

Some trees:

Char. 181: 1 --> 0

Char. 249: 1 --> 0

*Suuwassea*:

All trees:

Char. 1: 0 --> 1

Char. 101: 1 --> 2

Char. 105: 0 --> 1

Char. 169: 1 --> 2

Char. 230: 2 --> 1

Char. 303: 1 --> 2

*Dicraeosaurus*:

All trees:

Char. 145: 0 --> 1

*Amargasaurus*:

All trees:

Char. 76: 0 --> 1

Char. 146: 0 --> 1

Char. 179: 0 --> 2

Char. 309: 1 --> 0

Char. 332: 0 --> 1

*Brachytrachelopan*:

All trees:

No autapomorphies:

*Apatosaurus*:

All trees:

Char. 77: 1 --> 0

Char. 129: 0 --> 1

Char. 284: 0 --> 1

Char. 303: 1 --> 2

Char. 309: 1 --> 0

Char. 341: 0 --> 1

Char. 355: 0 --> 1

Char. 378: 0 --> 1

Char. 384: 0 --> 1

*Diplodocus*:

All trees:

Char. 157: 0 --> 1

Char. 206: 0 --> 1

*Barosaurus*:

All trees:

Char. 218: 1 --> 0

Char. 303: 1 --> 0

*Ninjatitan*:

All trees:

Char. 230: 3 --> 2

Char. 231: 0 --> 1

Char. 417: 0 --> 1

*Xianshanosaurus*:

All trees:

Char. 138: 0 --> 1

Char. 168: 1 --> 0

Char. 355: 1 --> 0

*Huabeisaurus*:

All trees:

Char. 125: 3 --> 2

Char. 143: 1 --> 0

Char. 181: 0 --> 1

Char. 206: 2 --> 1

Char. 347: 0 --> 1

Char. 360: 0 --> 1

Char. 368: 0 --> 1

*Daxiatitan*:

All trees:

Char. 201: 1 --> 0

Char. 250: 0 --> 1

Char. 357: 0 --> 1

*Malarguesaurus*:

All trees:

Char. 251: 0 --> 2

MCF-PVPH 916:

All trees:

No autapomorphies:

MCF-PVPH 917:

All trees:

Char. 247: 0 --> 1

Char. 255: 0 --> 1

Char. 256: 2 --> 1

Char. 259: 1 --> 0

Node 105 (Sauropoda):

All trees:

Char. 158: 13 --> 0

Char. 159: 1 --> 0

Char. 160: 3 --> 0

Char. 161: 1 --> 0

Char. 163: 1 --> 0

Char. 167: 1 --> 0

Char. 170: 1 --> 0

Char. 228: 1 --> 0

Char. 245: 1 --> 0

Char. 252: 1 --> 0

Char. 412: 1 --> 0

Node 106:

All trees:

Char. 386: 0 --> 1

Node 107:

All trees:

Char. 352: 0 --> 1

Some trees:

Char. 122: 0 --> 1

Char. 196: 0 --> 1

Char. 353: 0 --> 1

Char. 396: 0 --> 1

Node 108:

All trees:

Char. 199: 0 --> 1

Char. 207: 0 --> 1

Char. 299: 0 --> 1

Char. 310: 0 --> 1

Node 109:

Some trees:

Char. 131: 1 --> 0

Char. 140: 0 --> 1

Char. 165: 1 --> 0

Char. 207: 1 --> 2

Char. 302: 0 --> 1

Char. 312: 0 --> 1

Char. 316: 0 --> 1

Char. 379: 0 --> 1

Char. 392: 0 --> 1

Char. 398: 0 --> 1

Node 110 (Eusauropoda):

All trees:

Char. 172: 0 --> 1

Char. 195: 0 --> 1

Char. 276: 1 --> 3

Char. 280: 0 --> 1

Some trees:

Char. 361: 1 --> 0

Node 111:

Some trees:

Char. 133: 1 --> 0

Char. 167: 0 --> 1

Char. 335: 0 --> 1

Char. 353: 0 --> 1

Char. 358: 0 --> 1

Char. 366: 0 --> 1

Char. 367: 0 --> 1

Char. 395: 0 --> 1

Char. 399: 0 --> 1

Node 112:

All trees:

Char. 0: 0 --> 1

Char. 66: 0 --> 1

Char. 213: 1 --> 2

Some trees:

Char. 142: 0 --> 1

Char. 182: 0 --> 1

Char. 273: 0 --> 1

Node 113:

All trees:

Char. 157: 0 --> 1

Char. 206: 0 --> 1

Char. 230: 0 --> 2

Some trees:

Char. 193: 0 --> 1

Node 114:

All trees:

Char. 21: 1 --> 0

Char. 317: 0 --> 1

Char. 344: 0 --> 1

Char. 372: 0 --> 1

Some trees:

Char. 156: 0 --> 1

Node 115:

All trees:

Char. 124: 0 --> 1

Char. 125: 1 --> 2

Char. 155: 0 --> 1

Char. 163: 0 --> 1

Char. 166: 0 --> 1

Node 116:

All trees:

Char. 114: 0 --> 1

Char. 123: 0 --> 1

Char. 126: 01 --> 3

Char. 139: 0 --> 1

Char. 140: 1 --> 0

Char. 141: 0 --> 1

Char. 160: 0 --> 3

Char. 173: 1 --> 0

Char. 174: 2 --> 1

Char. 179: 0 --> 1

Char. 206: 0 --> 2

Char. 219: 1 --> 0

Some trees:

Char. 135: 0 --> 12

Node 117 (Neosauropoda):

All trees:

Char. 170: 0 --> 1

Char. 245: 0 --> 1

Char. 362: 0 --> 1

Char. 375: 0 --> 1

Some trees:

Char. 373: 0 --> 1

Node 118:

All trees:

Char. 414: 0 --> 2

Char. 416: 0 --> 1

Some trees:

Char. 209: 1 --> 0

Node 119 (Macronaria):

All trees:

Char. 131: 1 --> 2

Char. 156: 1 --> 0

Char. 194: 0 --> 1

Char. 206: 0 --> 2

Char. 336: 0 --> 1

Char. 341: 0 --> 1

Some trees:

Char. 164: 0 --> 1

Char. 282: 0 --> 1

Node 120:

All trees:

Char. 75: 0 --> 1

Char. 76: 0 --> 1

Char. 106: 1 --> 0

Char. 113: 0 --> 1

Char. 228: 0 --> 1

Char. 252: 0 --> 1

Char. 321: 0 --> 1

Char. 322: 0 --> 1

Char. 324: 1 --> 0

Char. 330: 0 --> 1

Some trees:

Char. 44: 1 --> 0

Char. 329: 1 --> 2

Node 121:

All trees:

Char. 354: 2 --> 1

Node 122:

All trees:

Char. 260: 0 --> 1

Node 123:

All trees:

Char. 255: 0 --> 1

Char. 256: 2 --> 1

Char. 259: 1 --> 0

Node 124 (Somphospondyli):

All trees:

Char. 166: 1 --> 0

Char. 396: 1 --> 0

Char. 415: 1 --> 0

Char. 416: 1 --> 0

Some trees:

Char. 329: 2 --> 0

Node 125:

All trees:

Char. 208: 0 --> 1

Node 126:

All trees:

Char. 123: 0 --> 1

Char. 135: 1 --> 2

Char. 141: 0 --> 1

Char. 212: 0 --> 1

Char. 230: 0 --> 1

Node 127:

Some trees:

Char. 1: 1 --> 2

Char. 4: 0 --> 1

Char. 5: 0 --> 1

Char. 89: 0 --> 1

Char. 100: 0 --> 1

Char. 164: 1 --> 0

Char. 170: 1 --> 0

Char. 173: 0 --> 1

Char. 180: 0 --> 1

Char. 195: 1 --> 2

Char. 202: 1 --> 0

Char. 204: 0 --> 1

Char. 227: 0 --> 1

Char. 299: 1 --> 2

Char. 306: 0 --> 1

Node 128:

All trees:

Char. 168: 0 --> 1

Char. 177: 0 --> 1

Char. 184: 0 --> 1

Node 129:

All trees:

Char. 123: 1 --> 0

Char. 130: 0 --> 1

Char. 137: 0 --> 1

Char. 364: 0 --> 1

Node 130:

All trees:

Char. 121: 0 --> 2

Char. 131: 2 --> 1

Char. 195: 1 --> 2

Char. 413: 1 --> 0

Node 131:

All trees:

Char. 157: 0 --> 1

Node 132:

All trees:

Char. 107: 1 --> 2

Char. 111: 0 --> 1

Char. 125: 2 --> 3

Char. 171: 0 --> 1

Char. 292: 0 --> 1

Char. 313: 0 --> 1

Char. 336: 1 --> 0

Node 133:

All trees:

Char. 333: 1 --> 0

Char. 361: 1 --> 0

Node 134:

All trees:

Char. 141: 1 --> 0

Char. 147: 0 --> 1

Char. 194: 1 --> 0

Char. 213: 2 --> 3

Char. 236: 0 --> 2

Char. 242: 0 --> 2

Node 135:

All trees:

Char. 125: 3 --> 2

Char. 128: 0 --> 1

Char. 145: 0 --> 1

Node 136:

All trees:

Char. 165: 0 --> 1

Some trees:

Char. 162: 1 --> 0

Char. 284: 0 --> 1

Char. 347: 0 --> 1

Node 137:

All trees:

Char. 274: 0 --> 1

Char. 306: 0 --> 2

Node 138:

All trees:

Char. 305: 1 --> 0

Char. 310: 1 --> 0

Some trees:

Char. 123: 1 --> 0

Char. 138: 0 --> 1

Char. 250: 0 --> 1

Char. 342: 0 --> 1

Node 139:

All trees:

Char. 131: 2 --> 3

Char. 158: 1 --> 3

Char. 242: 0 --> 2

Char. 256: 2 --> 1

Char. 259: 1 --> 0

Some trees:

Char. 226: 1 --> 0

Char. 422: 0 --> 1

Node 140:

All trees:

Char. 282: 1 --> 0

Some trees:

Char. 108: 1 --> 2

Char. 121: 0 --> 1

Char. 133: 0 --> 1

Char. 147: 0 --> 1

Char. 150: 1 --> 0

Char. 178: 0 --> 1

Char. 179: 01 --> 2

Char. 221: 0 --> 1

Char. 230: 1 --> 3

Char. 251: 0 --> 3

Char. 260: 0 --> 1

Char. 269: 0 --> 1

Char. 303: 0 --> 1

Char. 384: 0 --> 1

Node 141:

All trees:

Char. 240: 0 --> 1

Char. 413: 1 --> 0

Node 142:

All trees:

Char. 227: 0 --> 1

Node 143:

All trees:

Char. 71: 0 --> 1

Char. 158: 1 --> 3

Char. 306: 2 --> 1

Node 144:

All trees:

Char. 247: 1 --> 0

Char. 249: 0 --> 3

Char. 253: 0 --> 1

Node 145:

All trees:

Char. 150: 0 --> 1

Char. 204: 0 --> 1

Char. 242: 0 --> 2

Char. 426: 0 --> 1

Char. 427: 0 --> 2

Some trees:

Char. 422: 0 --> 1

Node 146:

All trees:

Char. 127: 1 --> 0

Char. 171: 1 --> 0

Char. 236: 0 --> 2

Char. 247: 0 --> 1

Some trees:

Char. 177: 1 --> 0

Node 147:

All trees:

Char. 256: 2 --> 1

Char. 301: 0 --> 1

Char. 349: 1 --> 0

Node 148:

All trees:

Char. 184: 1 --> 2

Char. 421: 0 --> 1

Char. 422: 1 --> 2

Char. 427: 2 --> 1

Node 149:

All trees:

Char. 240: 0 --> 1

Char. 254: 0 --> 1

Node 150:

All trees:

Char. 420: 0 --> 1

Node 151:

All trees:

Char. 171: 0 --> 1

Char. 181: 0 --> 1

Char. 418: 0 --> 1

Node 152:

All trees:

Char. 170: 1 --> 2

Char. 194: 1 --> 2

Node 153:

All trees:

Char. 425: 0 --> 1

Node 154:

All trees:

Char. 195: 1 --> 0

Node 155:

All trees:

Char. 235: 0 --> 2

Char. 243: 0 --> 1

Char. 246: 0 --> 1

Some trees:

Char. 290: 0 --> 1

Node 156:

All trees:

Char. 248: 0 --> 1

Node 157:

All trees:

Char. 243: 0 --> 1

Char. 358: 1 --> 0

Node 158:

All trees:

Char. 422: 0 --> 1

Node 159:

All trees:

Char. 240: 0 --> 1

Char. 275: 1 --> 0

Char. 276: 3 --> 0

Char. 303: 1 --> 2

Some trees:

Char. 169: 1 --> 2

Node 160:

All trees:

Char. 205: 0 --> 1

Char. 222: 1 --> 2

Char. 304: 0 --> 1

Char. 422: 1 --> 0

Some trees:

Char. 13: 1 --> 2

Char. 63: 0 --> 1

Char. 79: 0 --> 1

Char. 86: 0 --> 1

Char. 107: 2 --> 3

Char. 182: 1 --> 0

Char. 360: 0 --> 1

Node 161:

All trees:

Char. 173: 0 --> 1

Node 162:

All trees:

Char. 232: 0 --> 1

Char. 287: 0 --> 1

Char. 290: 0 --> 1

Char. 297: 0 --> 1

Char. 301: 0 --> 1

Char. 350: 0 --> 1

Some trees:

Char. 289: 0 --> 1

Char. 311: 0 --> 1

Node 163:

All trees:

Char. 156: 0 --> 1

Char. 157: 0 --> 1

Char. 257: 0 --> 1

Char. 261: 0 --> 1

Char. 348: 0 --> 1

Char. 369: 1 --> 0

Node 164:

Some trees:

Char. 198: 0 --> 1

Char. 229: 0 --> 1

Node 165:

All trees:

Char. 240: 1 --> 0

Char. 243: 0 --> 1

Char. 249: 0 --> 1

Char. 412: 1 --> 0

Node 166:

All trees:

Char. 123: 0 --> 1

Char. 146: 1 --> 0

Char. 163: 1 --> 0

Char. 166: 1 --> 2

Char. 195: 1 --> 0

Char. 216: 0 --> 1

Char. 235: 0 --> 1

Char. 242: 0 --> 1

Char. 258: 0 --> 1

Char. 276: 3 --> 2

Some trees:

Char. 0: 1 --> 0

Char. 1: 1 --> 0

Char. 9: 0 --> 1

Char. 10: 0 --> 1

Char. 21: 0 --> 2

Char. 22: 1 --> 2

Char. 24: 0 --> 1

Char. 57: 0 --> 1

Char. 60: 1 --> 2

Char. 80: 0 --> 1

Char. 98: 1 --> 2

Char. 99: 1 --> 3

Char. 102: 0 --> 1

Char. 105: 1 --> 0

Char. 106: 1 --> 0

Char. 107: 1 --> 3

Char. 108: 0 --> 3

Char. 110: 0 --> 1

Char. 113: 0 --> 1

Char. 236: 0 --> 3

Char. 262: 0 --> 1

Char. 263: 0 --> 1

Node 167:

All trees:

Char. 116: 0 --> 1

Char. 412: 0 --> 1

Node 168:

Some trees:

Char. 179: 0 --> 1

Char. 230: 0 --> 4

Char. 337: 0 --> 1

Node 169:

All trees:

Char. 283: 0 --> 1

Node 170:

All trees:

Char. 112: 0 --> 1

Char. 117: 0 --> 1

Char. 303: 1 --> 0

Node 171:

All trees:

Char. 190: 0 --> 1

Some trees:

Char. 142: 0 --> 2

Char. 170: 1 --> 2

Char. 411: 0 --> 1

Node 172:

All trees:

Char. 408: 0 --> 1

Some trees:

Char. 181: 0 --> 1

Char. 193: 0 --> 1

Char. 234: 0 --> 1

Node 173:

All trees:

Char. 3: 0 --> 1

Char. 28: 0 --> 1

Char. 36: 1 --> 0

Char. 45: 0 --> 1

Char. 52: 0 --> 1

Char. 88: 0 --> 1

Char. 107: 3 --> 2

Char. 171: 0 --> 2

Node 174:

All trees:

Char. 13: 1 --> 2

Char. 151: 0 --> 1

Char. 190: 0 --> 1

Char. 230: 0 --> 2

Char. 259: 1 --> 2

Char. 275: 1 --> 0

Char. 276: 2 --> 0

Char. 334: 0 --> 1

Char. 343: 1 --> 0

Char. 344: 1 --> 0

Char. 345: 0 --> 1

Char. 385: 0 --> 1

Some trees:

Char. 268: 1 --> 0

Node 175:

All trees:

Char. 203: 0 --> 1

Node 176:

All trees:

Char. 37: 0 --> 1

Char. 49: 0 --> 1

Char. 124: 1 --> 0

Char. 126: 12 --> 3

Char. 155: 1 --> 0

Char. 160: 1 --> 0

Node 177:

All trees:

Char. 27: 0 --> 1

Char. 29: 0 --> 1

Char. 30: 0 --> 1

Char. 46: 0 --> 1

Char. 109: 1 --> 0

Char. 118: 1 --> 3

Char. 135: 1 --> 2

Char. 154: 2 --> 3

Char. 156: 1 --> 0

Char. 181: 0 --> 1

Char. 231: 0 --> 1

Char. 238: 0 --> 1

Char. 239: 0 --> 1

Char. 244: 0 --> 1

Some trees:

Char. 142: 1 --> 2

Char. 180: 1 --> 0

Char. 193: 0 --> 1

Char. 227: 0 --> 1

Node 178:

All trees:

Char. 141: 0 --> 1

Char. 144: 0 --> 1

Char. 232: 0 --> 1

Char. 233: 0 --> 1

Char. 241: 0 --> 1

Char. 249: 0 --> 2

Char. 250: 0 --> 1

Char. 256: 2 --> 3

Node 179:

All trees:

Char. 127: 1 --> 0

Char. 151: 1 --> 0

Char. 157: 1 --> 0

Char. 164: 1 --> 2

Data Matrix (TNT format)

Xread

431 104

*Plateosaurus* 0000000-00000000000001000000000100000000010010?00000?0000??000000000000000??00000?00000??0?00000000000000?0000----000000000?00-1000101000?000?00001000100-0000000000-1-0-0000102?0000000000-??0000000--00--0000000????0?000?00?00?000000??0?000-??00-0---00000?02?010000????0???0000000000000000?0?00???001000000000000?0000000010000000000000?0000000?000000010000000000?000000000000000000000100000010000000000000000?0?000?0000-000000000000

*Mussaurus* 0000000-000000000000010100?00?010?0000000?002???000??0?0????????????????????????????1?1??0?00000??0?00??0100?0----[0 1][0 1]00?0000??0-??0010100000000???0??0??00-?000000000-1-0-0000????0??0000001-??0000000--00--0000000???10?000?00????000000??0?0?0-??00-0---0?000?02?01000?????0???0000000000000000?0??????0?10000000000?0?000??000000?0[0 1]00000000?00?000??0000000100?0000000?0?0???000?0??000??000???000010000???00?0???00?00000?0000-000000000000

*Antetonitrus* ??????????????????????????????????????????????????????????????????????????????????????????????????????????????????????????0??0-????10????????0?????????0?-?00000001121-0-00001022000000000?-??0000000--01--000000???????0???00???????000??0?0?0-??00-????00000?02?0?000?????0????0?11010??0000?????????????0000?0??0110?000??000?00?0???????????????????????00?000100000010001?0100?????????001???1?00??0???????????????00000????0-000000000000

*Chinshakiangosaurus* ????????????????????????????????????????????????????????????????????????????????????0?0????00?????0??1?10?000?????01?0????0??0-?????0?????????????????????????????????????????????????????????????????????????0??????0?????????????????????????????????????????????????????????????????????????????????????????????????????????????????????????????????????????00??????????????????????????????????????????????????????????????????????????????

*Isanosaurus* ??????????????????????????????????????????????????????????????????????????????????????????????????????????????????????????1??0-????10????????0?????????????0???????0-1-?-0?0????????????00?-????????10?11??00?0??????????????????????????????????????????????????????????????????0011000??000??????????????????????????????????????????????????????????????????11110010001?????????????????????????????????????????????????????????????????????

*Gongxianosaurus* ???????????????1??????????????????????????????????????????????????????????????????????????????????????????11?1????21??????0??0-????10????????0?????????????0???????????0-????????0???????0?-??????????????????????????????0?00??????0000???????????????????000????01000?????0????0011000??000?00?0?0???????1000?0?00?01?00000?0?????????????????????????????????111???0?????01??0??????????0000???1?00?00000100000?????????????????????0?0?0?00

*Amygdalodon* ?????????????????????????????????????????????????????????????????????????????????????????????????????????111?100-021?0????1????????1????????00?????0???????0????0????1-?-????????1????0??0?-??????0???????????01???0????????00????????00?????????????????00000????0???0??????????????????????????????????????????????????????????????????????????????????????????????????????????????????????????????????????????????????????????0-???????0000?

*Lessemsaurus* ?????????????????????????????????????????????????????????????????????????????????????????????????????????????????????????00010-1000??1000000?0???01?0010?-?000000010-1-0-0000102200000000?1-???000000--11--000010???????????00???????????????????????????????????????????????????001101000000000?0???????01110010[0 1]001110000??000?00?0??00?000?00???????0000000?00000000001000????0?00?0000??00????1??01100??00??????????0000???00??????????????

*Tazoudasaurus* ?????????????????????????????????00?10?????????01?????0--???????????????????????????100????0011???0??00100110100?001?0??001000-1000001000000100000?0??-000?000000110-000-000001000000100001-??011?00100111-000020??00???????001000000000000001001?00-0---00000?020020000??1100??10000000???????0?00??????001101101101?10100?100?001?0101?2????00000000?10?000??0101010000?000?0?00010010100?00???011?000?0001010?1???0??00000?1??0-000?0?000000

*Vulcanodon* ??????????????????????????????????0?????????????????????????????????????????????????????????????????????????????????????????????????????????????????????????????????????-????????????????????????????????????????????1????0?00?00?????00???0?100?????????01?????????????????00??10??????0??????????????????1101?0???1110100????0???????1??????0000000?0100?????0101??????1000?000??00?1000010?1??00100?0100?011001??????????0??????00000000?0??

*Shunosaurus* 01000?1-000011011-0001110000000??000001001??2000001101100000100011000000000??0000011100?0?000?101?1100?1011111000121?02000100101000001000000100001?010?00-1010000110-1-1-000112210000000001-??011101[0 1]0-11[0 1]?000020000010-0001001000000000000001001000-0---0?000?02001?00---1110101001300010??000010000010000110110100101010001000001101011100000100000???00000?0?11100110000101?1000?0?10?0011111001110??111?11111001000?00000?1000?000000000000

*Barapasaurus* ???????????????????????????????????????????????????????????????????????????????????????????????????????????101????01?0???01000-??0?0?0?0???0100??0?????00-?00000011??0?1-???0????1010000?0?-??011100100111-000020???01100?0?00?00???000000000100??00?????000???010???0?0--1?00?01100000000000000?00??????0??101101001110100???????????0110000?010000000100000000111000100?010111100???1?10???01??0?????????1??11?0??????00000?01?0?000000000000

*Patagosaurus* 1???0?1-00??11????????????????????0???????????????????????????????1??????????????????0????????????1????101110100-001?0??001001010000?00000001011001010100-?000000110-001-000112000000110001-??01100100-11--000020??002??0?0???????000000000??1??1????????0?000?02002?0??????00??110130001?000?00100??????00?101001001110100????????????110000?01000000010000000011100010000001???00?????????101??011????????????????????0000[0 1]?1??0?000????00000

*Cetiosaurus* ?????????????????????????????????????????????????????????????????????????????????????????????????????????????????????????01011010002?00000001011001010100-?010000110-001-0?00????2000010001-???1101000-11--0000[1 2]0?10??????0?002??0??0000000?0?0-1000-0---00000?0200?000??????????001100001??0???1????01000??10?10??0???????????????????110000?0?0000??1?0?000??011????1??100????????????????????????????????????????????????11[0 1]??0-000?00000000

*Losillasaurus* ??????????????????????????????????????????????????????????????????????10?????0????1????????????????1???1??????????????????10120??00100000000101100?01??0?-?11111011100110100?????00100?00010100111011001111100120???????????00??????10200?10[0 1]100??000000000????????1????????????????????????????????????????1011010?????1??????????????01??0??010?0?00?11?00????????????????????????????????????????????????????????????00???????0-000????0?0??

*Turiasaurus* ?????????????????????0???0?????????????????????????????????????????????????????????0??????????????????????111100-00100????11120?010100000100101?01101011???11111011100010?-0?????0000010001000011101100111100?1200100???????00??????002000?0?1??????????????????????200?????????1???????????????????00??000?10110100????10011110000111????????????????????????????????????00??11???0101010?11?????11?????1?10?111???????00???????0-???????????0

*Omeisaurus* 1100001-000011011-10011100??000???001010011010001011011?000?1???11100?0?0??00?0?0010100000000?1???1101?101110100-0110040001112310000000[1 2]00010110011010?00-210000[2 3]11100110000101220010010001000?11111100111-000220010?2110100??1000000000000001001000-0---0?000?02001?00???11000011013000?0000?00000?001000011011010010101000100000011101100000010000000100100000111000000001011??00000101001111[0 1]01111100111111111001000?00001?2010-000?0?000000

*Mamenchisaurus* 1110001-0-0011101-10011100?0?00???00?010010010?010?1?111000?0???101?00???0??0?0???10100??0?001101111[0 1]00101110100-011?04000111231000[1 2]000200010110001010?100210011311100110110-01-200100?00010000110021001?1101022001002??0?00??1120000030000001001000-0---0?000?02002?0????1100?0110??0?0?1????00?0?00????0011011010011101001101?0001???11[0 1]00000??00000010000001011100010000101?11000001110?11?1??01?1?001111??1??????00?0000000000-000000000000

*Jobaria* 11000010100011111-?0101100?0000??10010100?101000101101110???10??11?000??00?00?0?0?1?1000000???????11000?01110100-001001?001??2111101?00000001001011010101-2[0 1]10110110-0[0 1]1-11011212000001000?---0111011001111000010010021101?1001000000000??000100?000-100000000?0200100????1?001011000010????0100000?0????001101101001110100011100001111111000?010000000110000000111000100?110111000?11111001111??01111001????????????00000001?0000?000000000000

*Haplocanthosaurus* ??????????????????????????????????????????????????????????????????????????????????????????????????????????????????????[1 2 3 4]??01012110101?00100001001001011101-01111101110011011011221000101000100001100110011101001201100211011?001000010001000001001000-100000000?120010???????10??110130211?000100?00??000??????????????????????????????1111000?01000000011000???01110001001???????????1??????????????????????????????????00001?1??0?000?0?00000?

*Camarasaurus* 11100010100011111-1010100[0 1]0000000100101000101000101101110001101?111000000000010000101000000111111111010?01110100-0210010001012110002?0[0 1]10100101100?01-?1012100111110-0010110-01-?00000100010000110121001111000220010?2110111001000000000000011001000-100000000?020010000--[0 1]0[0 1]0101101100010100100100000000001101[0 1]0100111010011111100111111100000110000101100000001110001001110111000010111001111[0 1]0011110?111111111000?00000001?2110?000000000000

*Bellusaurus* ????0??0100011??????????????????????10100???1??01??1??11000??010??????000??????????????0???????????1??0101110100-02100???0101211000200010000101100?010100-?10011111110110110?????000001000100001101110011110002201?0?2??0?1?00?1200000300000110?10000100000000????0?000?????????1121300010100100?00??????00?10110100111010????1????????110000?01100001011000000011101010001101?????????????????????????????????????????000001?0100-0000???00000

*Europasaurus* 1110001010001111??0010110200000??1001?0000100100101101110000101????[0 1]0000000111010?1010000?01111?1?1101010[0 1]010100012100?0?01012[0 1]10002100110111011001010100-?100111111101102101020?00000100010000110011001110100220010021?001?00?000??10000000010000000100000010?020010000--0-10??1101300110100100100000???00?101001011110100??11??11?01111210??0110000?0110??000?111000100?1101110??0101110?11?1?????1????1?1??11?????00000001?1110-00000?000000

*Tehuelchesaurus* ???????????????????????????????????????????????????????????????????????????????????????????????????????????????????????????????????[1 2 3]??????????????????????[0 1 2]100001110-00102100010?00101100010000100?0100111-000211??01[1 2 3]1???1?????????????????????????????????????????????????????1101300010100100111??????001100101011010100????????????1?????0011000?101100000001111000001??????????????????????????????????????????????0000???00??????????????

*Tastavinsaurus* ???????????????????????????????????????????????????????????????????????????????????????????????????????????????????????????????????????????????????????????100?1[2 3]?10-0[0 1]??????????0?00010001000011011100?10-001221??0?211011?0010[0 1]01010[1 2]00000110010000100000010011000100???0-1011??????????????????????????????????????????????????????111[0 1]10110110000101100000101111001?011101110110???????1111??111011011?100111?001???00001?2000-00001?000000

*Brachiosaurus* 1210??1?10??11???????????????000?10?1110?01010?01011??110??????????0?000000111??0?10??????????????12?10?0?0111000121?0???011?22??002?002000111??????1??????10011211100110000211220001010001000?111121001110010221?????????????????1??0??????????????????????????????????????????????????????????????????????10100011????????????????????????????????????????00011121001000???????????0???????????????????????????????0000000?????0?00000?0?????

*Giraffatitan* 121011101000111101101111010100000100101000100000101101110000101?111000000001110?00101000010111111212110?00011100012100?0001112210002[0 1]00200011111001010100-210011211100110100211222001010101000011[0 1]1210011100102210111211011?001000111010000001001000-100000010002001000?????10??11011000?10001001100000000021010011111101001111111110111121011011000010110000000112100100111011111001?111001111??0111?0111?1??11?0???0000000?12110-000000000000

*Abydosaurus* 12101110100001?10110?1110100?0???????????0??00?01011??1100001?0?????0?0?????100?????100??1010????212110110?1110???21?0?00?1??2???????????????0??????????????????????????????????????????????????????????????????1??????????000????111??0???????????????????0100?????00???????????????????????????????????????????1??????????????????????????????????????????0???1??1???0???????????????????????????????1?????????????000???????????????????0?00

*Cedarosaurus* ???????????????????????????????????????????????????????????????????????????????????????????????????????????????????????????????????????????????????????????100??[2 3]????????????????????????0?????1??????????????2?1???????????00?????11010??000?0?0?00010000?110000000100???????????0??00011???1011??00???00?210000111111??00?????????????????????????????????0???11210110????0???????????????1111??11?????111111100??????0000?1???0-0000???00000

*Venenosaurus* ?????????????????????????????????????????????????????????????????????????????????????????????????????????????????????????????????????????????????0?????????????????????????????????????????????????????????????????11???????00????1110-00??00?001000010000001000000?000?????????11?130001110?1??????????????????????1?10100????1?????????????101?0000101?000??????????????????????????????????????????11????????????????????11?1[0 1]0-0000???00000

*Sauroposeidon* ??????101???11?????????????????????????????????????????????????????????????????????????????????????[0 1 2]??0100011?000121?0???11112210?02000200001111001010100-?10011311121-111[1 2]000[1 2]??100001010?00001111[0 1]110111?000221011121?????00?????01010??001100000001000000100020010000--0-10??110??0001[1 2]000100?1000???0001101001?11110100??1?1?11???11???0?1011?000101100000?01121011001110111?10????????????????????????????????????00000?1???0-000000000000

*Lusotitan* ???????????????????????????????????????????????????????????????????????????????????????????????????????????????????????????????????????2???????????????????1????[1 2]?110??????????????????????1?0?????[1 2]??????????[1 2]?????????????00????1?10100000010?1000010000001000?000000?????10??1????????????????????????00?101???1[0 1]1?1?101??????????????????1011?0001011?00??????????????1101?????0??1110???????????????????????????????????????0-000000000000

*Padillasaurus* ???????????????????????????????????????????????????????????????????????????????????????????????????????????????????????????????????????????????????????????1????2????????????????0???????0?000????1???????????2?????????????00?00011101000?0010-??0001000???100?2????????????????????????????????????????????????????????????????????????????????????????????????????????????????????????????????????????????????????????????????0-0000????????

*Galveosaurus* ?????????????????????????????????????????????????????????????????????????????????????????????????????????????????????????01112110?000012??0??1200???1??????1??1111111011111??????00??0?000????01101[1 2]100111100021001012110???00???????01000?0?100?0???????00110????0?000?????1???1????????????1??????0000000?101[0 1]0100????????????????????????????????01011000????????????????????????????????????????????????????????????0000???????????00000000

*Erketu* ???????????????????????????????????????????????????????????????????????????????????????????????????????????????????????0021013-1011100020100111100101????????????????????????????????????????????????????????????01?????????????????????????????????????????????????????????????????????????????????1?????????????????????????????????????????????????????????????????????111?110???1?111?0????????????????????????????????????????????????????

*Euhelopus* 01100?10100011??1-10?[0 1]110[0 1]00000???00???????????0??????11?00??01?1???????????????????10000001??????1101011[0 1]010100-021004?0210[0 1]3-110001012000010?1?1111-1101110111311110011111-0[0 1]-31??0?10[0 1]11000?11102110111100022101113??01??????????????????????????????????????????????????????11013000?110?000?00??????00?101[0 1]0101??????????????????11100?10011000010110000100111100100011011101001?11?00?1111001?1100?1???11??0???0000000?0?00??????????????

*Qiaowanlong* ??????????????????????????????????????????????????????????????????????????????????????????????????????????????????????????1012111111?00?010011110110???????????????????????????????????????????????????????????????????????????????????????????????????????????????????????????????????????????????????????????????????????????????????1101?[0 1]1??0?0?010??000??????????????????????????????????????????????????????????????????2????????????????

*Phuwiangosaurus* ????0?????????????????????10?????100101????????0??????111??????????1000110?1100?0?1?????????????????0?????0211010121?0[2 3 4]??21012111113?0?201001110011010110?21[0 1]11131111001111?????110100101110000111121[0 1]011110002201???2????1???????1010100?00010-1?0001000100100110010000--0-10??110??00011001???????11???00?10?0010?1101110????????????112?0110110000101100000011121?01001111111011?????????????????????????????????????0000?02000-000000000000

*Ruyangosaurus* ?????????????????????????????????????????????????????????????????????????????????????????????????????????????????????????01113-????3???2?????1????10???????101?[1 3]31111001112?????310101000010000[0 1]11111001111000221??1?21?00??00?1???0??1000?????????????????0??????0?????????????1101300101100100[0 1]10??????00?101[0 1]0111??????????????????111?101???????????????010011210010111101??????????????????????????????????????????0000?1?????????????0???

*Ligabuesaurus* ???????????????????????????????????????????????????????????????????????????????????????????????????[0 1 2]??01000211010?21?0???01?1?????0??002??0???000??002101-?10011310111-111112022?100001010?000?11?111001111000221????????????????????????????????????????????????????????????????1113000111011???????????00210100021????????????????????????????????????????000011210?1001110111010?1???????1111011111101???????????????0000?1?????????????????

*Chubutisaurus* ???????????????????????????????????????????????????????????????????????????????????????????????????????????????????????????????????????????????????????????1????3?10-0011?1??????10??01?10?0000???1?10??10-00?22???11???????00100??0101000?0?10??0??-????00010????0?000?????10??111??000111001???????????011101001211??0110??111111???????????????0001011010000011210110011101??????????????????????1?10???????????????????????????????00000000

*Wintonotitan* ???????????????????????????????????????????????????????????????????????????????????????????????????????????????????????????????????????????????????????????1??[1 3]?31?121-?1?11[0 1]0?2?0?????????????????????1??????22???11???????00?????0?01000??????00??0????000100???0?0001????10??11???0001[1 2]10?1???????????????01001??1?10110??111??????????????????00?10?10?1?????????????????????????????????????????????????????????????????[0 1]?????????00000000

*Andesaurus* ???????????????????????????????????????????????????????????????????????????????????????????????????????????????????????????????????????????????????????????10?[1 3][1 3]311121-1111?????2?000010101000?111??1??1111000221???????????00102110102011000?0?0000010000?0100020010?0???0?11????????????????????????????????1??????????????????????????????101100001011001??????2??????1??????????????????????????????????????????????00001??0000????000??000

*Epachthosaurus* ???????????????????????????????????????????????????????????????????????????????????????????????????????????????????????????????????????????????????????????100?13110-1-11111??????000010201000?111211101111000221??113??1?????1130101030?000010010??010000131??0100010????0-11111???????????????????1??????1101100211?00?101-1111112--11??11?101??0?????????0000[1 2]121011001?101?10?111?????11111110111110111?011110111???0000?????00000100000000

*Rinconsaurus* ??????????????????????????????????????????????????????????????????????????????????????????????????????????02[2 3]??????10????1101300-?010102??001000001111100-?100313[0 1]0121-112100000201201102010000111111101111000221??11?????1?002????110300000210?10??01000303110020011101?00?11?11?213?0?110?1111?00111010011101[0 1]011??????????11?1112--11?011?101?0001101100101001121011101????????????????????????????00????????????????000010???00000100012221

*Pitekunsaurus* ?????????????????????????????????????0100?0???0?????0??????????????10001000011?00000??????????????????????022?01012100??0110030001010102??00110?001111100-?1003131?121-11?100000?????????010000???1???01??????2???111???????00??????10300000210?1???01000?0?11???0?11101?0???????1113?0?110011??????????????????????1000????????????????????????????????????0???112101???1???????????????????????????????????????????????????0???000001???1?2?0

*Narambuenatitan* ?020?0??????????????????????????????011?0?002010101?1?11000????????110?1000011?00100?????????????????????????????????????2101?????01?102?0?????????????????10[0 1]??3?0121-??????????0?2000010100000110?100?1??010221?1?????????00?1201010300000110-0?20010103031101[0 1]?0????????????????????????????1?0011101000?111011111110?????????????????????1011000?10???00?00111[1 2]???0011??????????????????????????????????????????????00000??00000001???00000

*Muyelensaurus* ????????????????????????????????????0?????002???????0??????????????1???0000?1???0?????????????????????01?002???????10????21013-0-0?1010200001100?0111??????1001?3??121-112210000???2001010?0000111101101?1-010221???????????00?????01?30?000110?00???100030311002001110??????????1113000100011??????1????0??10100121?????????????????????????10100001101100?0???1121011001??01??????????????????????????????????????????000011?00000001???12110

*Gondwanatitan* ??????????????????????????????????????????????????????????????????????????????????????????????????????????????????????????1000-????????1??????????????????3100?131?121?11210????201200?020101000111?110????0??221??113???10?0??????0103010?0210?1020010003031110?002100???0?1??????1?????????????????????01?10101110???????????????????11011?1????001??11001??????????????1101??????????????????????????????????????????00001????000012?0011000

*A*.*_rionegrinus* ????????????????????????????????????????????????????????????????????????????????????????????????????????????????????????????????????????????????????????????????????????????????????????????????????????????????????????????0??????0103010?0?100??20?1000?03111?????????????11?????13?0?110??1???????????01?111001111100111??????????????????????00?10011001??????????????1101111100111001??????????????????????????????????1????0111121001?0??

*Arrudatitan* ?????????????????????????????????????????????????????????????????????????????????????????????????????????????????????????????????????1?2???????????????????????????????????????????????????????????????????????????1????????00????????301?00?10-??20010003?31110?????1??????11???????????????????????????????????????????????????????????????????????????????00?1121?1?001??????????????????????????????????????????????00001????01111211111000

*Punatitan* ??????????????????????????????????????????????????????????????????????????????????????????????????????????????????????????101????0?????2??????0?0?11???????1003?31?121-112211???20120110201000011101110?11001?????111???????000????010301100110-002001??031312100?0???????0?11??????????????????????????????????????????????????????????????????????????????????????????????????????????????????????????????????????????00000????0????2?0111000

*Overosaurus* ?????????????????????????????????????????????????????????????????????????????????????????????????????????????????????????11013-0-??30102??001?010??111100-31003?300121-11221000021120??020?????1?1?01101?1-0?0221?11131101??001130?01?3010?0?10??0???????30311102001110???????????????????????????????????????????????????????????????111?111???????????????????????????????????????????????????????????????????????????00001????011011?????000

*Bravasaurus* ????????????????????????1?????????????????????????????1?100??????????????????????????????????????????????????????????????21013-0-1030102000021000111???0?-?10031310121-112210?1?2[0 1]12011020101001?120110111-01022101?1???????0??????01?30?00??10-?0???1????031?001?0???????0?11??1????????????????????????01211110121??????????????????1110111?01????????????0??1112101???1????11110?????????????????????????????????????00001???00????2???????0

*Trigonosaurus* ?????????????????????????????????????????????????????????????????????????????????????????????????????????????????????????11013-0-10101020?00110001111??00-310033310121-11220000220120010201000011121110111-0?1221?1??31101??0?01???011300000[1 2]1001?20010003031110100?????????????????????????????????????????????????????????????????????????????????????????????????????????????????????????????????????????????????????????1????001012???11000

*Uberabatitan* ?????????????????????????????????????????????????????????????????????????????????????????????????????????????????????????11013-0-1000102000011000?11???0[0 1]-?10?3331?121-112100012200?00??1??????????0110110-?1??2?0111?????0?0??????01030000021001?2001000303101010021001?00?11?11??????????????1?111110100??11????11????111????1?????????????101?000??0?1??1100111211110011101110100111001????????????????????1?????????????1???0?1100111012000

*Futalognkosaurus* ??????????????????????????????????????????????????????????????????????????????????????????????????????????????????????21011013-0-001010200001100001112001-3100333?0121-11??0000020120??01?????????101?011?-???2?1????31?01?????13????030??022?????0101110???????????????????????1?????????????????????????????????????????????????????11??1111??000011011001??01????????????????????????????????????????????????????????0000?????00000??????0??

*Drusilasaura* ???????????????????????????????????????????????????????????????????????????????????????????????????????????????????????????????0-????????????????????????????????????????????0?????????????????????????????????????????????????13?????301??221????0101?11????????????????????????1?130000?????????????????????????????????????????????????????????????????????????????????????????????????????????????????????????????????????????00000?????0??

*Puertasaurus* ?????????????????????????????????????????????????????????????????????????????????????????????????????????????????????????21013-0-????102????????0??112001-??0?3?31?12101??100010???????????????????????????????????????????????????????????????????????????3???????????????????????????????????????????????????????????????????????????????????????????????????????????????????????????????????????????????????????????????????????????????????

*Bonitasaura* ????????????????01??????????????????0?00000?2?????????1110???????????????????????????0?000????????2[2 3]?10100022101012100??011[0 1]???0-1?1??020000??000011121?1??1001[0 1]310121-11210[0 1]02[0 2]2?12?110101000011?11110111101?2210111?????????????101030?000210-002001?11003100020011001????11??1???????????????????1?????1???1?01??1110????????0??2??1??????????000110110010101112???1001?1011?010?1?0??1??101???1111101111????1???????00001????00000100012000

*Quetecsaurus* ???????????????????????????????????????????????????????????????????????????????????????????????????????????22?????2100?1?11?13-0-????102??001??00???1[1 2]00????0?3?3??121?1???0000???????????????????????????????????????????????????????3000?????????????????????????????????????????????????????1?11????????????????1????????????????????????????????????????????????????????????????????????????????????????????????????????????????????????0??

*Petrobrasaurus* ??????????????????????????????????????????????????????????????????????????????????????????????????????????02[2 3]?----2100????111??????????????????????????????100113?0121??????????10?20010[1 2]0000001?11110??1??0?0221??11???????01?????010300002210-0?0101010?0??0??????????????1??????????????????????1110?001111100121?????????????????????????????????????????000112101000111????????????????????????????????????????????0000?????0000000000000?

*Baurutitan* ????????????????????????????????????????????????????????????????????????????????????????????????????????????????????????????????????????????????????????????????????????????????????????????????????????????????????????010?0122300010300000210-10200100000310002001100???0-1111????????????????????????????????????????????????????????????????????????????????????????????????????????????????????????????????????????????1????00110101000000

*Argentinosaurus* ???????????????????????????????????????????????????????????????????????????????????????????????????????????????????????????????????????????????????????????1001?3?0121-1121?2121?10?001010?000?111121101111000221????[2 3]????????????????????????????????????????????????????????????????????????????????????????????????????????????????1?????????????????????????????????????0????10?????????????????????????????????????0000???????????????????

*Patagotitan* ??????????????????????????????????????????????????????????????????????????????????????????????????????????02[1 2]10100?100???21013-0-?010112000011100011???????10011310121-112102121100200100010000111121101111000221??11???????01[0 1]0311010300002210-0001011110031000200?100?????11??111130110200111?011?1??10111101101211110110??????????????????101100?1101100101011121011001????????0?????????????????????????????????????00001[0 1]?0000000000000000

*Mendozasaurus* ?????????????????????????????????????????????????????????????????????????????????????????????????????????????????????????1111??0-?0??102??0???000?1112001-?10?0?31?121-11??00010????0??01????????????0?1????????????????????0???3?101030000021000001010100?21??0200110????0?????11113000110011?????11????01?101101211??0?????11?????????????????????????????00001121010????10??1??0????????????11?1?1?????????1110121???0000?????00000100011000

*Dreadnoughtus* ??????????????????????????????????????????????????????????????????????????????????????????????????????????02210?01?100???11113-????30102??0011000?11?????-?10031310121-111?1?????1120010[1 2]?????0111[1 2]11101111000221??1131?????0?12300?10300000010000200100000310001000100???0-11101110000011001110001?11010??1101201211011101???????????11?2?11101000011011001?01011210110011101110???101100??111??01????????1?11?????????000011???00000100000000

*Malawisaurus* 1120?0???????1?????????????????????????????????????????????????0???????????????0???0100???????????1?0101??02?1????21?0???11013-??103?1021010110?001111000-?1003?311121-112210010??1200?020100001?01110?11110?02210?1????????011???0010300?0?0100????-10??0?01000100010????0-11??1?????????????00?001110??01?101100[1 2]110001?0??1??111????1??????????00111110010????1210???????1111010?????????????????????????????????????00001?????1000100000000

*Notocolossus* ???????????????????????????????????????????????????????????????????????????????????????????????????????????????????????????????????????????????????????0???10?3?30?121-11?000010???????????????????????????????????????????????????01030000011000000000-000??0??????????????11??1???????????????????????????10110121????????????????????????????????????????????????????????????????1011011111101011?1?01111001110121???0000?????000000?????000

*Nemegtosaurus* 00200??11-0?12?1[0 1]1?01[0 1]211110?0010101101?0?0120?01010?1111??2111011?1100010??00100?1?101??0011??01?12?101000321010121?0???????????????????????????????????????????????????????????????????????????????????????????????????????????????????????????????????????????????????????????????????????????????????????????????????????????????????????????????????????????????????????????????????????????????????????????????001???????????????????????

*Tapuiasaurus* 002?0?111?0012?10110?2211200??0111011?1000?0???01011?1?11???11111???1?0000???1110???101?????0??1??12?1010003[2 3]101012100????1??????????1?2?0??11?????????????1000?31?121-1112???????120?000?????0110??110111????12???11?????????????????????????????????????????????????????????????????????????10?10?1?????????1?????110011?????1????????????????????????????????1?2???????????1??????????????????????????1110?111????001???????????????????????

*Isisaurus* ??????????????????????????????????????????????????????????????????????????????????????????????????????????????????????????10?3-10001?10210101000000110100-?10033300121-1112000122?1200?0001000011100100111-0?1220?1?13110?0?????????1030?00001000000-10000131000200110????0-11?01011300000001000?00???????1?101111?11101??????????????1100111?0110001111101?????????????????????????????????????????????????????????????0000[0 1]?2??00000000000000

*Alamosaurus* ??????????????????????????????????????????????????????????????????????????????????????????????????????????0311010121?0??111?13-10?030102?01011000?1112000-310?11210121-1122000?22?1200001010000111211101111001221?111[2 3]110010012231011030000011000020-100001310001110100???0-111111113000020011110101110?101?1110102111001111?1111112--??????1???000?1111100?1???112???111??11111111????????111111111?11011?1?111?0??????00001[0 1]???00000000000000

*Rapetosaurus* 00200?1?1?1012?10110?12?1?10?0011?00001?0?0120?01010?1111??21111???11?0100???1110?1?101??0011?????12?1010003?11-0121?040?11013-10003?102100011010?0110100-?100333[0 1]0121-11221001021120000201000011120110111-011221????3?????????????0?03???????00??20???????3???020?21???????????1110000???????00?0011????01?101211211?00110????1?1????111010110110001??11?01010111?111101????????10????????????10?????00?????????????00100001?20?00000100000000

*Opisthocoelicaudia* ???????????????????????????????????????????????????????????????????????????????????????????????????????????????????????????????????????????????????????1?0?100??300121-11[1 2]21-00-?21200102110000111211101111001220??1131?0?00??2??10010-01000110010[0 2]0-100001-100020002001000-1111100000000?0011110111110111111112102111011111?1111112--111011110110001?111011001011210111011111110111101101111111001111101111011110122???00001?20?0?0000?0000000

*Saltasaurus* ?????????????????????????????00?????1010???020?01??????????????????0100010??1?????10????????????????????????????????????1?1012-10003000200101000000110100-?11131300121-1122100?2211200002010000111111101111001221????311010?11?13?01103010001100??20-1000013100021101101?0??11?01110000000001011011?110??11?111210211101111???????????111011110110001111100110111121011111111111001?????????????????????????????????????00001????00000000000000

*Neuquensaurus* ??????????????????????????????????????????????????????????????????????????????????????????????????????????????????????????1013-100030002??00??00000110100-?111?1300121-11221001221120000201000?111211101111001221????31?0???11?230001030??001100??20-1000013???021?011?1?0??????111000?000001011011?1????11?111210211101111????????????11011?101?000111110011011112101111?1111?10001101101??111??0????00?1??????????????00001???000000000000000

*Histriasaurus* ???????????????????????????????????????????????????????????????????????????????????????????????????????????????????????????????????????????????????????????111?1??????????1??????0?010??0?1???0???00101111-01?020????????????????????????????????????????????????????????????????????????????????????????????????????????????????????????????????????????????????????????????????????????????????????????????????????????1?????????????????????

*Amazonsaurus* ???????????????????????????????????????????????????????????????????????????????????????????????????????????????????????????????????????????????????????????1????111????10????????0????????????????0?1?0111??1???????????????0????????000[0 1]?0?3???0?1111???10000002?1?00??????10????????????????????????????????????????????????????????????0?????????????????????????????????????????????????????????????????????????????????0??????????00000000

*Zapalasaurus* ??????????????????????????????????????????????????????????????????????????????????????????????????????????????????????????11?2110?01001?00????????????????????????????????????????????????????????????????????????????????????????0001001001110?011?00---1000000201100???????????????????????????????????????????????????????????????????????????000?0?11?00000???????????2?????????????????????????????????????????????????0????1-000????00000

*Lavocatisaurus* 0020001011?011?0??1??22?????0?????????????????????????10011?????????????????????????11110000??????2300?10003311-012110?000110210-?01?01100001000000010?0???1????1????????????????????????????????????????????????11?????????0???????0140000?????0????1???100000020110011?1??????11012121001001???????????00?10110111111010????????????????????????????????????????????????2101111????????????0??001??????????????????112????1?????????????00000

*Comahuesaurus* ????????????????????????????????????????????????????????????????????????????????????????????????????????????????????0??????????????????????????????????????11??11110-0210220?????0?1??1??0?0000???00111111-?1?02??????????0?0??210010140000??10?0????????10000002011?0??????10??1?????????????00?00?1????0??10110121??????????????????????????01010000011?000001211000100?2?????????????????????????????????????????????0?0?0??101-?00?00000000

*Cathartesaura* ?????????????????????????????????????????????????????????????????????????????????????????????????????????????????????????011021??0010011??0???000?0010?0?-?????????????????????????????????????????????????????????????????????????0?140??0131000?1111???10000002?1??0???????????1?121110011?1????????????????????????????????????????????????????????????????012110001??????????????????????????????????????????????????????????0-000????00000

*Limaysaurus* ?????????????1?01-????2?1???000??11?-0011111-001??-10111011?20?????100101010110?101???????????????????????03311-112111???01102110001001100001000000010?00-?110111110-0210111012220121011001000011000101111-1110201?0???????[1 2]0?1[0 2][0 1]000014000013100001?01000100000020110011?1??10??1101211100110100100?11???0?1101001011110100????????????11????0010110000110100?0?211000?001210??1????11???0??100??01111?1?????????????11?0??01????1-000?00000000

*Rayososaurus* ??????????????????????????????????????????????????????????????????????????????????????????????????????????????????????????????????????????????????????????????11?????????????????????????????????????????????????????????????????????????????????????????????????????????????????1012111001001??????????????????????????????????????????????????????????????????2?1????????????????????????????????????????????????????????????????000?000?????

*Rebbachisaurus* ???????????????????????????????????????????????????????????????????????????????????????????????????????????????????????????????????????????????????????????110111110-02?012?012?201?1111001000111100101111-111020?????????????????????????0?3?????1101?00???????????????????????110121?1001001???????????0??1000??[0 2]??????????????????????????????11100??????????????????????????????????????????????????????????????????1111???????????????????

*Katepensaurus* ?????????????????????????????????????????????????????????????????????????????????????????????????????????????????????????0110211[0 1]001001100001000000010?0???110?1?1???021011?0????012111100100001?100101??1-11?120?1?????????00?????101400?11310?0?1101000???????????????????????????????????????????????????????????????????????????????????????????????????????????????????????????????????????????????????????????????1?10?????1-000?????????

*Tataouinea* ?????????????????????????????????????????????????????????????????????????????????????????????????????????????????????????????????????????????????????????????????????????????????????????????????????????????????????211111?1121010101401011310?10110100011000002?11??????????????????????????????????????????????????????????????????11?000?????111?0????1?????????????????????????????????????????????????????????????????1??????0??????0000?

*Nigersaurus* 0020001011101100??1??2211??0000??11?-0011110-?11----0111011?2??????100100010??0?1?101012000000????23001?0003311-1121112??0110211[0 1]101001100001020001010?00-211?111110-02101200120?0111111001000111100101111-1100201?00???1?????????0?0140??103?0?0?11010001?0000120110011?11010??1101212100000100?00??????00?10000101???????????????????1100??0???111??????100?0?211010100????????????1???????????????????????????????1?211011????1????0???00000

*Demandasaurus* ?0?00???????????????????????????????????????????????????????????????????????????????10120?????????2??2?1?0033?????2111??0?11021???010011000010200??010?0?-?11???1?10-02?????0????0?1?0110?1000111100101?11-?1?02011????????????210???140001131001?1101000010000??0??00??????10???????????????????????????????????????????????????????????????????111????1?100001211010100???????????????????????????????????????????????111??????1-000?00000000

*Suuwassea* 01210????1???2??????????1??01???????00?00??12100?0??1??0?10????????0????0?0????????0?1?11?????????2??2???102???????10??0001112110?01000100101010?0?0?-?1???11???11?????10212?12-???????????000?????????1?????????1100???????0?????????1????????????????????????????????????????????00000?000?????????????00????2????????????????????????????????????????????0????????????????????00???????????????????01???????????????????0???????????????????

*Dicraeosaurus* 0021?????1???2??0?1?????????100???0?011000112100111111?????1?0???10001110100010?111011011?????????23?11?0002311-012100110111023100010000-1-1101101001-?1112010220110-1-1-112-12-20001011001---110000100111-100020110021?1?0?0?11200000200001310010100100000000002012?0?1?11?00??11000000???00100??0??????001101101001110??????????????111000001100000??00100?00011100010011101110101111110??111??111?1011????11?????????00001?0[0 1]00?00000000000?

*Amargasaurus* ?????????????2?01-1?????????100??10?01100011210011111??????????????0011101001?0?1110??????????????????????????????????11011102310001000?-1-1101000101-?110?010220110-1-1-112-12-20021011001---?10?00100111-0000201??02??1????????????0?0??0??????0100??????0???020??????????????1??00??????0?1???????????001101101001010?00????????????111001???????????????00001110?0?001???????00??1??????????????????????????????????00001?0??????00000?0?0?

*Brachytrachelopan* ?????????????????????????????????????????????????????????????????????????????????????????????????????????????????????????11?0?3100?1???0-?-??????000????1?2010220110-1-1-1?2-12-?0001011001---110000100111-1000201???????????????????????????????????????????????????????????????????????????????????????????????????????????????????????????????????????????????????????????????????????????????????????????????????????000???????????????????

*Apatosaurus* 0020001?111112011-11?221100101100?0010100011201010110110010020?01?000010000000001010?1?0001???????23011?0003301-01210?31021112210101?00201-1102100001-?111310000?110?0210110-12-0000011000111?111100100111-010020110021?11120?11200100210001311110101100000000002012?0111111001011000000100011001?000000?001101201001010100111100001111111000011000001?0010000001111001001110111000011111011111011111101?1111?111000100100001?[0 1]001-000000000000

*Diplodocus* 00200010111112011-1112211001011001001010001120101011011001002010110000100001010010101100001101101?23011?1003301-012100310?1112210001?00201-0112110001-?101310100111??0210110-12-20000110001111111100100111-010120110021111120?11200100211101311111101100021000003012?0111111001011000000011001001?0000000001101101001110100???????????1111000011000000?00100000011100010111101?10000111110011110011111011111?111100?1001000011?001-000000000000

*Barosaurus* ??????????????????????????????????????????????????????????????????????????????????????????????????????????????????????3??11112210?01?00?00-1112010001-?1013100001110-0210110-12-0000011000100?111100100111?01002011002????0?0?11200100211101311111101100021000003012?011?11?00?01????000????????1??00????00??0?00?0????????????????????11???0?11?0???????100?000111000???11??????00???1???????????????01????????????????0000???001?000000000000

*Ninjatitan* ??????????????????????????????????????????????????????????????????????????????????????????????????????????????????????????101??????????2???????????????????1????3??????1????????????????????????????????????????????????????00?????1??2100???1000?????????0??????????????????????1?130001100?1?????????????????????????????????????????????????????????????????????????00??????1??0??????????????????????????????????????????1???1-?00?????????

*Xianshanosaurus* ??????????????????????????????????????????????????????????????????????????????????????????????????????????02110101?1??????1????0?00?0?0?001010???0??1010?0?10011?1112??101?12?[0 1]11??200?01??000?111?21?01?11??0221??0?[1 2 3]1?????0?????1?002?100021???02001000?0?10?????[1 2]????????00????????????????00?11??????????????????????????????????????????00?0?0?0101100?0?001120001000?1???????????????????????????????????????????????????????0???0?0?????

*Huabeisaurus* ??????????????????????????????????????????????????????????????????????????????????????????????????????????02110101?100???[1 2]11121101?1001????010?00?111??1???1????311????11????????[0 1]???11??0?00001?10?11?1?1????121??113??01?[1 2]001110?01000[0 1]000210000200100000010002001000???0?100??1013???1?10?100?10??????????????????????1???????????????????101000001011001??0011210??0111101?1110?????????????????????????????????????0?001??000-000?0?000000

*Daxiatitan* ?????????????????????????????????????????????????????????????????????????????????????????????????????????????????????????21113-0-001??02??0010111011???0???100??31112??11??12?????1[1 2]0??????000?1?1?21??1?0????221???????????0??????1?03??00???001?[1 2]?01?0??1??0??????????????00???1013001111001001???????????????????????????????????????????????????????????01001121?1?00??????????????????????????????????????????????????????????0???0?0?????

*Malarguesaurus* ????????????????????????????????????????????????????????????????????????????????????????????????????????????????????????????????????????????????????????????????????????????????????????????????????????????????????????????00??????10100000010?0000010000?21001100{0 1}100?????????????????????????????????????????????????????????????????????????????????????0?????210{0 1}??????????????????????????????????????????????????????1????0-0000???00000

MCF-PVPH 916 ????????????????????????????????????????????????????????????????????????????????????????????????????????????????????????????????????????????????????????????????????????????????????????????????????????????????????????????00???????????00??????????????00010011000000?????????????????????????????????????????????????????????????????????????????????????????????????????????????????????????????????????????????????????1????0-0?00???00000

MCF-PVPH 917 ????????????????????????????????????????????????????????????????????????????????????????????????????????????????????????????????????????????????????????????????????????????????????????????????????????????????????????????00????0010?0000001000?000101000010011000?00?????????????????????????????????????????????101??????11?????????????????????????????????????????????????????????????????????????????????????????????1????0-0000???00000

;
